# An overview of meta-analyses on radiomics: more evidence is needed to support clinical translation

**DOI:** 10.1186/s13244-023-01437-2

**Published:** 2023-06-19

**Authors:** Jingyu Zhong, Junjie Lu, Guangcheng Zhang, Shiqi Mao, Haoda Chen, Qian Yin, Yangfan Hu, Yue Xing, Defang Ding, Xiang Ge, Huan Zhang, Weiwu Yao

**Affiliations:** 1 Department of Imaging, Tongren Hospital, Shanghai Jiao Tong University School of Medicine, Shanghai, 200336 China; 2 Department of Social and Behavioral Sciences, Harvard T.H. Chan School of Public Health, Boston, MA 02115 USA; 3 Department of Orthopedics, Shanghai Sixth People’s Hospital, Shanghai Jiao Tong University School of Medicine, Shanghai, 200233 China; 4 Department of Medical Oncology, Shanghai Pulmonary Hospital, Tongji University School of Medicine, Shanghai, 200433 China; 5 Department of General Surgery, Pancreatic Disease Center, Ruijin Hospital, Shanghai Jiao Tong University School of Medicine, Shanghai, 200025 China; 6 Department of Pathology, Shanghai Sixth People’s Hospital, Shanghai Jiao Tong University School of Medicine, Shanghai, 200233 China; 7 Department of Radiology, Ruijin Hospital, Shanghai Jiao Tong University School of Medicine, Shanghai, 200025 China

**Keywords:** Radiomics, Quality improvement, Systematic review, Meta-analysis

## Abstract

**Objective:**

To conduct an overview of meta-analyses of radiomics studies assessing their study quality and evidence level.

**Methods:**

A systematical search was updated via peer-reviewed electronic databases, preprint servers, and systematic review protocol registers until 15 November 2022. Systematic reviews with meta-analysis of primary radiomics studies were included. Their reporting transparency, methodological quality, and risk of bias were assessed by PRISMA (Preferred Reporting Items for Systematic reviews and Meta-Analyses) 2020 checklist, AMSTAR-2 (A MeaSurement Tool to Assess systematic Reviews, version 2) tool, and ROBIS (Risk Of Bias In Systematic reviews) tool, respectively. The evidence level supporting the radiomics for clinical use was rated.

**Results:**

We identified 44 systematic reviews with meta-analyses on radiomics research. The mean ± standard deviation of PRISMA adherence rate was 65 ± 9%. The AMSTAR-2 tool rated 5 and 39 systematic reviews as low and critically low confidence, respectively. The ROBIS assessment resulted low, unclear and high risk in 5, 11, and 28 systematic reviews, respectively. We reperformed 53 meta-analyses in 38 included systematic reviews. There were 3, 7, and 43 meta-analyses rated as convincing, highly suggestive, and weak levels of evidence, respectively. The convincing level of evidence was rated in (1) T2-FLAIR radiomics for IDH-mutant vs IDH-wide type differentiation in low-grade glioma, (2) CT radiomics for COVID-19 vs other viral pneumonia differentiation, and (3) MRI radiomics for high-grade glioma vs brain metastasis differentiation.

**Conclusions:**

The systematic reviews on radiomics were with suboptimal quality. A limited number of radiomics approaches were supported by convincing level of evidence.

**Clinical relevance statement:**

The evidence supporting the clinical application of radiomics are insufficient, calling for researches translating radiomics from an academic tool to a practicable adjunct towards clinical deployment.

**Graphical Abstract:**

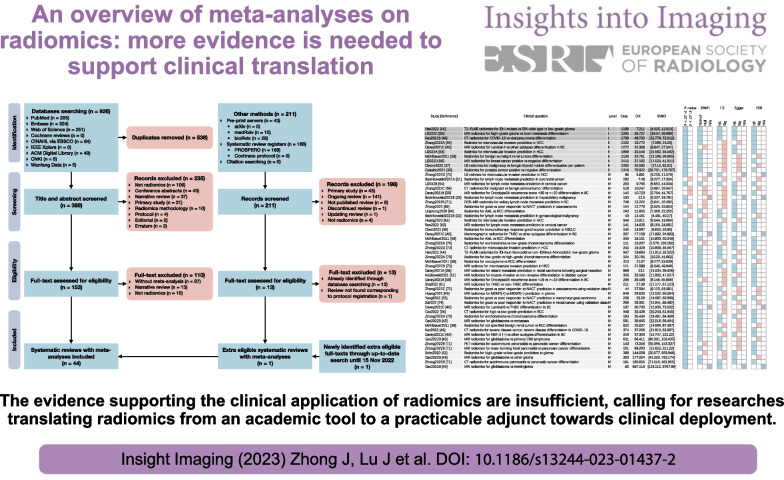

**Supplementary Information:**

The online version contains supplementary material available at 10.1186/s13244-023-01437-2.

## Key points

- The systematic reviews on radiomics studies were insufficient in reporting, suboptimal in methodological quality, and with high risk of bias.
- The meta-analyses covered a wide range of clinical questions, while only three of them were rated as convincing level of evidence.
- More radiomics investigation is necessary to allow clinical translation of radiomics to a practicable adjunct toward clinical deployment.

**Supplementary Information:**

The online version contains supplementary material available at 10.1186/s13244-023-01437-2.

## Introduction

A decade has passed since the concept of radiomics was raised [1]. The concept of radiomics is based on an assumption that medical images contain information of disease-specific processes that are undetectable to naked eye [2]. Radiomics, a high-throughput methodology that extract large amounts of imaging biomarkers from medical images, is believed to be one of the most promising approaches for enhancing the existing images into deeper mineable data to support clinical decision-making [1–6]. The rapidly evolving field of radiomics has attracted considerable interest, with a plethora of primary radiomics studies being published [7, 8]. Radiomics seems to potentially have a huge impact on clinical routine at first sight, but so far, little to none of these encouraging findings have served as evidence supporting these research tools translating into clinical application [9–13].

Primary radiomics studies are the sources of information for clinical evidence, while the systematic reviews and meta-analyses provide integration or synthesis of evidence with higher precision from conflicting results, and address questions that cannot be asked in individual studies [14]. Although an increasing number of systematic reviews and meta-analyses are published in various medical fields, including radiomics [15], it is still unclear how far radiomics is from current research to clinical application [9–13]. There were systematic reviews attempt to cover a wide range of topics in radiomics [16, 17]. However, the number of published primary radiomics studies was too large to summarize in one single systematic review [16], and the evidence level rating of current radiomics was out of the aim of a methodological systematic review [17]. Nevertheless, the overview of systematic reviews is a relatively new type of publication that attempts to provide a broader evidence synthesis highlighting the knowledge gaps, biases, and priorities for future research, which helps clinical practitioners and policy-makers interpret the results of higher-level pieces of evidence in radiomics [18–20].

Therefore, our overview of systematic reviews of primary radiomics studies is aimed at assessing the study quality and the evidence level supporting radiomics application in clinical settings.

## Methods

### Protocol and registration

This overview of meta-analysis has been prospectively registered on PROSPERO (CRD42021272746), and the review protocol is available as Additional file 1: Note S1. The ethical approval was not required due to the nature of the study. The overview of meta-analysis was conducted as per guidelines [19–22]. The corresponding checklists are supplied in Additional file 1. The literature search, study selection, data extraction, and quality assessment were duplicated by two independent reviewers (J.Z. and either Y.H., Y.X., X.G., or D.D.). The disagreements were resolved by consults with a third independent reviewer (G.Z., S.M., H.C., Q.Y., G.Y., H.Z. or W.Y.). The data analysis was performed by a reviewer (J.Z.) under supervision of a statistical expert (J.L.).

### Literature search and study selection

A systematic search was performed to identify systematic reviews with meta-analysis concerning on the radiomics applications for diagnostic, predictive, or prognostic purposes. The search strategy was tested for feasibility with the variations of the terms “radiomics”, “systematic review” and “meta-analysis”. The full formal search was performed until 30 September 2022 and was updated until 15 November 2022. We searched the peer-reviewed electronic databases (PubMed, Embase, Web of Science, Cochrane reviews via Cochrane Central, EBSCO Cumulative Index to Nursing and Allied Health Literature, Institute of Electrical and Electronics Engineers and Institution of Engineering and Technology Xplore, Association for Computing Machinery Digital Library, China National Knowledge Infrastructure, Wanfang Data), preprint servers (arXiv, medRxiv, bioRxiv), and systematic review protocol registers (PROSPERO and Cochrane protocol via Cochrane Central). To identify additionally eligible systematic reviews, the reference lists of all included articles were screened, and radiomics experts were consulted.

We include all the systematic reviews with meta-analysis concerning on the radiomics applications for diagnostic, predictive, or prognostic purposes in humans. There was no restriction for publication period, target population, study setting, or comparator group, while only articles in English, Chinese, Japanese, German, and French were available. We excluded with following criteria: (a) primary study systematic review without meta-analysis, and article with insufficient information for assessment; (b) systematic review purely assessed artificial intelligence, machine learning or deep learning; (c) systematic review focused on methodology or robustness issue other than clinical-relevant questions. After excluding duplicates, we screened the titles and abstract for potentially available systematic reviews and then, confirmed their eligibility by reading the full-texts, supplementary materials, and related review protocols. The detailed search strategy and study selection process are provided in Additional file 1: Note S2.

### Data extraction and study assessment

The data were extracted according to a predefined data extraction sheet (Additional file 1: Table S1). This sheet includes bibliographical information, study characteristics, and effect metrics at level of meta-analyses and those at level of individual primary studies. The contingency tables at the level of individual studies were extracted or reconstructed for repeating meta-analysis.

The PRISMA (Preferred Reporting Items for Systematic reviews and Meta-Analyses) 2020 checklist [22], the AMSTAR-2 (A MeaSurement Tool to Assess systematic Reviews, version 2) tool [23], and the ROBIS (Risk Of Bias In Systematic reviews) tool [24] for reporting quality, methodological quality, and risk of bias assessment, respectively. The operational definitions of these three tools can be found in Additional file 1: Tables S2 to S5. The PRISMA 2020 checklist is updated to guide systematic reviewers for transparently reporting with a checklist for abstract of twelve items and a checklist for full-text of twenty-seven items. The AMSTAR-2 tool is developed and modified for critically appraising systematic reviews with sixteen questions to assess their methodological quality. The three-phase ROBIS tool is specifically designed to assess the risk of bias in systematic reviews covering four domains: study eligibility criteria, identification and selection of studies, data collection and study appraisal, and synthesis and findings. These tools have been tailored to radiological systematic reviews and applied for identifying overlooked reporting items, insufficient methodology, and potential risk of bias, respectively [25–27].

A training phase was introduced to test and modify the tools to reach an operational definition of each item and make sure that all reviewers have a shared understanding. Reached consensus during data extraction and quality assessments is available in Additional file 1: Note S3.

### Data analysis and strength of evidence

The statistical analysis was performed with R language version 4.1.3 within using relevant packages [28, 29]. The differences of PRISMA adherence rate, AMSTAR-2 rating, and ROBINS assessment were compared by (a) Journal Citation Report quartile (Q1 or Q2-Q4), (b) journal type (imaging or non-imaging), (c) first authorship (radiologist or non-radiologist), (d) biomarker (diagnostic, predictive, or prognostic), and (e) publication year (2020, 2021, or 2022), using student’s t test, one-way analysis of variance, and Chi-square test. A two-tailed *p* < 0.05 was recognized as statistical significance, unless specified otherwise.

The meta-analyses were re-performed with R language version 4.1.3 using relevant packages to allow evidence rating [30, 31]. The diagnostic odds ratio (OR) and the corresponding 95% confidence interval (CI) were pooled as summary effect size using random-effect models, and corresponding *p* values were calculated. The sensitivity, specificity, area under curve and index of concordance were not included for analysis, because the corresponding methodology has not been well established so far [21]. The *I*^2^ statistic was used to assess heterogeneity among primary studies. The 95% prediction intervals (PI) were calculated to facilitate more conservative prediction for potential application of radiomics models. The Egger’s test was conducted for small-study effects and publication bias. Excess significance bias was evaluated by a Chi-square test comparing the actual observed number of primary studies with a *p* < 0.05 with the expected number of primary studies with statistical significance.

The strength of evidence supporting radiomics for clinical use was categorized into five levels: convincing, highly suggestive, suggestive, weak, and not suggestive (Additional file 1: Table S6), based on the results of a series of aforementioned analyses [21]. The detailed data analysis process is available in Additional file 1: Note S4.

## Results

### Literature search

**Fig. 1:**
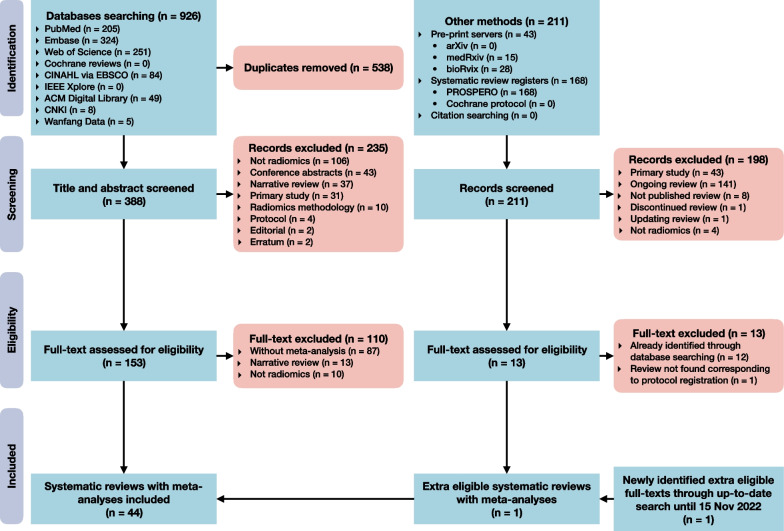
Flow diagram of systematic review search and selection

The flow diagram of selection process is shown in Fig. 1. Our primary literature search resulted in 926 records, in which 43 systematic reviews were included. No extra available systematic review was identified through preprint servers, systematic review protocol registers, or citation searching. The up-to-date search identified 1 extra eligible systematic review. Finally, 44 systematic reviews were included for the current overview [32–75]. The lists of the included systematic reviews with meta-analyses, and the excluded articles with justifications are provided in Additional file 1: Note S5.

### Study characteristics

The characteristics of the included systematic reviews were summarized (Table 1 and Additional file 1: Tables S7 and S8). The systematic reviews most frequently evaluated the application of radiomics in breast cancer (*n* = 7), followed by glioma (*n* = 5) and liver cancer (*n* = 4). The systematic reviews evaluating non-oncological diseases were less common. Only 2 and 1 systematic review investigated the radiomics in COVID-19 and pancreatitis, respectively.

**Table 1:** Characteristics of included systematic reviews

Characteristics	Data
Included primary studies, mean ± standard deviation, median (range)	22.4 ± 22.7, 15 (5 to 133)
Impact factor, mean ± standard deviation, median (range)	5.33 ± 1.63, 5.74 (2.37 to 10.06)
JCR quartile, *n*	*N* = 42*
Q1	18
Q2-Q4	24
Not applicable	2
Journal type, *n*	*N* = 44
Imaging	22
Non-imaging	22
First authorship, *n*	*N* = 44
Radiologist	21
Non-radiologist	23
Imaging modality, *n*	*N* = 81**
CT	25
MRI	35
PET	11
US	8
MMG	2
Biomarker, *n*	*N* = 44
Diagnostic	25
Predictive/Prognostic	13
Diagnostic and Predictive/Prognostic	6
Topics	*N* = 44
Oncologic	
Breast	7
Chest	4
Gastrointestinal	9
Genitourinary	6
Head and Neck	3
Musculoskeletal	3
Neuro	7
Gynecologic	2
Non-oncologic	
COVID-19	2
Pancreatitis	1
Quality assessment tool, *n*	*N* = 70**
CLAIM	1
IBSI	2
PROBAST	2
QUADAS-2	30
RQS	31
TRIPOD	4

*Two systematic reviews have no impact factor [35, 44]. ** Systematic reviews with multiple imaging modalities, or multiple quality assessment tools were counted, respectively. CLAIM = Checklist for Artificial Intelligence in Medical Imaging, IBSI = Image Biomarker Standardization Initiative, Journal Citation Report, PROBAST = Prediction Model Risk of Bias Assessment Tool, QUADAS = Quality Assessment of Diagnostic Accuracy Studies, RQS = Radiomics Quality Score rating, TRIPOD = Transparent Reporting of a Multivariable Prediction Model for Individual Prognosis or Diagnosis

The quality assessment tools used in included systematic reviews varied (Table 1). The Radiomics Quality Score (RQS) rating was the most employed tool (*n* = 31), followed by the Transparent Reporting of a multivariable prediction model for Individual Prognosis Or Diagnosis (TRIPOD) checklist (*n* = 4), the Image Biomarker Standardization Initiative (IBSI) checklist (*n* = 2), and the CheckList for Artificial Intelligence in Medical imaging (CLAIM) (*n* = 1). The revised QUality Assessment of Diagnostic Accuracy Studies (QUADAS-2) tool, and Prediction model Risk Of Bias ASsessment Tool (PROBAST) were applied by 30 and 2 systematic reviews for risk of bias assessment.

### Quality and risk of bias assessment

**Fig. 2:**
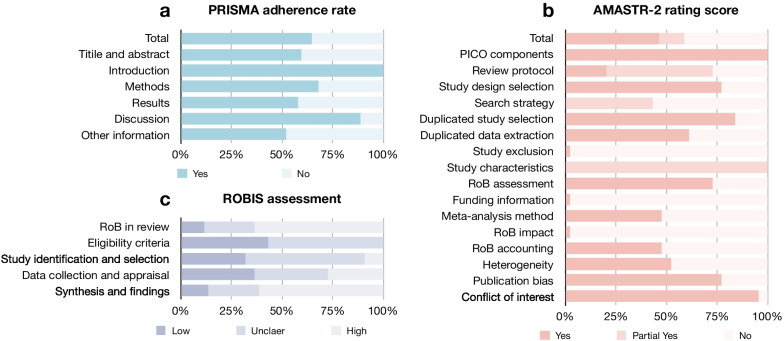
Result of quality assessment of included systematic reviews. **a** PRISMA adherence rate, **b** AMASTR-2 rating score, and **c** ROBIS assessment

The result of quality assessment is presented in Fig. 2 and Table 2. The evaluation results for individual systematic reviews are available in Additional file 1: Tables S9 to S11. The overall mean ± standard deviation (median, range) of PRISMA adherence rate was 65 ± 9% (64%, 48%-83%) for reporting quality. The AMSTAR-2 rated 5 and 39 systematic reviews as low and critically low confidence in methodological quality assessment, respectively. The overall risk of bias assessment by ROBIS tool resulted 5, 11, and 28 systematic reviews as having a low, unclear, and high risk of bias, respectively. The PRISMA adherence rate of systematic reviews with a first authorship of radiologist was higher than those without (68 ± 7 vs 62 ± 10, *p* = 0.023).

**Table 2:** Result of quality assessment of included systematic reviews

Subgroup	No. of systematic reviews	PRISMA adherence rate	AMSTAR-2 rating		ROBINS assessment		
		Mean ± standard deviation, %	Low, *n*	Critically low, *n*	Low, *n*	Unclear, *n*	High, *n*
Overall	44	65 ± 9	5	39	5	11	28
JCR quartile (*N* = 42), *p*-value		0.406		> 0.99			0.073
Q1	18	67 ± 8	2	16	0	4	14
Q2-Q4	24	64 ± 10	3	21	5	7	12
Journal type (*N* = 44), *p*-value		0.689		0.154			0.291
Imaging	22	64 ± 10	4	18	3	5	14
Non-imaging	22	65 ± 8	1	21	2	6	14
First authorship (*N* = 44), *p*-value		0.023		0.187			0.344
Radiologist	21	68 ± 7	1	20	3	5	13
Non-radiologist	23	62 ± 10	4	19	2	6	15
Biomarker (*N* = 44), *p*-value		0.124		0.117			0.132
Diagnostic	25	64 ± 9	5	20	4	9	12
Predictive/Prognostic	13	63 ± 9	0	13	1	2	10
Diagnostic and Predictive/Prognostic	6	72 ± 8	0	6	0	0	6
Publication year (*N* = 44), *p*-value		0.208		0.698			0.499
2020	3	65 ± 10	0	3	0	1	2
2021	20	62 ± 9	3	17	1	4	15
2022	21	67 ± 8	2	19	4	6	11

### Meta-analysis and strength of evidence

**Fig. 3:**
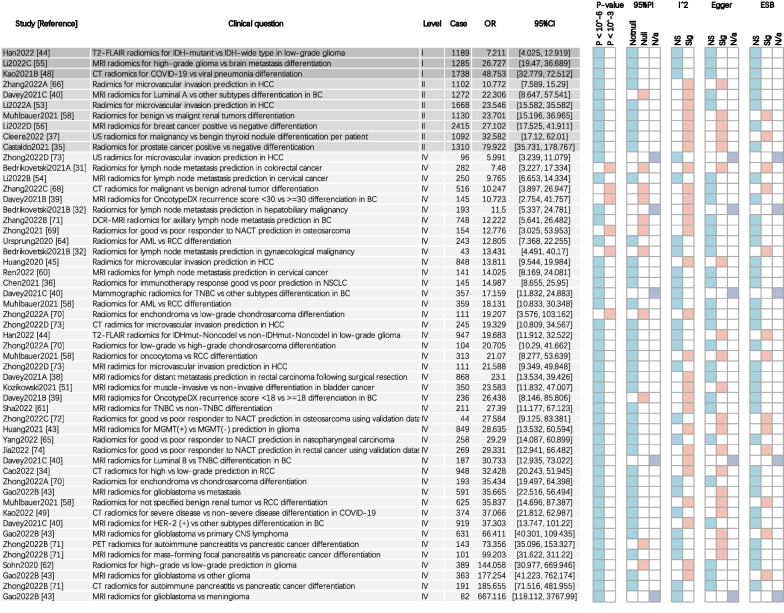
Summary of evidence rating. AML = angiomyolipoma, BC = breast cancer, HCC = hepatocellular carcinoma, NSCLC = non-small cell lung cancer, RCC = renal clear cell carcinoma, NACT = neoadjuvant chemotherapy, TNBC = triple negative breast cancer. NS = not significant, Sig = significant, N/a = not applicable

There were 53 meta-analyses in 38 systematic reviews re-conducted based on extracted or reconstructed data, covering 497 primary studies, 65,955 subjects, and 29,408 events [32, 33, 35–41, 44–47, 49–59, 61–63, 65–75] (Fig. 3). The meta-analyses in 6 systematic reviews were excluded due to unavailable data [34, 42, 43, 48, 60, 64]. Up to 47 meta-analyses reached a stringent *p*-value of less than 10^−6^, and 6 meta-analyses presented *p*-values < 10^−3^. None of the meta-analyses was deemed as non-significant. Twenty-eight meta-analyses presented an *I*^2^ > 50%. There were 5 meta-analyses conducting with less than three primary studies. For those performing with three or more primary studies, the 95%PI excluded the null value in 37 meta-analyses. Egger’s test of 28 meta-analyses reached *p* > 0.05 for indicating no small-study effects or publication bias. The excess significance bias was not presented in 35 meta-analyses.

Accordingly, there were 3, and 7 meta-analyses rated as convincing, and highly suggestive level of evidence, respectively (Table 3). The radiomics has been rated as convincing level of evidence in (1) T2-FLAIR radiomics for IDH-mutant vs IDH-wide type differentiation in low-grade glioma (diagnostic OR 7.2, 95%CI 4.0 to 12.9; *p* = 3.13 × 10^−11^), (2) CT radiomics for COVID-19 vs other viral pneumonia differentiation (OR 26.7, 95%CI 19.5 to 36.7; *p* = 4.54 × 10^−82^), and (3) MRI radiomics for high-grade glioma vs brain metastasis differentiation (OR 48.8, 95%CI 32.8 to 72.5; *p* = 7.25 × 10^−92^). The meta-analyses were rated as highly suggestive mainly due to high heterogeneity, significant small-study effects and publication bias. In spite of these dramatic statistical significances, 43 meta-analyses were rated as weak pieces of evidence. The reason for them failed to reach a higher level of evidence was mainly inadequate number of participants.

**Table 3:** Summary of convening and highly suggestive evidence

Study [References]	Clinical question	Evidence rating	PRISMA, %	AMSTAR	ROBINS
Han2022 [45]	T2-FLAIR radiomics for IDH-mutant vs IDH-wide type in low-grade glioma	Convincing	67	Critically low	Unclear
Kao2021B [56]	CT radiomics for COVID-19 vs viral pneumonia differentiation	Convincing	56	Critically low	High
Li2022C [49]	MRI radiomics for high-grade glioma vs brain metastasis differentiation	Convincing	67	Low	Unclear
Li2022A [54]	Radiomics in microvascular invasion prediction in hepatocellular carcinoma	Highly suggestive	77	Low	Unclear
Davey2021C [41]	MRI radiomics in Luminal A vs other subtypes differentiation in breast cancer	Highly suggestive	52	Critically low	High
Zhang2022A [67]	Radiomics in microvascular invasion prediction in hepatocellular carcinoma	Highly suggestive	77	Low	Low
Li2022D [57]	MRI radiomics in breast cancer positive vs negative differentiation	Highly suggestive	58	Critically low	High
Castaldo2021 [36]	Radiomics in prostate cancer positive vs negative differentiation	Highly suggestive	67	Critically low	Unclear
Mühlbauer2021 [59]	Radiomics in benign vs malignant renal tumors differentiation	Highly suggestive	60	Critically low	High
Cleere2022 [38]	US radiomics in malignancy vs benign thyroid nodule per patient	Highly suggestive	52	Critically low	High

## Discussion

An increasing number of studies are investigating the potential of radiomics as a diagnostic, predictive, or prognostic tool in multiple clinical scenarios, while none of the radiomics academic research has been successfully translated into daily clinical practice. Our overview of systematic reviews with meta-analyses identified 44 systematic reviews and reperformed 53 meta-analyses. The radiomics seemed to be convincing tools in answering three clinical questions including: (1) differentiation of IDH-mutant vs IDH-wide type in low-grade glioma, (2) differentiation of COVID-19 vs other viral pneumonia, and (3) differentiation of high-grade glioma vs brain metastasis. However, the included systematic reviews were insufficient in reporting, suboptimal in methodological quality, and with high risk of bias. The suboptimal study quality might lead to insufficient confidence in radiomics application and thereby hinder the clinical translation of radiomics even there was high-level of supporting evidence.

The radiomics were most frequently employed in oncological field with a representing example of breast cancer which accounting for seven of included systematic reviews, resulting only three non-oncological radiomics systematic reviews. Sollini et al. [16] declared that the number of oncological image minding studies was six-times of those in non-oncological field. Spadarella et al. [17] found that more than nine tenths of their included systematic reviews focused on oncological radiomics. It is not surprising because the concept of radiomics was raised to mine the medical images for extra deeper information related to oncological genomics [1]. However, the radiomics-biological correlation is more than radio-genomics but covers the diverse clinical, imaging, and molecular profile data, which allow understanding of complex diseases to achieve accurate diagnosis in order to provide the best possible treatment [12, 76]. The radiomics investigations are encouraged to expand to the non-oncological field for wider potential applications.

The quality and risk of bias assessment tools for radiomics systematic reviews varied. The RQS and QUADAS-2 tool were the most used tool for study quality and risk of bias assessment, respectively. The RQS was most used for quality assessment in the included systematic reviews and has been long served a necessary role as the de facto reference tool for assessing radiomics studies [17]. However, the RQS was far from perfect. With an increasing trend of deep learning application in radiomics, RQS could not well identify the advantages and disadvantages in radiomics studies applying as the CLAIM [72]. The TRIPOD checklist might further identify room for improvement in radiomics studies, but some items were not suitable for radiomics studies [76]. The IBSI checklist has highly overlapped with other checklists and somehow too complicated to use [73]. Recently, CheckList for EvaluAtion of Radiomics research (CLEAR) has been developed as a single documentation standard for radiomics research that can guide authors and reviewers [77. However, the reproducibility and effectiveness of this tool has not been fully investigated yet. The QUADAS-2 tool was employed repeatedly because most of the radiomics studies were diagnostic accuracy studies. The PROBAST tool might be also suitable for most of radiomics studies because it is developed for predictive models of both diagnostic and prognostic purpose. Other guidelines and checklists are developed or under development for radiomics and artificial intelligence studies including artificial intelligence extensions for TRIPOD, QUADAS-2, and PROBAST [79]. Further validation is needed for their feasibility and efficiency in improving quality of radiomics studies.

The evidence rating highlighted three pieces of convincing evidence of radiomics approaches answering clinical questions. However, there were seven pieces of highly suggestive evidence hindered by the high heterogeneity. We did not investigate the potential source of heterogeneity due to the workload, but this should be explored in the individual systematic review to allow interruption of the results. Unfortunately, these systematic reviews did not perform related investigations. Indeed, less than a half of included systematic reviews conducted such an analysis. Another reason for failing to reach convincing level of evidence is significant small-study effects and publication bias. This was assessed by more than four fifths of the included systematic reviews. The radiomics were not rated as sufficient tools for other clinical applications. There were more pieces of weak evidence due to insufficient participants. This could not be solved by systematic reviews, but it might be overcome with more carefully designed prospective, multicenter, randomized controlled trials and data sharing [9, 11, 12]. Another concern on the systematic reviews and meta-analyses of radiomics was their relatively low study quality. Although our overview identified three potential application of radiomics with high-level of supporting evidence, they were all with suboptimal quality that should be taken into consideration when applying the evidence. A systematic approach is encouraged to establish to comprehensively evaluate the radiomics tool, in order to tell whether the tool can be used in the clinical practice. The GRADE (grading of recommendations assessment, development and evaluation) system can be used for diagnostic tests or strategies [80], but the feasibility of this approach for radiomics researches needs to be verified.

There are several limitations to address. First, we only included systematic reviews with meta-analyses to identify the most possible candidate to be supported by high-level evidence. We only included the primary studies mentioned in the meta-analyses for re-analysis, because updating the literature search may lead to a too heavy workload. As a rapidly developing field, our meta-analyses may not include all the eligible radiomics studies. Second, most of the meta-analyses were based on training or validation dataset, which potentially overestimated the results. The future analysis is encouraged to be conducted using testing dataset of the strictly designed studies. Third, the majority of the included primary studies were retrospective, single-center, small-scale studies and have been assessed as suboptimal quality. Further, the overall quality of included systematic reviews was also insufficient. Therefore, the aforementioned evidence level rating results should be cautiously interpreted. Lastly, the evidence rating criteria of diagnostic accuracy tests have not been well established. We only estimated the diagnostic odds ratio as effect size, but not the corresponding sensitivity, specificity, and area under curve value for each meta-analysis, whose potential role in evidence rating needs further investigation.

## Conclusion

In conclusion, our overview of systematic reviews and meta-analyses highlighted three convincing and seven highly suggestive level of evidence for radiomics in answering clinical questions, while the low study quality and high risk of bias might lead to insufficient confidence in clinical translation. Future research should provide more scientific base for those with low-level of evidence and seek to validate the radiomics algorithms in clinical settings for those with high-level of evidence. Systematic reviews and meta-analyses on radiomics researches could continuously help the stakeholder to identify knowledge gaps, biases, and priorities for future research to promote the radiomics translation from an academic tool for generating papers to a practicable adjunct toward clinical deployment.

## Supplementary Information

**Additional file 1:** Supplementary Materials, Supplementary Review Protocol, and Supplementary PRISMA Checklists.** Supplementary Note S1.** Review protocol.** Supplementary Note S2.** Study search strategy and study selection.** Supplementary Note S3.** Consensus reached during data extraction and quality assessment.** Supplementary Note S4.** Data synthesis and analysis methods.**Supplementary Note S5.** List of included full-texts and excluded full-texts with justifications. **Supplementary Table S1.** Data extraction sheet.** Supplementary Table S2.** PRISMA 2020 abstract checklist for reporting quality assessment.** Supplementary Table S3.** PRISMA 2020 checklist for reporting quality assessment.** Supplementary Table S4.** AMSTAR-2 tool for methodological quality assessment.** Supplementary Table S5.** ROBIS tool for risk of bias assessment.** Supplementary Table S6** Category of five levels of evidence based on meta-analyzes.** Supplementary Table S7.** Bibliographic information of included systematic reviews.** Supplementary Table S8.** Review topic of included systematic reviews.** Supplementary Table S9.** PRISMA adherence rate of included systematic reviews.** Supplementary Table S10.** AMSTAR-2 ratings of included systematic reviews.** Supplementary Table S11.** ROBIS tool assessments of included systematic reviews.

## Data Availability

All data generated or analyzed during this study are included in this published article and its Additional files.

## Abbreviations

AMSTAR-2: A MeaSurement Tool to Assess systematic Reviews, version 2
CLAIM: CheckList for Artificial Intelligence in Medical imaging
IBSI: Image Biomarker Standardization Initiative
PRISMA: Preferred Reporting Items for Systematic reviews and Meta-Analyses
PROBAST: Prediction model Risk Of Bias ASsessment Tool
QUADAS-2: Revised QUality Assessment of Diagnostic Accuracy Studies
ROBIS: Risk Of Bias In Systematic reviews
RQS: Radiomics Quality Score
TRIPOD: Transparent Reporting of a multivariable prediction model for Individual Prognosis Or Diagnosis
