# Supplementary material for: An overview of meta-analyses on radiomics: more evidence is needed to support clinical translation
Source: Insights Imaging. 2023 Jun 19;14:111. doi: 10.1186/s13244-023-01437-2 (PMC10279606; doi:10.1186/s13244-023-01437-2)
Supplement: Supplementary file 1 — Additional file 1: Supplementary Materials, Supplementary Review Protocol, and Supplementary PRISMA Checklists. Supplementary Note S1. Review protocol. Supplementary Note S2. Study search strategy and study selection. Supplementary Note S3. Consensus reached during data extraction and quality assessment. Supplementary Note S4. Data synthesis and analysis methods.Supplementary Note S5. List of included full-texts and excluded full-texts with justifications. Supplementary Table S1. Data extraction sheet. Supplementary Table S2. PRISMA 2020 abstract checklist for reporting quality assessment. Supplementary Table S3. PRISMA 2020 checklist for reporting quality assessment. Supplementary Table S4. AMSTAR-2 tool for methodological quality assessment. Supplementary Table S5. ROBIS tool for risk of bias assessment. Supplementary Table S6 Category of five levels of evidence based on meta-analyzes. Supplementary Table S7. Bibliographic information of included systematic reviews. Supplementary Table S8. Review topic of included systematic reviews. Supplementary Table S9. PRISMA adherence rate of included systematic reviews. Supplementary Table S10. AMSTAR-2 ratings of included systematic reviews. Supplementary Table S11. ROBIS tool assessments of included systematic reviews. [file 13244_2023_1437_MOESM1_ESM.pdf]

An overview of meta-analyses on radiomics: more evidence is needed to support clinical translation

Supplementary Materials

List of Supplementary Materials

Supplementary Note S1 Review protocol
Supplementary Note S2 Study search strategy and study selection
Supplementary Note S3 Consensus reached during data extraction and quality assessment
Supplementary Note S4 Data synthesis and analysis methods
Supplementary Note S5 List of included full-texts and excluded full-texts with justifications

Supplementary Table S1 Data extraction sheet
Supplementary Table S2 PRISMA 2020 abstract checklist for reporting quality assessment
Supplementary Table S3 PRISMA 2020 checklist for reporting quality assessment
Supplementary Table S4 AMSTAR-2 tool for methodological quality assessment
Supplementary Table S5 ROBIS tool for risk of bias assessment
Supplementary Table S6 Category of five levels of evidence based on meta-analyzes
Supplementary Table S7 Bibliographic information of included systematic reviews
Supplementary Table S8 Review topic of included systematic reviews
Supplementary Table S9 PRISMA adherence rate of included systematic reviews
Supplementary Table S10 AMSTAR-2 ratings of included systematic reviews
Supplementary Table S11 ROBIS tool assessments of included systematic reviews

Supplementary Note S1 Review protocol

PROSPERO registration

ROSPERO ID: CRD42021272746

Submit to PROSPERO: 10 Aug 2021

Last edited: 15 Nov 2022

Review question

What is the existing systematic review evidence for (1) the diagnostic, prognostic, and predictive accuracy of radiomics studies in clinical settings, and (2) quality assessment and risk of bias tools for these studies?

Searches

We will search the following databases for eligible systematic reviews: (1) Cochrane Database of Systematic Reviews (CDSR), (2) PubMed, (3) EMBASE, and (4) Web of Science. Literature search strategies will be developed using medical subject headings (MeSH) and derived words, including "radiomics", "systematic review", and "meta-analysis". Only systematic reviews published after Jan 01 2012 will be included, since the term "radiomics" was first coined in early 2012. The search strategy will include only terms relating to the review question. Publications must be available in English, Chinese, Japanese, German or French. To ensure literature saturation, we will check the reference lists of included systematic reviews identified through the search.

[Modification]

To better identify relevant studies, we decided to search more peer-reviewed databases, and gray literature resources. We will search the peer-reviewed electronic databases (PubMed, Embase, Web of Science, Cochrane reviews via Cochrane Central, EBSCO Cumulative Index to Nursing and Allied Health Literature, Institute of Electrical and Electronics Engineers and Institution of Engineering and Technology Xplore, Association for Computing Machinery Digital Library, China National Knowledge Infrastructure, Wanfang Data), preprint servers (arXiv, medRxiv, bioRxiv), and systematic review protocol registers (PROSPERO and Cochrane protocol via Cochrane Central). The term "radiomics" was firstly risen on 2012, but the similar method like histogram or texture analysis has been applied for image analysis before the term "radiomics" appeared. Therefore, we will give up the restriction for publication period. The publications must be available in English, Chinese, Japanese, German or French. To ensure literature saturation, we will check the reference lists of included systematic reviews identified through the search. The experts' opinion will also serve as a source for potentially eligible systematic reviews. An up-to-date search will be performed if it is considered to be necessary after the discussion within the review group.

Condition or domain being studied

Since Lambin et al first coined the term radiomics in early 2012, almost a decade has passed. Thousands of papers have been published in this rapidly evolving field. However, what is the existing systematic review evidence for (1) the diagnostic, prognostic, and predictive accuracy of radiomics studies in clinical settings, and (2) quality assessment and risk of bias tools for these studies, is unknown. Therefore, we aim to summarize current evidence from radiomics studies, and to help improve the quality of such studies.

Participants/population

Patients or participants assessed by radiomics approaches for clinical questions, typically diagnostic, prognostic, and predictive accuracy tests.

Intervention(s), exposure(s)

Diagnostic test accuracy of radiomics approaches used for clinical questions.

Comparator(s)/control

Not applicable.

Types of study to be included

Systematic reviews and meta-analyzes of diagnostic test accuracy. Systematic reviews concerning diagnostic test accuracy of radiomics approaches will be included. Meta-analyzes focusing on a specific clinical question will be included.

[Modification]

We will only include systematic reviews with meta-analyses to allow level of evidence rating.

Main outcome(s)

The bibliographic information, review questions, and quality and risk of bias assessment tools used in systematic reviews will be summarized. The rating of AMSTAR-2 tool, PRISMA 2020 checklist, and ROBIS tool per systematic review will be used as metrics of methodological quality, reporting quality, and risk of bias, respectively. If meta-analyzes concerning a specific clinical question were performed in included systematics reviews, statistical analyses for the evidence rating will conducted for levels of supporting evidence of meta-analyzes.

Measures of effect

Levels of supporting evidence of will be categorized into five levels: convincing, highly suggestive, suggestive, weak, and not suggestive based on the criteria shown as follows: $p < 10^{-6}$, > 1000 events, the largest study reaches statistical significance ($p < 0.05$), $I^2 < 50\%$, the null value excluded by the 95% PI, no small-study effects ($p > 0.1$ for Egger's test) and excess significance ($p > 0.1$), and survived the 10% credibility ceiling ($p < 0.05$) for strong evidence; $p < 10^{-6}$, > 1000 events, the largest study reaches statistical significance ($p < 0.05$) for highly suggestive evidence; $p < 10^{-3}$, > 1000 events for suggestive evidence; $p < 0.05$ for weak evidence; and $p > 0.05$ for not suggestive evidence.

[Modification]

Levels of supporting evidence of will be categorized into five levels: convincing, highly suggestive, suggestive, weak, and not suggestive based on the criteria shown as follows: convincing (class I) when number of cases>1000, $p<10^{-6}$, $I^2<50\%$, 95% prediction interval excluding the null, no small-study effects and no excess significance bias; highly suggestive (class II) when number of cases>1000, $p<10^{-6}$, largest study with a statistically significant effect and class I criteria not met; suggestive (class III) when number of cases>1000, $p<10^{-3}$ and class I–II criteria not met; weak (class IV) when $p<0.05$ and class I–III criteria not met; non-significant when $p>0.05$.

Data extraction (selection and coding)

All data will be managed using EndNote software. The first 15 records of titles and abstracts will be reviewed by two reviewers according to the inclusion and exclusion criteria as a pilot screening. Subsequent discussion will inform the screening notes. Title/abstract screening and full text screening will be conducted by the same two reviewers. A third reviewer will resolve any disagreements.

A data extraction tool will be established and then trialed on two randomly chosen studies, which fulfilled all the inclusion criteria. These shall be used to train reviewers to appropriately apply the data extraction tool. Data will be abstracted by one reviewer and double-checked by a second, with a third reviewer providing moderation as required. Key data extracted will include characteristics of included systematic reviews: (1) bibliographic information, (2) review questions, (3) quality and risk of bias assessment tools used, and (4) metrics of meta-analyzes.

Risk of bias (quality) assessment

AMSTAR-2 tool, PRISMA 2020 checklist, and ROBIS tool will be used for methodological quality, reporting quality, and risk of bias, respectively.

Strategy for data synthesis

A narrative synthesis will be provided with information presented in the text and/or tables to summarize and explain the characteristics and findings of the included studies. A quantitative synthesis will be done if the included studies are sufficiently homogenous. Additional analyses may be possible for comparing across included reviews, in which case we will consult with a statistical specialist regarding the validity and suitability of further analyses. All analysis will be based on aggregate data.

Analysis of subgroups or subsets

Not planned.

Type and method of review

Diagnostic, Prognostic, Narrative synthesis, Systematic review, Meta-analysis, Review of reviews

Keywords

Radiomics, Overview of Reviews

Conflicts of interest

The authors declare that they have no competing interests.

Supplementary Note S2 Study search strategy and study selection

Study search strategy

We firstly performed a preliminary search to confirm the availability of the search string. One of the reviewers (J. Z.) has experience in developing the search strings. The reviewer developed the search stirrings and validated their feasibility. The search string was developed by combining the variations of the terms of “radiomics”, “systematic review” and “meta-analysis”. To further identify relevant articles, the article type of “systematic review” or “meta-analysis” was used when it is available. Then, we conducted the formal search to identify potential available articles. The literature search was duplicated by two independent reviewers (J.Z. and either Y.H., Y.X., X.G., or D.D.). The disagreements were resolved by consults with a third independent reviewer (G.Z., S.M., H.C., Q.Y., G.Y., H.Z. or W.Y.). An up-to-date search was performed by two independent reviewers (J.Z. and either Y.H.).

1. Peer-reviewed databases

1.1 Cochrane review via Cochrane Library

Available via https://www.cochranelibrary.com

Preliminary search date: 15 Jul 2022

Articles retrieved: 434 records (0 Cochrane reviews, 0 Cochrane protocol, 434 clinical trials)

Formal search date: 30 Sep 2022

Articles retrieved: 462 records (0 Cochrane reviews, 0 Cochrane protocol, 462 clinical trials)

Search string:

radiomics OR radiogenomics OR radiomic OR radiogenomic

1.2 PubMed

Available via https://pubmed.ncbi.nlm.nih.gov

Preliminary search date: 15 Jul 2022

Articles retrieved: 179

Formal search date: 30 Sep 2022

Articles retrieved: 205

Search string:

(radiomic* OR radiogenomic*) AND (("systematic review" OR "systematic reviews as Topic"[Mesh] OR "Systematic Review"[Publication Type]) OR ("meta-analysis" OR "meta-analyses" OR "meta-analyzes" OR "Meta-Analysis as Topic"[Mesh] OR "Meta-Analysis"[Publication Type]))

1.3 Embase

Available via www.embase.com

Preliminary search date: 15 Jul 2022

Articles retrieved: 289

Formal search date: 30 Sep 2022

Articles retrieved: 324

Search string:

(("radiomic":ti,ab,kw OR "radiomics":ti,ab,kw OR "radiomics"/exp OR "radiomic"/exp) OR ("radiogenomic":ti,ab,kw OR "radiogenomics":ti,ab,kw OR "radiogenomic"/exp OR "radiogenomics"/exp)) AND (("systematic review":ti,ab,kw OR "systematic review"/exp) OR ("meta-analysis":ti,ab,kw OR "meta analysis"/exp))

1.4 Web of Science

Available via apps.webofknowledge.com

Preliminary search date: 15 Jul 2022

Articles retrieved: 228

Formal search date: 30 Sep 2022

Articles retrieved: 251

Search string:

(TS=(radiomic) OR TS=(radiogenomic) OR TS=(radiomics) OR TS=(radiogenomics)) AND (TS=(systematic review) OR TS=(systematic reviews)OR TS=(meta-analysis) OR TS=(meta-analyses) OR TS=(meta-analyzes))

1.5 EBSCO Cumulative Index to Nursing and Allied Health Literature

Available via https://search.ebscohost.com

Preliminary search date: 15 Jul 2022

Articles retrieved: 74

Formal search date: 30 Sep 2022

Articles retrieved: 84

Search string:

(radiomics OR radiogenomics OR radiomic OR radiogenomic) AND ("systematic review" OR "systematic reviews" OR "meta-analysis" OR "meta-analyses" OR "meta-analyzes")

1.6 Institute of Electrical and Electronics Engineers and Institution of Engineering and Technology Xplore

Available via https://ieeexplore.ieee.org/Xplore/home.jsp

Preliminary search date: 15 Jul 2022

Articles retrieved: 0

Formal search date: 15 Sep 2022

Articles retrieved: 0

Search string:

("All Metadata":radiomics OR "All Metadata":radiogenomics OR "All Metadata":radiomic OR "All Metadata":radiogenomic) AND ("All Metadata":"systematic review" OR "All Metadata":"meta-analysis" OR "All Metadata":"meta-analyses" OR "All Metadata":"meta-analyzes")

1.7 Association for Computing Machinery Digital Library

Available via https://dl.acm.org

Preliminary search date: 15 Jul 2022

Articles retrieved: 46

Formal search date: 30 Sep 2022

Articles retrieved: 49 (33 meeting abstracts, 16 journal articles)

Search string:

[[All: radiomics] OR [All: radiogenomics] OR [All: radiomic] OR [All: radiogenomic]] AND [[All: "systematic review"] OR [All: "systematic reviews"] OR [All: "meta-analysis"] OR [All: "meta-analyses"] OR [All: "meta-analyzes"]]

1.8 China National Knowledge Infrastructure

Available via http://www.cnki.net

Preliminary search date: 15 Jul 2022

Articles retrieved: 7

Formal search date: 30 Sep 2022

Articles retrieved: 8

Search string:

(TKA="影像组学" OR TKA="影像基因组学") AND (TKA="系统综述" OR TKA="荟萃分析")

English translation:

(radiomics OR radiogenomics) AND (systematic review OR meta-anlysis)

1.9 Wanfang Data

Available via https://www.wanfangdata.com.cn

Preliminary search date: 15 Jul 2022

Articles retrieved: 4

Formal search date: 30 Sep 2022

Articles retrieved: 5

Search string:

(主题:("影像组学") OR 主题:("影像基因组学")) AND (主题:("系统综述") OR 主题:("荟萃分析"))

English translation:

(radiomics OR radiogenomics) AND (systematic review OR meta-anlysis)

2. Preprint servers

2.1 medRxiv

Available via https://www.medrxiv.org

Preliminary search date: 15 Jul 2022

Articles retrieved: 14

Formal search date: 30 Sep 2022

Articles retrieved: 15

Search string:

(radiomics OR radiogenomics OR radiomic OR radiogenomic) AND ("systematic review" OR "meta-analysis")

2.2 bioRxiv

Available via https://www.biorxiv.org

Preliminary search date: 15 Jul 2022

Articles retrieved: 26

Formal search date: 30 Sep 2022

Articles retrieved: 28

Search string:

(radiomics OR radiogenomics OR radiomic OR radiogenomic) AND ("systematic review" OR "meta-analysis")

2.3 arXiv

Available via https://arxiv.org

Preliminary search date: 15 Jul 2022

Articles retrieved: 0

Formal search date: 30 Sep 2022

Articles retrieved: 0

Search string:

(radiomics OR radiogenomics OR radiomic OR radiogenomic) AND ("systematic review" OR "meta-analysis")

3. Systematic review registers

3.1 PROSPERO register

Available via https://www.crd.york.ac.uk/PROSPERO/

Preliminary search date: 15 Jul 2022

Articles retrieved: 152 records (12 review completed published, 1 review completed published being updated)

Formal search date: 30 Sep 2022

Articles retrieved: 168 records (17 review completed published, 8 review completed not published, 1 review completed published being updated, 1 review discontinued, 141 review ongoing)

Search string:

radiomics OR radiogenomics OR radiomic OR radiogenomic

3.2 Cochrane protocol via Cochrane Library

Available via https://www.cochranelibrary.com

Preliminary search date: 15 Jul 2022

Articles retrieved: 434 records (0 Cochrane review, 0 Cochrane protocol, 434 clinical trails)

Formal search date: 30 Sep 2022

Articles retrieved: 462 records (0 Cochrane reviews, 0 Cochrane protocol, 462 clinical trials)

Search string:

radiomics OR radiogenomics OR radiomic OR radiogenomic

Study selection

All data were managed using EndNote software version X9. The first 15 records were reviewed by two reviewers according to the inclusion and exclusion criteria as a pilot screening. Subsequent discussion informed the screening notes. Title and abstract screening and full text screening were duplicated by two independent reviewers (J.Z. and either Y.H., Y.X., X.G., or D.D.). The disagreements were resolved by consults with a third independent reviewer (G.Z., S.M., H.C., Q.Y., G.Y., H.Z. or W.Y.). The newly identified potentially eligible studies via the up-to-date search were reviewed for consideration for inclusion by two independent reviewers (J.Z. and Y.H.).

1. Studies included in narrative synthesis

1.1 Inclusion Criteria: (1) systematic reviews of diagnostic, prognostic, and predictive accuracy tests with meta-analyses; (2) patients or participants assessed by radiomics approaches for clinical questions; (3) studies are reported in English, Japanese, Chinese, German or French; (4) institutional full-text availability.

1.2 Exclusion Criteria: (1) duplicate studies; (2) systematic reviews of robustness, reproducibility, or repeatability of radiomics features or topics other than clinical questions; (3) primary studies, technical reports, letters to editors, comments to published studies, conference proceedings, case reports, brief communications and articles with insufficient information for assessing quality; (4) not human studies; (5) not radiomics studies.

2. Studies included in meta analyzes

2.1 Inclusion criteria: (1) systematic reviews and meta-analyses with a sufficient number of studies attempts to answer a similar question; (2) studies sufficient data to extract the two-by-two tables, or reconstructed with documented sensitivity (Se), specificity (Sp), accuracy, positive predictive value (PPV), negative predictive value (NPV) and likelihood ratio (LR), diagnostic odds ratio (DOR), and their corresponding 95% confidence interval (CI), or with those could be calculated using published data; (3) the latest and largest among the systematic reviews and meta-analyzes on the same clinical question.

2.2 Exclusion criteria: (1) systematic reviews that do not contain quantitative synthesis; (2) insufficient data, and unavailable to be calculated using published data; (3) older or smaller systematic reviews and meta-analyzes on the same clinical question.

3. Overlapping primary studies

We compared the meta-analysis attempting to answer similar clinical questions. However, none of the later or larger meta-analysis covered all the primary studies in the older or former meta-analysis, because the clinical question of these meta-analyzes were subtly different to each other. We did not merge them for a larger scale of meta-analysis.

Supplementary Note S3 Consensus reached during data extraction and quality assessment

1. Data extraction

We developed a data extraction sheet to collect study data. As the reviewers have different levels of experience and knowledge, the items listed were reviewed and discussed to ensure that all reviewers had clear knowledge of the procedures. A training phase was introduced before the formal extraction. The data extraction was duplicated by two independent reviewers (J.Z. and either Y.H., Y.X., X.G., or D.D.). The disagreements were resolved by consults with a third independent reviewer (G.Z., S.M., H.C., Q.Y., G.Y., H.Z. or W.Y.).

During the training phase, two randomly chosen articles from all articles fulfilled the inclusion criteria for discussion were used to train reviewers. They thoroughly read the two randomly chosen articles including the supplementary materials, and measured each study independently. A structured data collection tool was modified and used to help them reach agreement. Disagreements were discussed in order to achieve a shared understanding of each parameter. This pre-defined and piloted data extraction tool was used in the formal data extraction phase. The details of the data extraction tool can be found in Supplementary Table S1.

The following items were discussed to reach consensus:

(1) **The first authorship of the study:** The first authorship of the study was decided by the department of the first author. The author was considered as a radiologist if he/she was in the department of radiology, imaging, nuclear medicine, etc.; or the author was considered as a non-radiologist.

(2) **Impact factor, Journal Citation Reports (JCR) quartile, and Journal type**: Impact factor, JCR quartile, and Journal type are extracted according to Journal Citation Reports 2021, which became available on Jun 2022 (https://access.clarivate.com/). The Journal type was decided according to the Journal Citation Report. If the journal is in the radiology, imaging, nuclear medicine type, it was considered as an imaging journal; or the journal was considered as non-imaging journal. For journal has multiple typing, they were considered as imaging journals if one of the typing is imaging.

(3) **Publication year**: The publication year was decided according to the formal publication year, if the article was formally published (not early access, first online, or something like that); or decided according to the year the article firstly available online, if the article has not been formally published, but currently available as early access, first online, or something like that.

(4) **Quality assessment tools & risk of bias tools:** The systematic reviews in radiomics may employ multiple tools for risk of bias or quality assessment, including QUADAS-2, PROBAST, RQS, TRIPOD, CLAIM, and IBSI. The risk of bias tool was defined as QUADAS-2 and PROBAST, which are designed for diagnostic, predictive, or prognosis modeling studies. The RQS and IBSI were considered as tools for methodology quality, the TRIPOD for reporting transparency, and the CLAIM for both methodology quality and reporting transparency.

2. Quality assessment

We employed PRISMA (Preferred Reporting Items for Systematic reviews and Meta-Analyses) 2020 checklist, AMSTAR-2 (A MeaSurement Tool to Assess systematic Reviews, version 2) tool, and ROBIS (Risk Of Bias In Systematic reviews) tool for methodological quality, reporting quality, and risk of bias assessment, respectively. The details of these three tools can be found in Supplementary Tables S2 to S5. The quality assessment was duplicated by two independent reviewers (J.Z. and either Y.H., Y.X., X.G., or D.D.) The disagreements were resolved by consults with a third independent reviewer (G.Z., S.M., H.C., Q.Y., G.Y., H.Z. or W.Y.).

During the training phase, two randomly chosen articles from all articles fulfilled the inclusion criteria for discussion were used to train reviewers. They thoroughly read the two randomly chosen articles including the supplementary materials, and discussed the items in AMSTAR-2 tool and PRISMA 2020 checklist to reach agreement. Signal questions in ROBIS tool were modified to our review. Disagreements were discussed in order to achieve a shared understanding of each item. This pre-defined and piloted data extraction tool was used in the formal data extraction phase. During the quality assessment, all the included studies were rated by two reviewers together. Any disagreement was resolved by discussion to reach a consensus or consultation with a third reviewer.

2.1 PRISMA 2020 checklist for reporting quality

The PRISMA checklist was designed to help systematic reviewers transparently report why the review was done, what the authors did, and what they found. The PRISMA 2020 statement replaces the 2009 statement and includes new reporting guidance that reflects advances in methods to identify, select, appraise, and synthesize studies. The structure and presentation of the items have been modified to facilitate implementation. The PRISMA 2020 checklist has been updated to guide systematic reviewers for transparently reporting with twenty-seven items according to six sections.

The following items were discussed to reach consensus:

(1) **PRISMA #2.3 (Eligibility criteria in Abstract):** In most cases, the inclusion and exclusion criteria were too long to put in the abstract section of systematic review. However, we did not refer this item to the inclusion and exclusion in the Method section, because this refers to PRISMA #5. We consider the inclusion criteria is provided when there was a simple description of study characteristics with both term “inclusion/include” and “exclusion/exclude”.

(2) **PRISMA #2.7 (Included studies in Abstract) and #2.8 (synthesis of results in Abstract):** For the PRISMA #2.7, both the number of study and participants are necessary; while for the PRISMA #2.8, the number of study and participants are necessary were not necessary.

(3) **PRISMA #2.11 (Funding in Abstract) and #25 (Support):** PRISMA #2.11 refers to a section in the Abstract section, the funding information at the end of the article is not recognized. PRISMA #25 refers to not only financial but also non-financial support for the review, as well as their role in the review. Only the funder and funding number are not enough for this item. A declaration of no funding is considered as fulfilled.

(4) **PRISMA #2.12 (Registration in Abstract) and PRISMA #24 (Registration and protocol):** PRISMA #2.12 only need the name and number of registrations to fulfill, while PRISMA #24 needs more detailed information, including the name and number of registrations (PRISMA #24a), protocol information (PRISMA #24b), and amendments (PRISMA #24c). However, not all these items are described in the text. We consider the PRIMSA #24a, #24b, and #24c are fulfilled when a link to a published protocol or a registration (with number) on PROSPERO or Cochrane Library is indicated. These registers are the most widely accepted systematic review protocol registers. These items are considered also as fulfilled if a declaration of no protocol is provided.

(5) **PRISMA #7 (Search strategy):** This item is considered to be fulfilled when the detailed search string for each database is provided. Only one sentence describing the keyword applied or an example of search string in a specific database (such as PubMed) is not enough for this item. We decided to use this relatively strict criteria, because the search strategy can be easily provided as supplementary material.

(6) **PRISMA #8 (Selection process), #9 (Data collection process) and #11 (Risk of bias assessment):** For these three items, the tools are usually described in the text, while how reviewers worked, and whether the reviewers worked independent, are usually not described. We considered these items as fulfilled when the tool, number of

reviewers, and the independence were described. For the PRISMA #11, risk of bias can be assessed by typical risk of bias assessment tools, study methodological or reporting quality assessment tools.

(7) **PRISMA #13a (Synthesis methods – synthesis grouping):** We considered the key point of this item is description of the process or the criteria for selecting studies for data synthesis, but which studies are selected for synthesis. Therefore, we recognize systematic reviews with description of the process or the criteria as fulfilled for this item, whether the specific studies are described or not.

(8) **PRISMA #13c (Synthesis methods – display results):** A simple sentence is enough, such as "the data were descriptively summarized by text or table", or some type of plots "were used for visually display the results".

(9) **PRISMA #13d (Synthesis methods – methods and selection rationale):** Most of the studies provide the methods used for data synthesis, but usually without the reason for choosing the specific methodology. We consider this item as fulfilled only when there is a reason provided, or some description of which methodology would be selected on which situation.

(10) **PRISMA #13e (Synthesis methods – explore cause of heterogeneity) and #13f (Synthesis methods – sensitivity analysis):** The following methodology is considered as those exploring cause of heterogeneity (PRISMA #13e), including subgroup analysis, meta-regression, and threshold effect assessment. Although the subgroup analysis can be considered as one of the methodologies of sensitivity analysis (PRISMA #13f), we only consider it is used for sensitivity analysis when it is declared in the text. Other methodologies for sensitivity analysis include leave-one-out analysis, meta-analysis using different model (such as fixed and random), and re-meta-analysis of some high-quality individual studies.

(11) **PRISMA #14 (Reporting bias assessment) and #21 (Reporting biases):** The PRISMA #14 and #21 items are considered as fulfilled when the methodology and results for reporting bias assessment are described. Otherwise, the PRISMA #14 is considered as fulfilled when an explanation for why the reporting bias is not assessed (such as, less than 5 included studies), and the PRISMA #21 is also considered as fulfilled (even though there is no results for reporting bias provided in the study).

(12) **PRISMA #16b (Study selection – exclusion studies):** A reference list of exclusion articles and corresponding reasons are needed for this item. Only simple number of articles with reason (usually displayed in the flowchart) are not enough.

(13) **PRISMA #23c (Discussion – limitation of review process):** The limitation of the review process (methodology) other than the limitations of the included studies, such as, language limitations, publication period limitations, limited number of searched databases, not searching for gray literatures, the limitation of the risk of bias assessment tool, etc.

(14) **PRISMA #27 (Availability of data, code and other materials):** A link to data, code, and other materials are the most ideal presentation for this item, but it is also acceptable that a data availability declaration is provided as "All data generated or analyzed during this study are included in this published article and its supplementary information files" or "The data that support the findings of this study are available from the corresponding author upon reasonable request".

2.2 AMSTAR-2 tool for study quality

The original version of AMSTAR tool has been published in 2007, as a practical critical appraisal tool for use by health professionals and policy makers who do not necessarily have advanced training in epidemiology, to enable them to carry out rapid and reproducible assessments of the quality of conduct of systematic reviews of randomized controlled trials of interventions. The AMSTAR version 2 underwent further development to enable appraisal of systematic reviews of randomized and non-randomized studies of healthcare interventions. The AMSTAR-2 tool retains 10 of the

original domains, has 16 items in total, has simpler response categories than the original AMSTAR, includes a more comprehensive user guide, and has an overall rating based on weaknesses in critical domains.

The following items were discussed to reach consensus:

(1) **AMSTAR #3 (Selection of study design):** The typical radiomics studies are diagnostic, predictive, or prognostic accuracy test studies. The study design can be randomized or non-randomized, cross-sectional or cohort, with or without interventions. We consider this item as "yes" if the study has declared that the study design selection, but the explanation of the is not necessary.

(2) **AMSTAR #4 (Search strategy):** This item is considered to be fulfilled when the detailed search string for each database is provided. Only one sentence describing the keyword applied or an example of search string in a specific database (such as PubMed) is not enough for this item. We decided to use this relatively strict criteria, because the search strategy can be easily provided as supplementary material.

(3) **AMSTAR #9 (Technique of RoB):** Although the systematic reviews in radiomics may employ multiple tools for risk of bias or quality assessment, including QUADAS-2, PROBAST, RQS, TRIPOD, CLAIM, and IBSI, the acceptable risk of bias tools for AMSTAR #9 are QUADAS-2 and PROBAST, which are designed for diagnostic, predictive, or prognosis modeling studies. The RQS and IBSI are considered as tools for methodology quality, the TRIPOD for reporting transparency, and the CLAIM for both methodology quality and reporting transparency.

(4) **AMSTAR #12 (Assessment of impact of RoB), and #13 (Discussion of impact of RoB):** These two items emphasized on the assessment of impact of RoB on the summary estimates, such as sensitivity analysis excluding studies with high risk of bias); while the discussion on the potential impact of RoB on the summary estimates, which does not ask for quantitative evaluations, and can be fulfilled by qualitative descriptions.

2.3 ROBIS tool for risk of bias

The three-phase ROBIS tool is specifically designed to assess the risk of bias in systematic reviews covering four domains: study eligibility criteria, identification and selection of studies, data collection and study appraisal, and synthesis and findings.

The following items were discussed to reach consensus:

(1) **ROBIS #1.1 (Did the review adhere to pre-defined objectives and eligibility criteria?):** This item was rated as "yes", if the protocol was provided, and the protocol was adhered; as "probably yes", if the aim and eligibility criteria of systematic review was provided in the article (usually provided), and they were adhered. Since the aim and eligibility criteria of systematic review was provided on some degree, there were no review rated as "probably no" or "no".

(2) **ROBIS #1.2 (Were the eligibility criteria appropriate for the review question?) & #1.3 (Were eligibility criteria unambiguous?):** These two items were assessed together. For ROBIS #1.2, we emphasized the relation between the criteria and the review question. For ROBIS #1.3, we emphasized the details of the criteria themselves. Since the raters are likely to require some content knowledge to answer this question, these items were assessed by one radiologist and one clinical with experience in related field.

(3) **ROBIS #1.4 (Were all restrictions in eligibility criteria based on study characteristics appropriate?) & #1.5 (Were any restrictions in eligibility criteria based on sources of information appropriate?):** These two items were assessed together. For ROBIS #1.4, the restrictions on study characteristics were usually not reported, and can be assumed that none were imposed, and the question was be answered as "Yes"; if there was any restriction on study characteristics, two raters would assess its appropriateness. For ROBIS #1.5, there were usually restrictions

on languages, this item was rated as “probably yes” if no reason for this restriction was provided or discussed in the limitation; if there was some reasoning or discussions, this item was rated as “yes”.

(4) **ROBIS #2.1 (Did the search include an appropriate range of databases/ electronic sources for published and unpublished reports?), #2.2 (Were methods additional to database searching used to identify relevant reports?), & #2.3 (Were the terms and structure of the search strategy likely to retrieve as many eligible studies as possible?):** These three items were assessed together. For ROBIS #2.1, this item was rated as “probably yes” if at least two databases were searched; and rated as “yes” if more searching was performed. For ROBIS #2.2, this item was rated as “probably yes” if at least one of following method was applied: citation searches, contacting experts, reference checking, handsearching, etc.; and rated as “yes” if multiple methods were used. For ROBIS #2.3, this item was rated as “yes” if the search strategy for each database was provided; and rated as “probably yes” if the keywords for searching was provided.

(5) **ROBIS #2.4 (Were restrictions based on date, publication format, or language appropriate?):** The guideline for ROBIS assessment declared that “restriction of papers based on language (e. g. restriction to English language articles) or publication format (e.g. restriction to full text published studies) is rarely (if ever) appropriate”. However, it is rarely possible for the review group to read the publications in all the languages. We rated this item as “probably yes”, for those has restrictions on language. The publication format was usually restricted to full-length articles in reviews to allow assessment on methodology quality and risk of bias; therefore, we did not rate the reviews with restrictions on publication format as “No”, but as “probably yes”.

(6) **ROBIS #2.5 (Were efforts made to minimise errors in selection of studies?):** This item was rated as “yes”, if at least two raters assessed the eligibility of studies independently; or it was rated as “probably no”. We did not used the “probably yes”, because it is hard to guess whether the process was independent if it is not reported in the article.

(7) **ROBIS #2.5 (Were efforts made to minimise errors in selection of studies?):** This item was rated as “yes”, if at least two raters assessed the eligibility of studies independently; or it was rated as “probably no”. We did not used the “probably yes”, because it is hard to guess whether the process was independent if it is not reported in the article (PRISMA #8).

(8) **ROBIS #3.1 (Were efforts made to minimise error in data collection?):** This item was rated as “yes”, if at least two raters extracted the data independently; or it was rated as “probably no”. We did not used the “probably yes”, because it is hard to guess whether the process was independent if it is not reported in the article (PRISMA #9).

(9) **ROBIS #3.2 (Were sufficient study characteristics available for both review authors and readers to be able to interpret the results?):** This question can be difficult to judge as all information collected as part of a review is not always presented in a publication, often due to space restrictions. Assessors may therefore need to access additional resources such as web appendices. We checked the supplementary materials and data availability declarations to allow a better assessment on this item. This item was rated as “probably yes” if there was study characteristics provided, and “yes” if there were more detailed information in extra materials or available from authors.

(10) **ROBIS #3.3 (Were all relevant study results collected for use in the synthesis?):** For a review to answer “Yes” to this question detailed information should be included in the methods section to describe how results data that were not reported in the format required for synthesis were obtained e.g. by estimating/transforming from reported data or by contacting authors for additional information. We referenced to PRISMA #13a and #13b for this item.

(11) **ROBIS #3.4 (Was risk of bias (or methodological quality) formally assessed using appropriate criteria?) & #3.5 (Were efforts made to minimise error in risk of bias assessment?):** These two items were assessed together. For ROBIS #3.4, the item was rated as “no” if no risk of bias assessment was performed; as “yes” if QUADAS-2 or

PROBAST tool was applied, because the systematic reviews included for current overview of systematic reviews were all for diagnostic, predictive, or prognostic purpose. For ROBIS #3.5, This item was rated as “yes”, if at least two raters assessed the risk of bias independently; or it was rated as “probably no”. We did not used the “probably yes”, because it is hard to guess whether the process was independent if it is not reported in the article (PRISMA #11).

(12)**ROBIS #4.1 (Did the synthesis include all studies that it should?):** This item was rated as “probably yes” if there was no evidence of the reviewers have purposefully excluded the results; and rated as “yes” if the method for reconstruction data were reported to allow the reviewers to gain the specific results from an included study are not available or reported.

(13)**ROBIS #4.2 (Were all predefined analyses followed or departures explained?):** We compare the analysis method and the results reported in the review, and rated it as “probably yes” if they matched to each other. For those pre-defined analysis not available, we considered they were fulfilled if there was a reasoning for it. For lacking of any pre-defined analysis, this item was rated as “no”.

(14)**ROBIS #4.3 (Was the synthesis appropriate given the nature and similarity in the research questions, study designs and outcomes across included studies?):** If a quantitative synthesis is undertaken (a meta-analysis), this question both addresses whether it was appropriate to do this and addresses the statistical methods used. Because we only included systematic reviews with meta-analysis the initial rating for all the systematic reviews were “probably yes”. The rating would be up-rated to “yes” if the selection of method for meta-analysis was discussed (PRISMA #13d)

(15)**ROBIS #4.4 (Was between-studies variation (heterogeneity) minimal or addressed in the synthesis?) & #4.5 (Were the findings robust, e. g. as demonstrated through funnel plot or sensitivity analyses?):** For ROBIS #4.4, if the source of heterogeneity was assessed, this item was rated as “yes”; otherwise, as “no” (PRISMA #13e). For ROBIS #4.5, if one of funnel plot or sensitivity analyses were performed, this item was rated as “yes”; otherwise, as “no” (PRISMA #13f or #14).

(16)**ROBIS #4.6 (Were biases in primary studies minimal or addressed in the synthesis?):** Examples of when this question would be answered “yes” might include all studies having received a “low risk of bias” rating from the reviewer or sensitivity analyses/ adjustment approaches were employed where studies were at high risk of bias. This item was rated as “no” if there was no risk of bias assessment (ROBIS #3.4). There was no all low risk systematic reviews; therefore, the sensitivity analysis was necessary for “probably yes” for this item (PRISMA #13f).

(17)**ROBIS #A (Did the interpretation of findings address all of the concerns identified the Phase 2 assessment?):** There was no systematic reviews rated as “low concern” in all domains. This item was rated as “yes” if all “high concern” or “unclear concern” domains have appropriately addressed concerns identified; or “probably yes” if at least one of high concern” or “unclear concern” domains have appropriately addressed concerns identified.

(18)**ROBIS #B (Was the relevance of identified studies to the review's research question appropriately considered?):** This item was rated as “yes”, if QUADAS-2 or PROBAST tool has been applied (ROBIS #3.4), or as “probably yes” if it was considered to be appropriate by the reviewers; or “probably no” if. It was probably not appropriate.

(19)**ROBIS #C (Did the reviewers avoid emphasizing results on the basis of their statistical significance?):** If there was only one meta-analysis performed in the review, this item was rated as “probably yes”, if there were multiple meta-analyses, this item was rated by the reviewers. If there was no evidence for highlighting results on the basis of their statistical significance, this item was rated as “probably yes”, if the review provided a comprehensive results for the meta-analyses, this item was rated as “yes”.

Supplementary Note S4 Data synthesis and analysis methods

The statistical analysis was performed with R language version 4.1.3 within using relevant packages. The meta-analyses were re-performed with R language version 4.1.3 using relevant package (metaumbrella) via a website App (https://www.metaumbrella.org). The data analysis was performed by a reviewer (J.Z.) under supervision of a statistical expert (J.L.).

1. Statistical analysis

The statistical analysis was performed with R language version 4.1.3 within using relevant packages. The adherence rate of PRISMA checklist was calculated as the deviation of the obtainable items to the obtained items. For example, if one systematic review obtained 30 items out of 52 obtainable items, the PRISMA adherence rate was 30 / 52 = 58%. The differences of PRISMA adherence rate, AMSTAR-2 rating, and ROBINS assessment were compared journal type (imaging or non-imaging), first authorship (radiologist or non-radiologist), biomarker (diagnostic, predictive, or prognostic), and publication year (2020, 2021, or 2022), using student t-test or one-way ANOVA, or chi-square test. A two-tailed $p < 0.05$ was recognized as statistical significance, unless specified otherwise.

2. Meta-analysis

2.1 Effect size

The odds ratio (OR) and the corresponding 95% confidence interval (CI) were pooled as summary effect size using random-effect models and corresponding p values were calculated. This function is available using the metaumbralla package. Because included studies were diagnostic accuracy tests, the diagnostic odds ratio and their 95% CIs were calculated to derive the effect size using a bivariate random effect model.

2.2 Heterogeneity

The Cochran's Q test and the I^2 statistic were used to assess heterogeneity among primary studies. Measuring inter-study dispersion assumes that, if all studies were methodologically identical and variation in results were only due to the random selection of study participants, the effect sizes would follow a chi-squared distribution. Cochran's Q assesses the hypothesis that the distribution of results is homogenous and p-values < 0.05 would generally lead to the rejection of this null-hypothesis. As with a small number of studies Cochran's Q can be distorted, I^2, a measure for how much of the variability between effect size estimates is due to methodological heterogeneity rather than sampling error, was also reported. I^2 values of 50% and less are usually considered to be low or unimportant, while above 50% are considered high. This function is available using the metaumbralla package.

2.3 Prediction intervals

The 95% prediction intervals were calculated to facilitate more conservative prediction for potential application of radiomics models (if the number of studies is equal or larger to 3). This function is available using the metaumbralla package. A "notnull" value for the 95% prediction interval of the meta-analysis to exclude the null value to achieve the class for which it is indicated.

2.4 Small-study effects

The Egger's test was conducted for small-study effects. When a two-tailed $p < 0.10$ was reached, small-study effect was considered detected. This function is available using the metaumbralla package.

2.5 Excess significance bias

Excess significance bias was evaluated by a chi-square test comparing the actual observed number of primary studies with a $p < 0.05$ with the expected number of primary studies with statistical significance. The expected number was the sum of statistical power estimates of each primary study in the meta-analysis. The presence of excess significance bias was proved when observed number > expected number and $p < 0.10$ for chi-square test were both reached. This function is available using the metaumbralla package.

3. Level of evidence

The strength of evidence supporting radiomics for clinical use were categorized into five levels: convincing, highly suggestive, suggestive, weak, and not suggestive (Supplementary Table S6). The rating was based on the results of a series of aforementioned analyses. This function is available using the metaumbralla package.

The criteria were strongly recommended to be used to all allow an objective, standardized classification of the level of evidence. However, the analysts should not forget that the variables used in these criteria are continuous and the set of cut-off points are only cut-off points. For example, the difference between a factor that includes 1000 patients and a factor that includes 1001 patients is negligible, but according to the criteria, the former can only be class IV (weak), whereas the latter could be class I (convincing).

Supplementary Note S5 List of included full-texts and excluded full-texts with justifications

List of included full-texts - in primary search [Reason for exclusion from meta-analysis]

1. Bedrikovetski S, Dudi-Venkata NN, Kroon HM, Seow W, Vather R, Carneiro G, Moore JW, Sammour T. Artificial intelligence for pre-operative lymph node staging in colorectal cancer: a systematic review and meta-analysis. BMC Cancer. 2021 Sep 26;21(1):1058. doi: 10.1186/s12885-021-08773-w. PMID: 34565338; PMCID: PMC8474828.
2. Bedrikovetski S, Dudi-Venkata NN, Maicas G, Kroon HM, Seow W, Carneiro G, Moore JW, Sammour T. Artificial intelligence for the diagnosis of lymph node metastases in patients with abdominopelvic malignancy: A systematic review and meta-analysis. Artif Intell Med. 2021 Mar;113:102022. doi: 10.1016/j.artmed.2021.102022. Epub 2021 Feb 2. PMID: 33685585.
3. Bhandari AP, Liong R, Koppen J, Murthy SV, Lasocki A. Noninvasive Determination of *IDH*and 1p19q Status of Lower-grade Gliomas Using MRI Radiomics: A Systematic Review. AJNR Am J Neuroradiol. 2021 Jan;42(1):94-101. doi: 10.3174/ajnr.A6875. Epub 2020 Nov 26. PMID: 33243896; PMCID: PMC7814803. [Two-by-two data not available, only AUC]
4. Cao X, et al. CT radiomics for predicting pathological grade of renal clear cell carcinoma:Meta-analysis. Chin J Med Imaging Technol, 2022, 38 (8):1197-1202. Doi: 10.13929/j.isn.1003-3289.2022.08.017.
5. Castaldo R, Cavaliere C, Soricelli A, Salvatore M, Pecchia L, Franzese M. Radiomic and Genomic Machine Learning Method Performance for Prostate Cancer Diagnosis: Systematic Literature Review. J Med Internet Res. 2021 Apr 1;23(4):e22394. doi: 10.2196/22394. PMID: 33792552; PMCID: PMC8050752.
6. Chen Q, Zhang L, Mo X, You J, Chen L, Fang J, Wang F, Jin Z, Zhang B, Zhang S. Current status and quality of radiomic studies for predicting immunotherapy response and outcome in patients with non-small cell lung cancer: a systematic review and meta-analysis. Eur J Nucl Med Mol Imaging. 2021 Dec;49(1):345-360. doi: 10.1007/s00259-021-05509-7. Epub 2021 Aug 17. PMID: 34402924.
7. Cleere EF, Davey MG, O'Neill S, Corbett M, O'Donnell JP, Hacking S, Keogh IJ, Lowery AJ, Kerin MJ. Radiomic Detection of Malignancy within Thyroid Nodules Using Ultrasonography-A Systematic Review and Meta-Analysis. Diagnostics (Basel). 2022 Mar 24;12(4):794. doi: 10.3390/diagnostics12040794. PMID: 35453841; PMCID: PMC9027085.
8. Davey MS, Davey MG, Ryan ÉJ, Hogan AM, Kerin MJ, Joyce M. The use of radiomic analysis of magnetic resonance imaging in predicting distant metastases of rectal carcinoma following surgical resection: A systematic review and meta-analysis. Colorectal Dis. 2021 Dec;23(12):3065-3072. doi: 10.1111/codi.15919. Epub 2021 Oct 1. PMID: 34536962.
9. Davey MG, Davey MS, Ryan ÉJ, Boland MR, McAnena PF, Lowery AJ, Kerin MJ. Is radiomic MRI a feasible alternative to OncotypeDX® recurrence score testing? A systematic review and meta-analysis. BJS Open. 2021 Sep 6;5(5):zrab081. doi: 10.1093/bjsopen/zrab081. PMID: 34633438; PMCID: PMC8504445.
10. Davey MG, Davey MS, Boland MR, Ryan ÉJ, Lowery AJ, Kerin MJ. Radiomic differentiation of breast cancer molecular subtypes using pre-operative breast imaging - A systematic review and meta-analysis. Eur J Radiol. 2021 Nov;144:109996. doi: 10.1016/j.ejrad.2021.109996. Epub 2021 Oct 2. PMID: 34624649.
11. Deantonio L, Garo ML, Paone G, Valli MC, Cappio S, La Regina D, Cefali M, Palmarocchi MC, Vannelli A, De Dosso S. 18F-FDG PET Radiomics as Predictor of Treatment Response in Oesophageal Cancer: A Systematic Review and Meta-Analysis. Front Oncol. 2022 Mar 15;12:861638. doi: 10.3389/fonc.2022.861638. PMID: 35371989; PMCID: PMC8965232. [Two-by-two data not available, only AUC]

12. Gao Y, Cheng S, Zhu L, Wang Q, Deng W, Sun Z, Wang S, Xue H. A systematic review of prognosis predictive role of radiomics in pancreatic cancer: heterogeneity markers or statistical tricks? Eur Radiol. 2022 Jul 29. doi: 10.1007/s00330-022-08922-0. Epub ahead of print. PMID: 35904618. [Methodology not available, C-index]
13. Gao Z, et al. Diagnostic value of radiomics in glioblastoma: a meta-analysis. Chinese Journal of Evidence-Based Medicine 2022 22:2 (232-242). Doi: 10.7507/1672-2531.202108134
14. Han Z, Chen Q, Zhang L, Mo X, You J, Chen L, Fang J, Wang F, Jin Z, Zhang S, Zhang B. Radiogenomic association between the T2-FLAIR mismatch sign and IDH mutation status in adult patients with lower-grade gliomas: an updated systematic review and meta-analysis. Eur Radiol. 2022 Aug;32(8):5339-5352. doi: 10.1007/s00330-022-08607-8. Epub 2022 Feb 15. PMID: 35169897.
15. Huang J, Tian W, Zhang L, Huang Q, Lin S, Ding Y, Liang W, Zheng S. Preoperative Prediction Power of Imaging Methods for Microvascular Invasion in Hepatocellular Carcinoma: A Systemic Review and Meta-Analysis. Front Oncol. 2020 Jun 26;10:887. doi: 10.3389/fonc.2020.00887. PMID: 32676450; PMCID: PMC7333535.
16. Huang H, Wang FF, Luo S, Chen G, Tang G. Diagnostic performance of radiomics using machine learning algorithms to predict MGMT promoter methylation status in glioma patients: a meta-analysis. Diagn Interv Radiol. 2021 Nov;27(6):716-724. doi: 10.5152/dir.2021.21153. PMID: 34792025; PMCID: PMC8621632.
17. Kao YS, Hsu Y. A Meta-Analysis for Using Radiomics to Predict Complete Pathological Response in Esophageal Cancer Patients Receiving Neoadjuvant Chemoradiation. In Vivo. 2021 May-Jun;35(3):1857-1863. doi: 10.21873/invivo.12448. PMID: 33910873; PMCID: PMC8193315. [Two-by-two data not available, only AUC]
18. Kao YS, Lin KT. A Meta-Analysis of Computerized Tomography-Based Radiomics for the Diagnosis of COVID-19 and Viral Pneumonia. Diagnostics (Basel). 2021 May 29;11(6):991. doi: 10.3390/diagnostics11060991. PMID: 34072573; PMCID: PMC8229671.
19. Kao YS, Lin KT. A meta-analysis of the diagnostic test accuracy of CT-based radiomics for the prediction of COVID-19 severity. Radiol Med. 2022 Jul;127(7):754-762. doi: 10.1007/s11547-022-01510-8. Epub 2022 Jun 22. PMID: 35731375; PMCID: PMC9213649.
20. Kothari G, Korte J, Lehrer EJ, Zaorsky NG, Lazarakis S, Kron T, Hardcastle N, Siva S. A systematic review and meta-analysis of the prognostic value of radiomics based models in non-small cell lung cancer treated with curative radiotherapy. Radiother Oncol. 2021 Feb;155:188-203. doi: 10.1016/j.radonc.2020.10.023. Epub 2020 Oct 21. PMID: 33096167.
21. Kozikowski M, Suarez-Ibarrola R, Osiecki R, Bilski K, Gratzke C, Shariat SF, Miernik A, Dobruch J. Role of Radiomics in the Prediction of Muscle-invasive Bladder Cancer: A Systematic Review and Meta-analysis. Eur Urol Focus. 2022 May;8(3):728-738. doi: 10.1016/j.euf.2021.05.005. Epub 2021 Jun 5. PMID: 34099417. [Methodology not available, C-index]
22. Lee S, Choi Y, Seo MK, Jang J, Shin NY, Ahn KJ, Kim BS. Magnetic Resonance Imaging-Based Radiomics for the Prediction of Progression-Free Survival in Patients with Nasopharyngeal Carcinoma: A Systematic Review and Meta-Analysis. Cancers (Basel). 2022 Jan 27;14(3):653. doi: 10.3390/cancers14030653. PMID: 35158921; PMCID: PMC8833585. [Methodology not available, C-index]
23. Li L, Wu C, Huang Y, Chen J, Ye D, Su Z. Radiomics for the Preoperative Evaluation of Microvascular Invasion in Hepatocellular Carcinoma: A Meta-Analysis. Front Oncol. 2022 Apr 7;12:831996. doi: 10.3389/fonc.2022.831996. PMID: 35463303; PMCID: PMC9021380.
24. Li L, Zhang J, Zhe X, Tang M, Zhang X, Lei X, Zhang L. A meta-analysis of MRI-based radiomic features for predicting lymph node metastasis in patients with cervical cancer. Eur J Radiol. 2022 Jun;151:110243. doi: 10.1016/j.ejrad.2022.110243. Epub 2022 Mar 9. PMID: 35366583.

25. Li Y, Liu Y, Liang Y, Wei R, Zhang W, Yao W, Luo S, Pang X, Wang Y, Jiang X, Lai S, Yang R. Radiomics can differentiate high-grade glioma from brain metastasis: a systematic review and meta-analysis. Eur Radiol. 2022 May 19. doi: 10.1007/s00330-022-08828-x. Epub ahead of print. PMID: 35587827.
26. Li Z, Ye J, Du H, Cao Y, Wang Y, Liu D, Zhu F, Shen H. Preoperative Prediction Power of Radiomics for Breast Cancer: A Systemic Review and Meta-Analysis. Front Oncol. 2022 Mar 1;12:837257. doi: 10.3389/fonc.2022.837257. PMID: 35299744; PMCID: PMC8920972.
27. Liang X, Yu X, Gao T. Machine learning with magnetic resonance imaging for prediction of response to neoadjuvant chemotherapy in breast cancer: A systematic review and meta-analysis. Eur J Radiol. 2022 May;150:110247. doi: 10.1016/j.ejrad.2022.110247. Epub 2022 Mar 10. PMID: 35290910. [Two-by-two data not available, only AUC]
28. Mühlbauer J, Egen L, Kowalewski KF, Grilli M, Walach MT, Westhoff N, Nuhn P, Laqua FC, Baessler B, Kriegmair MC. Radiomics in Renal Cell Carcinoma-A Systematic Review and Meta-Analysis. Cancers (Basel). 2021 Mar 17;13(6):1348. doi: 10.3390/cancers13061348. PMID: 33802699; PMCID: PMC8002585.
29. Pesapane F, Agazzi GM, Rotili A, Ferrari F, Cardillo A, Penco S, Dominelli V, D'Ecclesiis O, Vignati S, Raimondi S, Bozzini A, Pizzamiglio M, Petralia G, Nicosia L, Cassano E. Prediction of the Pathological Response to Neoadjuvant Chemotherapy in Breast Cancer Patients With MRI-Radiomics: A Systematic Review and Meta-analysis. Curr Probl Cancer. 2022 Jul 21;46(5):100883. doi: 10.1016/j.currproblcancer.2022.100883. Epub ahead of print. PMID: 35914383. [Two-by-two data not available, only AUC]
30. Ren J, Li Y, Liu XY, Zhao J, He YL, Jin ZY, Xue HD. Diagnostic performance of ADC values and MRI-based radiomics analysis for detecting lymph node metastasis in patients with cervical cancer: A systematic review and meta-analysis. Eur J Radiol. 2022 Sep 10;156:110504. doi: 10.1016/j.ejrad.2022.110504. Epub ahead of print. PMID: 36108474.
31. Sha YS, Chen JF. MRI-based radiomics for the diagnosis of triple-negative breast cancer: a meta-analysis. Clin Radiol. 2022 Sep;77(9):655-663. doi: 10.1016/j.crad.2022.04.015. Epub 2022 May 28. PMID: 35641339.
32. Sohn CK, Bisdas S. Diagnostic Accuracy of Machine Learning-Based Radiomics in Grading Gliomas: Systematic Review and Meta-Analysis. Contrast Media Mol Imaging. 2020 Dec 18;2020:2127062. doi: 10.1155/2020/2127062. PMID: 33746649; PMCID: PMC7952179.
33. Ugga L, Perillo T, Cuocolo R, Stanzione A, Romeo V, Green R, Cantoni V, Brunetti A. Meningioma MRI radiomics and machine learning: systematic review, quality score assessment, and meta-analysis. Neuroradiology. 2021 Aug;63(8):1293-1304. doi: 10.1007/s00234-021-02668-0. Epub 2021 Mar 2. PMID: 33649882; PMCID: PMC8295153. [Two-by-two data not available, only AUC]
34. Ursprung S, Beer L, Bruining A, Woitek R, Stewart GD, Gallagher FA, Sala E. Radiomics of computed tomography and magnetic resonance imaging in renal cell carcinoma-a systematic review and meta-analysis. Eur Radiol. 2020 Jun;30(6):3558-3566. doi: 10.1007/s00330-020-06666-3. Epub 2020 Feb 14. PMID: 32060715; PMCID: PMC7248043.
35. Yang C, Jiang Z, Cheng T, Zhou R, Wang G, Jing D, Bo L, Huang P, Wang J, Zhang D, Jiang J, Wang X, Lu H, Zhang Z, Li D. Radiomics for Predicting Response of Neoadjuvant Chemotherapy in Nasopharyngeal Carcinoma: A Systematic Review and Meta-Analysis. Front Oncol. 2022 May 4;12:893103. doi: 10.3389/fonc.2022.893103. PMID: 35600395; PMCID: PMC9121398.
36. Zhang J, Huang S, Xu Y, Wu J. Diagnostic Accuracy of Artificial Intelligence Based on Imaging Data for Preoperative Prediction of Microvascular Invasion in Hepatocellular Carcinoma: A Systematic Review and Meta-Analysis. Front Oncol. 2022 Feb 24;12:763842. doi: 10.3389/fonc.2022.763842. PMID: 35280776; PMCID: PMC8907853.

37. Zhang J, Li L, Zhe X, Tang M, Zhang X, Lei X, Zhang L. The Diagnostic Performance of Machine Learning-Based Radiomics of DCE-MRI in Predicting Axillary Lymph Node Metastasis in Breast Cancer: A Meta-Analysis. Front Oncol. 2022 Feb 4;12:799209. doi: 10.3389/fonc.2022.799209. PMID: 35186739; PMCID: PMC8854258.
38. Zhang H, Lei H, Pang J. Diagnostic performance of radiomics in adrenal masses: A systematic review and meta-analysis. Front Oncol. 2022 Sep 2;12:975183. doi: 10.3389/fonc.2022.975183. PMID: 36119492; PMCID: PMC9478189.
39. Zhong J, Hu Y, Si L, Jia G, Xing Y, Zhang H, Yao W. A systematic review of radiomics in osteosarcoma: utilizing radiomics quality score as a tool promoting clinical translation. Eur Radiol. 2021 Mar;31(3):1526-1535. doi: 10.1007/s00330-020-07221-w. Epub 2020 Sep 2. PMID: 32876837.
40. Zhong J, Hu Y, Ge X, Xing Y, Ding D, Zhang G, Zhang H, Yang Q, Yao W. A systematic review of radiomics in chondrosarcoma: assessment of study quality and clinical value needs handy tools. Eur Radiol. 2022 Aug 26. doi: 10.1007/s00330-022-09060-3. Epub ahead of print. PMID: 36018355.
41. Zhong J, Hu Y, Xing Y, Ge X, Ding D, Zhang H, Yao W. A systematic review of radiomics in pancreatitis: applying the evidence level rating tool for promoting clinical transferability. Insights Imaging. 2022 Aug 20;13(1):139. doi: 10.1186/s13244-022-01279-4. PMID: 35986798; PMCID: PMC9391628.
42. Zhong J, Hu Y, Zhang G, Xing Y, Ding D, Ge X, Pan Z, Yang Q, Yin Q, Zhang H, Zhang H, Yao W. An updated systematic review of radiomics in osteosarcoma: utilizing CLAIM to adapt the increasing trend of deep learning application in radiomics. Insights Imaging. 2022 Aug 20;13(1):138. doi: 10.1186/s13244-022-01277-6. PMID: 35986808; PMCID: PMC9392674.
43. Zhong X, Long H, Su L, Zheng R, Wang W, Duan Y, Hu H, Lin M, Xie X. Radiomics models for preoperative prediction of microvascular invasion in hepatocellular carcinoma: a systematic review and meta-analysis. Abdom Radiol (NY). 2022 Jun;47(6):2071-2088. doi: 10.1007/s00261-022-03496-3. Epub 2022 Apr 1. PMID: 35364684.

List of excluded full-texts (with justifications) - in primary search

1. Abdurixiti M, Nijiati M, Shen R, Ya Q, Abuduxiku N, Nijiati M. Current progress and quality of radiomic studies for predicting EGFR mutation in patients with non-small cell lung cancer using PET/CT images: a systematic review. Br J Radiol. 2021 Jun 1;94(1122):20201272. doi: 10.1259/bjr.20201272. Epub 2021 May 12. PMID: 33882244; PMCID: PMC8173688. (Without meta-analysis)
2. Abunahel BM, Pontre B, Kumar H, Petrov MS. Pancreas image mining: a systematic review of radiomics. Eur Radiol. 2021 May;31(5):3447-3467. doi: 10.1007/s00330-020-07376-6. Epub 2020 Nov 5. PMID: 33151391. (Without meta-analysis)
3. Azadikhah A, Varghese BA, Lei X, Martin-King C, Cen SY, Duddalwar VA. Radiomics quality score in renal masses: a systematic assessment on current literature. Br J Radiol. 2022 Sep 1;95(1137):20211211. doi: 10.1259/bjr.20211211. Epub 2022 Jun 15. PMID: 35671097. (Without meta-analysis)
4. Bezzi C, Mapelli P, Presotto L, Neri I, Scifo P, Savi A, Bettinardi V, Partelli S, Gianolli L, Falconi M, Picchio M. Radiomics in pancreatic neuroendocrine tumors: methodological issues and clinical significance. Eur J Nucl Med Mol Imaging. 2021 Nov;48(12):4002-4015. doi: 10.1007/s00259-021-05338-8. Epub 2021 Apr 9. PMID: 33835220. (Without meta-analysis)
5. Bhandari A, Ibrahim M, Sharma C, Liong R, Gustafson S, Prior M. CT-based radiomics for differentiating renal tumours: a systematic review. Abdom Radiol (NY). 2021 May;46(5):2052-2063. doi: 10.1007/s00261-020-02832-9. Epub 2020 Nov 2. PMID: 33136182. (Without meta-analysis)

6. Brancato V, Cerrone M, Lavitrano M, Salvatore M, Cavaliere C. A Systematic Review of the Current Status and Quality of Radiomics for Glioma Differential Diagnosis. Cancers (Basel). 2022 May 31;14(11):2731. doi: 10.3390/cancers14112731. PMID: 35681711; PMCID: PMC9179305. (Without meta-analysis)
7. Cacciamani, Giovanni E. et al. 'Radiomics and Bladder Cancer: Current Status'. 1 Jan. 2020 : 343 – 362. (Without meta-analysis)
8. Carbonara R, Bonomo P, Di Rito A, Didonna V, Gregucci F, Ciliberti MP, Surgo A, Bonaparte I, Fiorentino A, Sardaro A. Investigation of Radiation-Induced Toxicity in Head and Neck Cancer Patients through Radiomics and Machine Learning: A Systematic Review. J Oncol. 2021 Jun 9;2021:5566508. doi: 10.1155/2021/5566508. PMID: 34211551; PMCID: PMC8211491.(Without meta-analysis)
9. Casà C, Piras A, D'Aviero A, Preziosi F, Mariani S, Cusumano D, Romano A, Boskoski I, Lenkowicz J, Dinapoli N, Cellini F, Gambacorta MA, Valentini V, Mattiucci GC, Boldrini L. The impact of radiomics in diagnosis and staging of pancreatic cancer. Ther Adv Gastrointest Endosc. 2022 Mar 16;15:26317745221081596. doi: 10.1177/26317745221081596. PMID: 35342883; PMCID: PMC8943316. (Without meta-analysis)
10. Castello A, Castellani M, Florimonte L, Urso L, Mansi L, Lopci E. The Role of Radiomics in the Era of Immune Checkpoint Inhibitors: A New Protagonist in the Jungle of Response Criteria. J Clin Med. 2022 Mar 21;11(6):1740. doi: 10.3390/jcm11061740. PMID: 35330068; PMCID: PMC8948743. (Without meta-analysis)
11. Chang S, Han K, Suh YJ, Choi BW. Quality of science and reporting for radiomics in cardiac magnetic resonance imaging studies: a systematic review. Eur Radiol. 2022 Jul;32(7):4361-4373. doi: 10.1007/s00330-022-08587-9. Epub 2022 Mar 1. PMID: 35230519. (Without meta-analysis)
12. Chen Q, Zhang L, Liu S, You J, Chen L, Jin Z, Zhang S, Zhang B. Radiomics in precision medicine for gastric cancer: opportunities and challenges. Eur Radiol. 2022 Sep;32(9):5852-5868. doi: 10.1007/s00330-022-08704-8. Epub 2022 Mar 22. PMID: 35316364. (Without meta-analysis)
13. Chetan MR, Gleeson FV. Radiomics in predicting treatment response in non-small-cell lung cancer: current status, challenges and future perspectives. Eur Radiol. 2021 Feb;31(2):1049-1058. doi: 10.1007/s00330-020-07141-9. Epub 2020 Aug 18. PMID: 32809167; PMCID: PMC7813733. (Without meta-analysis)
14. Churchill, I.F., Sullivan, K.A., Simone, A.C. et al. Thoracic imaging radiomics for staging lung cancer: a systematic review and radiomic quality assessment. Clin Transl Imaging **10**, 191–216 (2022). https://doi.org/10.1007/s40336-021-00474-5 (Without meta-analysis)
15. Crimì F, Quaia E, Cabrelle G, Zanon C, Pepe A, Regazzo D, Tizianel I, Scaroni C, Ceccato F. Diagnostic Accuracy of CT Texture Analysis in Adrenal Masses: A Systematic Review. Int J Mol Sci. 2022 Jan 7;23(2):637. doi: 10.3390/ijms23020637. PMID: 35054823; PMCID: PMC8776161. (Without meta-analysis)
16. Crombé A, Fadli D, Italiano A, Saut O, Buy X, Kind M. Systematic review of sarcomas radiomics studies: Bridging the gap between concepts and clinical applications? Eur J Radiol. 2020 Nov;132:109283. doi: 10.1016/j.ejrad.2020.109283. Epub 2020 Sep 12. PMID: 32980727. (Without meta-analysis)
17. Darvish, L., Bahreyni-Toossi, MT., Roozbeh, N. *et al.* The role of radiogenomics in the diagnosis of breast cancer: a systematic review. *Egypt J Med Hum Genet* **23**, 99 (2022). https://doi.org/10.1186/s43042-022-00310-z (Without meta-analysis)
18. Di Re AM, Sun Y, Sundaresan P, Hau E, Toh JWT, Gee H, Or M, Haworth A. MRI radiomics in the prediction of therapeutic response to neoadjuvant therapy for locoregionally advanced rectal cancer: a systematic review. Expert Rev Anticancer Ther. 2021 Apr;21(4):425-449. doi: 10.1080/14737140.2021.1860762. Epub 2021 Jan 11. PMID: 33289435. (Without meta-analysis)

19. Du PT, et al. Pulmonary tuberculosis diagnosis, differentiation and disease management: A review of radiomics applications. Polish Journal of Medical Physics and Engineering 2021 27:4 (251-259). 10.2478/pjmpe-2021-0030 (Narrative review)
20. El Ayachy R, Giraud N, Giraud P, Durdux C, Giraud P, Burgun A, Bibault JE. The Role of Radiomics in Lung Cancer: From Screening to Treatment and Follow-Up. Front Oncol. 2021 May 5;11:603595. doi: 10.3389/fonc.2021.603595. PMID: 34026602; PMCID: PMC8131863. (Without meta-analysis)
21. Eldaly AS, Avila FR, Torres-Guzman RA, Maita K, Garcia JP, Forte AJ, Serrano LP. Radiomics And Artificial Intelligence In Predicting Axillary Lymph Node Metastasis In Breast Cancer: A Systematic Review. Curr Med Imaging. 2022 Aug 22. doi: 10.2174/1573405618666220822093226. Epub ahead of print. PMID: 35996255. (Without meta-analysis)
22. Faiella E, Santucci D, Calabrese A, Russo F, Vadalà G, Zobel BB, Soda P, Iannello G, de Felice C, Denaro V. Artificial Intelligence in Bone Metastases: An MRI and CT Imaging Review. Int J Environ Res Public Health. 2022 Feb 8;19(3):1880. doi: 10.3390/ijerph19031880. PMID: 35162902; PMCID: PMC8834956. (Without meta-analysis)
23. Fiz F, Jayakody Arachchige VS, Gionso M, Pecorella I, Selvam A, Wheeler DR, Sollini M, Viganò L. Radiomics of Biliary Tumors: A Systematic Review of Current Evidence. Diagnostics (Basel). 2022 Mar 28;12(4):826. doi: 10.3390/diagnostics12040826. PMID: 35453878; PMCID: PMC9024804. (Without meta-analysis)
24. Fiz F, Viganò L, Gennaro N, Costa G, La Bella L, Boichuk A, Cavinato L, Sollini M, Politi LS, Chiti A, Torzilli G. Radiomics of Liver Metastases: A Systematic Review. Cancers (Basel). 2020 Oct 7;12(10):2881. doi: 10.3390/cancers12102881. PMID: 33036490; PMCID: PMC7600822. (Without meta-analysis)
25. Gao C, Li J, Wu L, Kong D, Xu M, Zhou C. The Natural Growth of Subsolid Nodules Predicted by Quantitative Initial CT Features: A Systematic Review. Front Oncol. 2020 Mar 27;10:318. doi: 10.3389/fonc.2020.00318. PMID: 32292716; PMCID: PMC7119340. (Not radiomics)
26. Ge L, Chen Y, Yan C, Zhao P, Zhang P, A R, Liu J. Study Progress of Radiomics With Machine Learning for Precision Medicine in Bladder Cancer Management. Front Oncol. 2019 Nov 28;9:1296. doi: 10.3389/fonc.2019.01296. PMID: 31850202; PMCID: PMC6892826. (Without meta-analysis)
27. Ghezzo S, Bezzi C, Presotto L, Mapelli P, Bettinardi V, Savi A, Neri I, Preza E, Samanes Gajate AM, De Cobelli F, Scifo P, Picchio M. State of the art of radiomic analysis in the clinical management of prostate cancer: A systematic review. Crit Rev Oncol Hematol. 2022 Jan;169:103544. doi: 10.1016/j.critrevonc.2021.103544. Epub 2021 Nov 18. PMID: 34801699. (Without meta-analysis)
28. Giraud P, Giraud P, Gasnier A, El Ayachy R, Kreps S, Foy JP, Durdux C, Huguet F, Burgun A, Bibault JE. Radiomics and Machine Learning for Radiotherapy in Head and Neck Cancers. Front Oncol. 2019 Mar 27;9:174. doi: 10.3389/fonc.2019.00174. PMID: 30972291; PMCID: PMC6445892. (Narrative review)
29. Gitto S, Cuocolo R, Albano D, Morelli F, Pescatori LC, Messina C, Imbriaco M, Sconfienza LM. CT and MRI radiomics of bone and soft-tissue sarcomas: a systematic review of reproducibility and validation strategies. Insights Imaging. 2021 Jun 2;12(1):68. doi: 10.1186/s13244-021-01008-3. PMID: 34076740; PMCID: PMC8172744. (Without meta-analysis)
30. Gong XQ, Tao YY, Wu YK, Liu N, Yu X, Wang R, Zheng J, Liu N, Huang XH, Li JD, Yang G, Wei XQ, Yang L, Zhang XM. Progress of MRI Radiomics in Hepatocellular Carcinoma. Front Oncol. 2021 Sep 20;11:698373. doi: 10.3389/fonc.2021.698373. PMID: 34616673; PMCID: PMC8488263. (Narrative review)
31. Granzier RWY, van Nijnatten TJA, Woodruff HC, Smidt ML, Lobbes MBI. Exploring breast cancer response prediction to neoadjuvant systemic therapy using MRI-based radiomics: A systematic review. Eur J Radiol. 2019 Dec;121:108736. doi: 10.1016/j.ejrad.2019.108736. Epub 2019 Nov 6. PMID: 31734639. (Without meta-analysis)

32. Guglielmo P, Marturano F, Bettinelli A, Gregianin M, Paiusco M, Evangelista L. Additional Value of PET Radiomic Features for the Initial Staging of Prostate Cancer: A Systematic Review from the Literature. Cancers (Basel). 2021 Nov 30;13(23):6026. doi: 10.3390/cancers13236026. PMID: 34885135; PMCID: PMC8657371. (Without meta-analysis)
33. Guha A, Connor S, Anjari M, Naik H, Siddiqui M, Cook G, Goh V. Radiomic analysis for response assessment in advanced head and neck cancers, a distant dream or an inevitable reality? A systematic review of the current level of evidence. Br J Radiol. 2020 Feb 1;93(1106):20190496. doi: 10.1259/bjr.20190496. Epub 2019 Nov 6. PMID: 31682155; PMCID: PMC7055439. (Without meta-analysis)
34. Harding-Theobald E, Louissaint J, Maraj B, Cuaresma E, Townsend W, Mendiratta-Lala M, Singal AG, Su GL, Lok AS, Parikh ND. Systematic review: radiomics for the diagnosis and prognosis of hepatocellular carcinoma. Aliment Pharmacol Ther. 2021 Oct;54(7):890-901. doi: 10.1111/apt.16563. Epub 2021 Aug 12. PMID: 34390014; PMCID: PMC8435007. (Without meta-analysis)
35. Infante T, Cavaliere C, Punzo B, Grimaldi V, Salvatore M, Napoli C. Radiogenomics and Artificial Intelligence Approaches Applied to Cardiac Computed Tomography Angiography and Cardiac Magnetic Resonance for Precision Medicine in Coronary Heart Disease: A Systematic Review. Circ Cardiovasc Imaging. 2021 Dec;14(12):1133-1146. doi: 10.1161/CIRCIMAGING.121.013025. Epub 2021 Dec 17. PMID: 34915726. (Without meta-analysis)
36. Jansen RW, van Amstel P, Martens RM, Kooi IE, Wesseling P, de Langen AJ, Menke-Van der Houven van Oordt CW, Jansen BHE, Moll AC, Dorsman JC, Castelijns JA, de Graaf P, de Jong MC. Non-invasive tumor genotyping using radiogenomic biomarkers, a systematic review and oncology-wide pathway analysis. Oncotarget. 2018 Apr 13;9(28):20134-20155. doi: 10.18632/oncotarget.24893. PMID: 29732009; PMCID: PMC5929452. (Without meta-analysis)
37. Jethanandani A, Lin TA, Volpe S, Elhalawani H, Mohamed ASR, Yang P, Fuller CD. Exploring Applications of Radiomics in Magnetic Resonance Imaging of Head and Neck Cancer: A Systematic Review. Front Oncol. 2018 May 14;8:131. doi: 10.3389/fonc.2018.00131. PMID: 29868465; PMCID: PMC5960677. (Without meta-analysis)
38. Khaleel S, Katims A, Cumarasamy S, Rosenzweig S, Attalla K, Hakimi AA, Mehrazin R. Radiogenomics in Clear Cell Renal Cell Carcinoma: A Review of the Current Status and Future Directions. Cancers (Basel). 2022 Apr 22;14(9):2085. doi: 10.3390/cancers14092085. PMID: 35565216; PMCID: PMC9100795. (Without meta-analysis)
39. Kocak B, Durmaz ES, Erdim C, Ates E, Kaya OK, Kilickesmez O. Radiomics of Renal Masses: Systematic Review of Reproducibility and Validation Strategies. AJR Am J Roentgenol. 2020 Jan;214(1):129-136. doi: 10.2214/AJR.19.21709. Epub 2019 Oct 15. PMID: 31613661. (Without meta-analysis)
40. Koong K, Preda V, Jian A, Liquet-Weiland B, Di Ieva A. Application of artificial intelligence and radiomics in pituitary neuroendocrine and sellar tumors: a quantitative and qualitative synthesis. Neuroradiology. 2022 Apr;64(4):647-668. doi: 10.1007/s00234-021-02845-1. Epub 2021 Nov 27. PMID: 34839380. (Not radiomics)
41. La Greca Saint-Esteven A, Vuong D, Tschanz F, van Timmeren JE, Dal Bello R, Waller V, Pruschy M, Guckenberger M, Tanadini-Lang S. Systematic Review on the Association of Radiomics with Tumor Biological Endpoints. Cancers (Basel). 2021 Jun 16;13(12):3015. doi: 10.3390/cancers13123015. PMID: 34208595; PMCID: PMC8234501. (Without meta-analysis)
42. Laino ME, Viganò L, Ammirabile A, Lofino L, Generali E, Francone M, Lleo A, Saba L, Savevski V. The added value of artificial intelligence to LI-RADS categorization: A systematic review. Eur J Radiol. 2022 May;150:110251. doi: 10.1016/j.ejrad.2022.110251. Epub 2022 Mar 11. PMID: 35303556. (Without meta-analysis)

43. Lecointre L, Dana J, Lodi M, Akladios C, Gallix B. Artificial intelligence-based radiomics models in endometrial cancer: A systematic review. Eur J Surg Oncol. 2021 Nov;47(11):2734-2741. doi: 10.1016/j.ejso.2021.06.023. Epub 2021 Jun 24. PMID: 34183201. (Without meta-analysis)
44. Lee S, Han K, Suh YJ. Quality assessment of radiomics research in cardiac CT: a systematic review. Eur Radiol. 2022 May;32(5):3458-3468. doi: 10.1007/s00330-021-08429-0. Epub 2022 Jan 4. PMID: 34981135. (Without meta-analysis)
45. Li MD, Ahmed SR, Choy E, Lozano-Calderon SA, Kalpathy-Cramer J, Chang CY. Artificial intelligence applied to musculoskeletal oncology: a systematic review. Skeletal Radiol. 2022 Feb;51(2):245-256. doi: 10.1007/s00256-021-03820-w. Epub 2021 May 19. PMID: 34013447. (Not radiomics)
46. Liu X, Elbanan MG, Luna A, Haider MA, Smith AD, Sabottke CF, Spieler BM, Turkbey B, Fuentes D, Moawad A, Kamel S, Horvat N, Elsayes KM. Radiomics in Abdominopelvic Solid-Organ Oncologic Imaging: Current Status. AJR Am J Roentgenol. 2022 Jun 29. doi: 10.2214/AJR.22.27695. Epub ahead of print. PMID: 35766531. (Narrative review)
47. Midiri F, Vernuccio F, Purpura P, Alongi P, Bartolotta TV. Multiparametric MRI and Radiomics in Prostate Cancer: A Review of the Current Literature. Diagnostics (Basel). 2021 Oct 3;11(10):1829. doi: 10.3390/diagnostics11101829. PMID: 34679527; PMCID: PMC8534893. (Narrative review)
48. Mirón Mombiela R, Arildskov AR, Bruun FJ, Hasselbalch LH, Holst KB, Rasmussen SH, Borrás C. What Genetics Can Do for Oncological Imaging: A Systematic Review of the Genetic Validation Data Used in Radiomics Studies. Int J Mol Sci. 2022 Jun 10;23(12):6504. doi: 10.3390/ijms23126504. PMID: 35742947; PMCID: PMC9224495. (Without meta-analysis)
49. Moyya PD, Asaithambi M. Radiomics - Quantitative Biomarker Analysis for Breast Cancer Diagnosis and Prediction: A Review. Curr Med Imaging. 2022;18(1):3-17. doi: 10.2174/1573405617666210303102526. PMID: 33655872. (Without meta-analysis)
50. Nardone V, Reginelli A, Grassi R, Boldrini L, Vacca G, D'Ippolito E, Annunziata S, Farchione A, Belfiore MP, Desideri I, Cappabianca S. Delta radiomics: a systematic review. Radiol Med. 2021 Dec;126(12):1571-1583. doi: 10.1007/s11547-021-01436-7. Epub 2021 Dec 4. PMID: 34865190. (Without meta-analysis)
51. Ninatti G, Kirienko M, Neri E, Sollini M, Chiti A. Imaging-Based Prediction of Molecular Therapy Targets in NSCLC by Radiogenomics and AI Approaches: A Systematic Review. Diagnostics (Basel). 2020 May 30;10(6):359. doi: 10.3390/diagnostics10060359. PMID: 32486314; PMCID: PMC7345054. (Narrative review)
52. Oltra-Sastre M, Fuster-Garcia E, Juan-Albarracin J, Sáez C, Perez-Girbes A, Sanz-Requena R, Revert-Ventura A, Mocholi A, Urchueguia J, Hervas A, Reynes G, Font-de-Mora J, Muñoz-Langa J, Botella C, Aparici F, Marti-Bonmati L, Garcia-Gomez JM. Multi-parametric MR Imaging Biomarkers Associated to Clinical Outcomes in Gliomas: A Systematic Review. Curr Med Imaging Rev. 2019;15(10):933-947. doi: 10.2174/1573405615666190109100503. PMID: 32008521. (Not radiomics)
53. Park CJ, Park YW, Ahn SS, Kim D, Kim EH, Kang SG, Chang JH, Kim SH, Lee SK. Quality of Radiomics Research on Brain Metastasis: A Roadmap to Promote Clinical Translation. Korean J Radiol. 2022 Jan;23(1):77-88. doi: 10.3348/kjr.2021.0421. PMID: 34983096; PMCID: PMC8743155. (Without meta-analysis)
54. Park JE, Kim HS, Kim D, Park SY, Kim JY, Cho SJ, Kim JH. A systematic review reporting quality of radiomics research in neuro-oncology: toward clinical utility and quality improvement using high-dimensional imaging features. BMC Cancer. 2020 Jan 10;20(1):29. doi: 10.1186/s12885-019-6504-5. PMID: 31924170; PMCID: PMC6954557. (Without meta-analysis)

55. Phillips I, Ajaz M, Ezhil V, Prakash V, Alobaidli S, McQuaid SJ, South C, Scuffham J, Nisbet A, Evans P. Clinical applications of textural analysis in non-small cell lung cancer. Br J Radiol. 2018 Jan;91(1081):20170267. doi: 10.1259/bjr.20170267. Epub 2017 Oct 27. PMID: 28869399; PMCID: PMC5966204. (Narrative review)
56. Piñeiro-Fiel M, Moscoso A, Pubul V, Ruibal Á, Silva-Rodríguez J, Aguiar P. A Systematic Review of PET Textural Analysis and Radiomics in Cancer. Diagnostics (Basel). 2021 Feb 23;11(2):380. doi: 10.3390/diagnostics11020380. PMID: 33672285; PMCID: PMC7926413. (Without meta-analysis)
57. Ponsiglione A, Stanzione A, Cuocolo R, Ascione R, Gambardella M, De Giorgi M, Nappi C, Cuocolo A, Imbriaco M. Cardiac CT and MRI radiomics: systematic review of the literature and radiomics quality score assessment. Eur Radiol. 2022 Apr;32(4):2629-2638. doi: 10.1007/s00330-021-08375-x. Epub 2021 Nov 23. PMID: 34812912. (Without meta-analysis)
58. Guglielmo P, Marturano F, Bettinelli A, Gregianin M, Paiusco M, Evangelista L. Additional Value of PET Radiomic Features for the Initial Staging of Prostate Cancer: A Systematic Review from the Literature. Cancers (Basel). 2021 Nov 30;13(23):6026. doi: 10.3390/cancers13236026. PMID: 34885135; PMCID: PMC8657371. (Without meta-analysis)
59. Raisi-Estabragh Z, Izquierdo C, Campello VM, Martin-Isla C, Jaggi A, Harvey NC, Lekadir K, Petersen SE. Cardiac magnetic resonance radiomics: basic principles and clinical perspectives. Eur Heart J Cardiovasc Imaging. 2020 Apr 1;21(4):349-356. doi: 10.1093/ehjci/jeaa028. PMID: 32142107; PMCID: PMC7082724. (Narrative review)
60. Ramlee S, Hulse D, Bernatowicz K, Pérez-López R, Sala E, Aloj L. Radiomic Signatures Associated with $CD8^+$ Tumour-Infiltrating Lymphocytes: A Systematic Review and Quality Assessment Study. Cancers (Basel). 2022 Jul 27;14(15):3656. doi: 10.3390/cancers14153656. PMID: 35954318; PMCID: PMC9367613. (Without meta-analysis)
61. Ravegnini G, Ferioli M, Morganti AG, Strigari L, Pantaleo MA, Nannini M, De Leo A, De Crescenzo E, Coe M, De Palma A, De Iaco P, Rizzo S, Perrone AM. Radiomics and Artificial Intelligence in Uterine Sarcomas: A Systematic Review. J Pers Med. 2021 Nov 11;11(11):1179. doi: 10.3390/jpm11111179. PMID: 34834531; PMCID: PMC8624692. (Without meta-analysis)
62. Rizzo, A., Triumbari, E.K.A., Gatta, R. et al. The role of ^{18}F-FDG PET/CT radiomics in lymphoma. Clin Transl Imaging **9**, 589–598 (2021). https://doi.org/10.1007/s40336-021-00451-y (Without meta-analysis)
63. Rizzo S, Manganaro L, Dolciami M, Gasparri ML, Papadia A, Del Grande F. Computed Tomography Based Radiomics as a Predictor of Survival in Ovarian Cancer Patients: A Systematic Review. Cancers (Basel). 2021 Feb 2;13(3):573. doi: 10.3390/cancers13030573. PMID: 33540655; PMCID: PMC7867247. (Without meta-analysis)
64. Salvestrini V, Greco C, Guerini AE, Longo S, Nardone V, Boldrini L, Desideri I, De Felice F. The role of feature-based radiomics for predicting response and radiation injury after stereotactic radiation therapy for brain metastases: A critical review by the Young Group of the Italian Association of Radiotherapy and Clinical Oncology (yAIRO). Transl Oncol. 2022 Jan;15(1):101275. doi: 10.1016/j.tranon.2021.101275. Epub 2021 Nov 17. PMID: 34800918; PMCID: PMC8605350. (Without meta-analysis)
65. Sanduleanu S, Woodruff HC, de Jong EEC, van Timmeren JE, Jochems A, Dubois L, Lambin P. Tracking tumor biology with radiomics: A systematic review utilizing a radiomics quality score. Radiother Oncol. 2018 Jun;127(3):349-360. doi: 10.1016/j.radonc.2018.03.033. Epub 2018 May 18. PMID: 29779918. (Without meta-analysis)
66. Seow P, Wong JHD, Ahmad-Annuar A, Mahajan A, Abdullah NA, Ramli N. Quantitative magnetic resonance imaging and radiogenomic biomarkers for glioma characterisation: a systematic review. Br J Radiol. 2018

Dec;91(1092):20170930. doi: 10.1259/bjr.20170930. Epub 2018 Jun 29. PMID: 29902076; PMCID: PMC6319852. (Without meta-analysis)

67. Shang J, Guo Y, Ma Y, Hou Y. Cardiac computed tomography radiomics: a narrative review of current status and future directions. Quant Imaging Med Surg. 2022 Jun;12(6):3436-3453. doi: 10.21037/qims-21-1022. PMID: 35655815; PMCID: PMC9131324. (Narrative review)
68. Shi L, He Y, Yuan Z, Benedict S, Valicenti R, Qiu J, Rong Y. Radiomics for Response and Outcome Assessment for Non-Small Cell Lung Cancer. Technol Cancer Res Treat. 2018 Jan 1;17:1533033818782788. doi: 10.1177/1533033818782788. PMID: 29940810; PMCID: PMC6048673. (Narrative review)
69. Shi L, Zhao J, Peng X, Wang Y, Liu L, Sheng M. CT-based radiomics for differentiating invasive adenocarcinomas from indolent lung adenocarcinomas appearing as ground-glass nodules: Asystematic review. Eur J Radiol. 2021 Nov;144:109956. doi: 10.1016/j.ejrad.2021.109956. Epub 2021 Sep 17. PMID: 34563797. (Without meta-analysis)
70. Sollini M, Antunovic L, Chiti A, Kirienko M. Towards clinical application of image mining: a systematic review on artificial intelligence and radiomics. Eur J Nucl Med Mol Imaging. 2019 Dec;46(13):2656-2672. doi: 10.1007/s00259-019-04372-x. Epub 2019 Jun 18. PMID: 31214791; PMCID: PMC6879445. (Without meta-analysis)
71. Spadarella G, Calareso G, Garanzini E, Ugga L, Cuocolo A, Cuocolo R. MRI based radiomics in nasopharyngeal cancer: Systematic review and perspectives using radiomic quality score (RQS) assessment. Eur J Radiol. 2021 Jul;140:109744. doi: 10.1016/j.ejrad.2021.109744. Epub 2021 Apr 30. PMID: 33962253. (Without meta-analysis)
72. Spadarella G, Ugga L, Calareso G, Villa R, D'Aniello S, Cuocolo R. The impact of radiomics for human papillomavirus status prediction in oropharyngeal cancer: systematic review and radiomics quality score assessment. Neuroradiology. 2022 Aug;64(8):1639-1647. doi: 10.1007/s00234-022-02959-0. Epub 2022 Apr 23. PMID: 35459957; PMCID: PMC9271107. (Without meta-analysis)
73. Spohn SKB, Bettermann AS, Bamberg F, Benndorf M, Mix M, Nicolay NH, Fechter T, Hölscher T, Grosu R, Chiti A, Grosu AL, Zamboglou C. Radiomics in prostate cancer imaging for a personalized treatment approach - current aspects of methodology and a systematic review on validated studies. Theranostics. 2021 Jul 6;11(16):8027-8042. doi: 10.7150/thno.61207. PMID: 34335978; PMCID: PMC8315055. (Without meta-analysis)
74. Staal FCR, Aalbersberg EA, van der Velden D, Wilthagen EA, Tesselaar MET, Beets-Tan RGH, Maas M. GEP-NET radiomics: a systematic review and radiomics quality score assessment. Eur Radiol. 2022 Jul 26. doi: 10.1007/s00330-022-08996-w. Epub ahead of print. PMID: 35882634. (Without meta-analysis)
75. Staal FCR, van der Reijd DJ, Taghavi M, Lambregts DMJ, Beets-Tan RGH, Maas M. Radiomics for the Prediction of Treatment Outcome and Survival in Patients With Colorectal Cancer: A Systematic Review. Clin Colorectal Cancer. 2021 Mar;20(1):52-71. doi: 10.1016/j.clcc.2020.11.001. Epub 2020 Nov 7. PMID: 33349519. (Without meta-analysis)
76. Stanzione A, Galatola R, Cuocolo R, Romeo V, Verde F, Mainenti PP, Brunetti A, Maurea S. Radiomics in Cross-Sectional Adrenal Imaging: A Systematic Review and Quality Assessment Study. Diagnostics (Basel). 2022 Feb 24;12(3):578. doi: 10.3390/diagnostics12030578. PMID: 35328133; PMCID: PMC8947112. (Without meta-analysis)
77. Stanzione A, Gambardella M, Cuocolo R, Ponsiglione A, Romeo V, Imbriaco M. Prostate MRI radiomics: A systematic review and radiomic quality score assessment. Eur J Radiol. 2020 Aug;129:109095. doi: 10.1016/j.ejrad.2020.109095. Epub 2020 May 30. Erratum in: Eur J Radiol. 2020 Oct;131:109208. PMID: 32531722. (Without meta-analysis)

78. Stanzione A, Verde F, Cuocolo R, Romeo V, Paolo Mainenti P, Brunetti A, Maurea S. Placenta Accreta Spectrum Disorders and Radiomics: Systematic review and quality appraisal. Eur J Radiol. 2022 Aug 22;155:110497. doi: 10.1016/j.ejrad.2022.110497. Epub ahead of print. PMID: 36030661. (Without meta-analysis)
79. Subramanian H, Dey R, Brim WR, Tillmanns N, Cassinelli Petersen G, Brackett A, Mahajan A, Johnson M, Malhotra A, Aboian M. Trends in Development of Novel Machine Learning Methods for the Identification of Gliomas in Datasets That Include Non-Glioma Images: A Systematic Review. Front Oncol. 2021 Dec 23;11:788819. doi: 10.3389/fonc.2021.788819. PMID: 35004312; PMCID: PMC8733688. (Not radiomics)
80. Sugano D, Sanford D, Abreu A, Duddalwar V, Gill I, Cacciamani GE. Impact of radiomics on prostate cancer detection: a systematic review of clinical applications. Curr Opin Urol. 2020 Nov;30(6):754-781. doi: 10.1097/MOU.0000000000000822. PMID: 32941257. (Without meta-analysis)
81. Sushentsev N, Moreira Da Silva N, Yeung M, Barrett T, Sala E, Roberts M, Rundo L. Comparative performance of fully-automated and semi-automated artificial intelligence methods for the detection of clinically significant prostate cancer on MRI: a systematic review. Insights Imaging. 2022 Mar 28;13(1):59. doi: 10.1186/s13244-022-01199-3. PMID: 35347462; PMCID: PMC8960511. (Without meta-analysis)
82. Tabatabaei M, Razaei A, Sarrami AH, Saadatpour Z, Singhal A, Sotoudeh H. Current Status and Quality of Machine Learning-Based Radiomics Studies for Glioma Grading: A Systematic Review. Oncology. 2021;99(7):433-443. doi: 10.1159/000515597. Epub 2021 Apr 13. PMID: 33849021. (Without meta-analysis)
83. Vaidya T, Agrawal A, Mahajan S, Thakur MH, Mahajan A. The Continuing Evolution of Molecular Functional Imaging in Clinical Oncology: The Road to Precision Medicine and Radiogenomics (Part I). Mol Diagn Ther. 2019 Feb;23(1):1-26. doi: 10.1007/s40291-018-0366-4. PMID: 30411216. (Narrative review)
84. Valdora F, Houssami N, Rossi F, Calabrese M, Tagliafico AS. Rapid review: radiomics and breast cancer. Breast Cancer Res Treat. 2018 Jun;169(2):217-229. doi: 10.1007/s10549-018-4675-4. Epub 2018 Feb 2. PMID: 29396665. (Without meta-analysis)
85. van Kempen EJ, Post M, Mannil M, Kusters B, Ter Laan M, Meijer FJA, Henssen DJHA. Accuracy of Machine Learning Algorithms for the Classification of Molecular Features of Gliomas on MRI: A Systematic Literature Review and Meta-Analysis. Cancers (Basel). 2021 May 26;13(11):2606. doi: 10.3390/cancers13112606. PMID: 34073309; PMCID: PMC8198025. (Not radiomics)
86. Wakabayashi T, Ouhmich F, Gonzalez-Cabrera C, Felli E, Saviano A, Agnus V, Savadjiev P, Baumert TF, Pessaux P, Marescaux J, Gallix B. Radiomics in hepatocellular carcinoma: a quantitative review. Hepatol Int. 2019 Sep;13(5):546-559. doi: 10.1007/s12072-019-09973-0. Epub 2019 Aug 31. PMID: 31473947; PMCID: PMC7613479. (Without meta-analysis)
87. Walls GM, Osman SOS, Brown KH, Butterworth KT, Hanna GG, Hounsell AR, McGarry CK, Leijenaar RTH, Lambin P, Cole AJ, Jain S. Radiomics for Predicting Lung Cancer Outcomes Following Radiotherapy: A Systematic Review. Clin Oncol (R Coll Radiol). 2022 Mar;34(3):e107-e122. doi: 10.1016/j.clon.2021.10.006. Epub 2021 Nov 8. PMID: 34763965. (Without meta-analysis)
88. Wang H, Zhou Y, Li L, Hou W, Ma X, Tian R. Current status and quality of radiomics studies in lymphoma: a systematic review. Eur Radiol. 2020 Nov;30(11):6228-6240. doi: 10.1007/s00330-020-06927-1. Epub 2020 May 29. PMID: 32472274. (Without meta-analysis)
89. Wang Q, Li C, Zhang J, Hu X, Fan Y, Ma K, Sparrelid E, Brismar TB. Radiomics Models for Predicting Microvascular Invasion in Hepatocellular Carcinoma: A Systematic Review and Radiomics Quality Score Assessment. Cancers (Basel). 2021 Nov 22;13(22):5864. doi: 10.3390/cancers13225864. PMID: 34831018; PMCID: PMC8616379. (Without meta-analysis)

90. Wen Li Y, Leech M. Review of the Role of Radiomics in Tumour Risk Classification and Prognosis of Cancer. Anticancer Res. 2020 Jul;40(7):3605-3618. doi: 10.21873/anticanres.14350. PMID: 32620600. (Narrative review)
91. Wesdorp NJ, Hellingman T, Jansma EP, van Waesberghe JTM, Boellaard R, Punt CJA, Huiskens J, Kazemier G. Advanced analytics and artificial intelligence in gastrointestinal cancer: a systematic review of radiomics predicting response to treatment. Eur J Nucl Med Mol Imaging. 2021 Jun;48(6):1785-1794. doi: 10.1007/s00259-020-05142-w. Epub 2020 Dec 16. PMID: 33326049; PMCID: PMC8113210. (Without meta-analysis)
92. Wesdorp NJ, van Goor VJ, Kemna R, Jansma EP, van Waesberghe JHTM, Swijnenburg RJ, Punt CJA, Huiskens J, Kazemier G. Advanced image analytics predicting clinical outcomes in patients with colorectal liver metastases: A systematic review of the literature. Surg Oncol. 2021 Sep;38:101578. doi: 10.1016/j.suronc.2021.101578. Epub 2021 Apr 15. PMID: 33866191. (Without meta-analysis)
93. Won SY, Lee N, Park YW, Ahn SS, Ku CR, Kim EH, Lee SK. Quality reporting of radiomics analysis in pituitary adenomas: Promoting clinical translation. Br J Radiol. 2022 Aug 26:20220401. doi: 10.1259/bjr.20220401. Epub ahead of print. PMID: 36018049. (Without meta-analysis)
94. Xue C, Yuan J, Lo GG, Chang ATY, Poon DMC, Wong OL, Zhou Y, Chu WCW. Radiomics feature reliability assessed by intraclass correlation coefficient: a systematic review. Quant Imaging Med Surg. 2021 Oct;11(10):4431-4460. doi: 10.21037/qims-21-86. PMID: 34603997; PMCID: PMC8408801. (Radiomics methodology)
95. Zhang C, de A F Fonseca L, Shi Z, Zhu C, Dekker A, Bermejo I, Wee L. Systematic review of radiomic biomarkers for predicting immune checkpoint inhibitor treatment outcomes. Methods. 2021 Apr;188:61-72. doi: 10.1016/j.ymeth.2020.11.005. Epub 2020 Dec 1. PMID: 33271285. (Without meta-analysis)
96. Zheng X, He B, Hu Y, Ren M, Chen Z, Zhang Z, Ma J, Ouyang L, Chu H, Gao H, He W, Liu T, Li G. Diagnostic Accuracy of Deep Learning and Radiomics in Lung Cancer Staging: A Systematic Review and Meta-Analysis. Front Public Health. 2022 Jul 18;10:938113. doi: 10.3389/fpubh.2022.938113. PMID: 35923964; PMCID: PMC9339706. (Not radiomics)
97. Shrestha P, Poudyal B, Yadollahi S, E Wright D, V Gregory A, D Warner J, Korfiatis P, C Green I, L Rassier S, Mariani A, Kim B, K Laughlin-Tommaso S, L Kline T. A systematic review on the use of artificial intelligence in gynecologic imaging - Background, state of the art, and future directions. Gynecol Oncol. 2022 Jul 29:S0090-8258(22)00496-6. doi: 10.1016/j.ygyno.2022.07.024. Epub ahead of print. PMID: 35914978. (Without meta-analysis)
98. Fan Y, Feng M, Wang R. Application of Radiomics in Central Nervous System Diseases: a Systematic literature review. Clin Neurol Neurosurg. 2019 Dec;187:105565. doi: 10.1016/j.clineuro.2019.105565. Epub 2019 Oct 16. PMID: 31670024. (Without meta-analysis)
99. Sadaghiani MS, Rowe SP, Sheikhbahaei S. Applications of artificial intelligence in oncologic ^{18}F-FDG PET/CT imaging: a systematic review. Ann Transl Med. 2021 May;9(9):823. doi: 10.21037/atm-20-6162. PMID: 34268436; PMCID: PMC8246218. (Without meta-analysis)
100. Russo V, Lallo E, Munnia A, Spedicato M, Messerini L, D'Aurizio R, Ceroni EG, Brunelli G, Galvano A, Russo A, Landini I, Nobili S, Ceppi M, Bruzzone M, Cianchi F, Staderini F, Roselli M, Riondino S, Ferroni P, Guadagni F, Mini E, Peluso M. Artificial Intelligence Predictive Models of Response to Cytotoxic Chemotherapy Alone or Combined to Targeted Therapy for Metastatic Colorectal Cancer Patients: A Systematic Review and Meta-Analysis. Cancers (Basel). 2022 Aug 19;14(16):4012. doi: 10.3390/cancers14164012. PMID: 36011003; PMCID: PMC9406544. (Without meta-analysis)
101. Sollini M, Gelardi F, Matassa G, Delgado Bolton RC, Chiti A, Kirienko M. Interdisciplinarity: An essential requirement for translation of radiomics research into clinical practice -a systematic review focused on thoracic

oncology. Rev Esp Med Nucl Imagen Mol (Engl Ed). 2020 May-Jun;39(3):146-156. English, Spanish. doi: 10.1016/j.remn.2019.10.003. Epub 2020 Apr 8. PMID: 32278786. (Without meta-analysis)

102. Telecan T, Andras I, Crisan N, Giurgiu L, Căta ED, Caraiani C, Lebovici A, Boca B, Balint Z, Diosan L, Lupsor-Platon M. More than Meets the Eye: Using Textural Analysis and Artificial Intelligence as Decision Support Tools in Prostate Cancer Diagnosis-A Systematic Review. J Pers Med. 2022 Jun 16;12(6):983. doi: 10.3390/jpm12060983. PMID: 35743766; PMCID: PMC9225075. (Without meta-analysis)
103. Guerrisi A, Loi E, Ungania S, Russillo M, Bruzzaniti V, Elia F, Desiderio F, Marconi R, Solivetti FM, Strigari L. Novel cancer therapies for advanced cutaneous melanoma: The added value of radiomics in the decision making process-A systematic review. Cancer Med. 2020 Mar;9(5):1603-1612. doi: 10.1002/cam4.2709. Epub 2020 Jan 17. PMID: 31951322; PMCID: PMC7050080. (Not radiomics)
104. Bleker J, Kwee TC, Yakar D. Quality of Multicenter Studies Using MRI Radiomics for Diagnosing Clinically Significant Prostate Cancer: A Systematic Review. Life (Basel). 2022 Jun 23;12(7):946. doi: 10.3390/life12070946. PMID: 35888036; PMCID: PMC9324573. (Without meta-analysis)
105. Corr F, Grimm D, Saß B, Pojskić M, Bartsch JW, Carl B, Nimsky C, Bopp MHA. Radiogenomic Predictors of Recurrence in Glioblastoma-A Systematic Review. J Pers Med. 2022 Mar 4;12(3):402. doi: 10.3390/jpm12030402. PMID: 35330402; PMCID: PMC8952807. (Without meta-analysis)
106. Gherghe M, Lazar AM, Mutuleanu MD, Stanciu AE, Martin S. Radiomics Analysis of [^{18}F]FDG PET/CT Thyroid Incidentalomas: How Can It Improve Patients' Clinical Management? A Systematic Review from the Literature. Diagnostics (Basel). 2022 Feb 12;12(2):471. doi: 10.3390/diagnostics12020471. PMID: 35204561; PMCID: PMC8870948. (Without meta-analysis)
107. Lim EJ, Castellani D, So WZ, Fong KY, Li JQ, Tiong HY, Gadzhiev N, Heng CT, Teoh JY, Naik N, Ghani K, Sarica K, De La Rosette J, Somani B, Gauhar V. Radiomics in Urolithiasis: Systematic Review of Current Applications, Limitations, and Future Directions. J Clin Med. 2022 Aug 31;11(17):5151. doi: 10.3390/jcm11175151. PMID: 36079078. (Without meta-analysis)
108. Bicci E, Nardi C, Calamandrei L, Pietragalla M, Cavigli E, Mungai F, Bonasera L, Miele V. Role of Texture Analysis in Oropharyngeal Carcinoma: A Systematic Review of the Literature. Cancers (Basel). 2022 May 16;14(10):2445. doi: 10.3390/cancers14102445. PMID: 35626048; PMCID: PMC9139172. (Without meta-analysis)
109. Liberini V, Laudicella R, Capozza M, Huellner MW, Burger IA, Baldari S, Terreno E, Deandreis D. The Future of Cancer Diagnosis, Treatment and Surveillance: A Systemic Review on Immunotherapy and Immuno-PET Radiotracers. Molecules. 2021 Apr 11;26(8):2201. doi: 10.3390/molecules26082201. PMID: 33920423; PMCID: PMC8069316. (Not radiomics)
110. Alabi RO, Bello IO, Youssef O, Elmusrati M, Mäkitie AA, Almangush A. Utilizing Deep Machine Learning for Prognostication of Oral Squamous Cell Carcinoma-A Systematic Review. Front Oral Health. 2021 Jul 26;2:686863. doi: 10.3389/froh.2021.686863. PMID: 35048032; PMCID: PMC8757862. (Not radiomics)

List of included full-texts - in up-to-date search

1. Jia LL, Zheng QY, Tian JH, He DL, Zhao JX, Zhao LP, Huang G. Artificial intelligence with magnetic resonance imaging for prediction of pathological complete response to neoadjuvant chemoradiotherapy in rectal cancer: A systematic review and meta-analysis. Front Oncol. 2022 Oct 12;12:1026216. doi: 10.3389/fonc.2022.1026216. PMID: 36313696; PMCID: PMC9597310.

List of excluded full-texts (with justifications) - in up-to-date search

1. Guha A, Goda JS, Dasgupta A, Mahajan A, Halder S, Gawde J, Talole S. Classifying primary central nervous system lymphoma from glioblastoma using deep learning and radiomics based machine learning approach - a systematic review and meta-analysis. Front Oncol. 2022 Oct 3;12:884173. doi: 10.3389/fonc.2022.884173. PMID: 36263203; PMCID: PMC9574102. (Not radiomics)
2. O'Donnell JPM, Gasior SA, Davey MG, O'Malley E, Lowery AJ, McGarry J, O'Connell AM, Kerin MJ, McCarthy P. The accuracy of breast MRI radiomic methodologies in predicting pathological complete response to neoadjuvant chemotherapy: A systematic review and network meta-analysis. Eur J Radiol. 2022 Oct 17;157:110561. doi: 10.1016/j.ejrad.2022.110561. Epub ahead of print. PMID: 36308849. (Network meta-analysis focus on difference between models)

Supplementary Table S1 Data extraction sheet

Field	Item
Bibliographical Information	The Title of The Study
	The First Authors of The Study
	Published Year
	Journal Type
	Published Journal
	Impact Factor of Published Journal
	Published Volume
	Published Issue
	Published Page
	Study ID, determined by First Author + Year, and Journal, if needed
Study Characteristics	Participant characteristics
	Participant age
	Participant gender
	Imaging modality
	Inclusion criteria
	Exclusion criteria
	Restriction of language, publication period, publication type, etc.
	Information sources
	Number of Included Primary Studies
	Number of Cases and Controls
	Outcome
	Disease or anatomical site
	Oncological topic
	Quality assessment tool
	Results of quality assessment
	Rating of quality assessment tool
	Risk of bias assessment tools
	Rating of risk of bias assessment tools
	Supplementary materials

	Protocols
	Funding sources
Effect Metrics at Systematic Review Level	Number of Events and Sample Size
	Pooled Sensitivity
	Pooled Specificity
	Pooled Accuracy
	Pooled Positive Predictive Value (PPV)
	Pooled Negative Predictive Value (NPV)
	Pooled Positive Likelihood Ratio (PLR)
	Pooled Negative Likelihood Ratio (NLR)
	Pooled Diagnostic Odds Ratio (DOR)
Effect Metrics at Individual Study Level	Number of Events and Sample Size
	Pooled Sensitivity
	Pooled Specificity
	Pooled Accuracy
	Pooled Positive Predictive Value (PPV)
	Pooled Negative Predictive Value (NPV)
	Pooled Positive Likelihood Ratio (PLR)
	Pooled Negative Likelihood Ratio (NLR)
	Pooled Diagnostic Odds Ratio (DOR)

Supplementary Table S2 PRISMA 2020 abstract checklist for reporting quality assessment

Section and Topic	Item #	Checklist item
**TITLE**		
Title	1	Identify the report as a systematic review.
**BACKGROUND**		
Objectives	2	Provide an explicit statement of the main objective(s) or question(s) the review addresses.
**METHODS**		
Eligibility criteria	3	Specify the inclusion and exclusion criteria for the review
Information sources	4	Specify the information sources (e.g., databases, registers) used to identify studies and the date when each was last searched.
Risk of bias	5	Specify the methods used to assess the risk of bias in the included studies.
Synthesis	6	Specify the methods used to present and synthesize results.
**RESULTS**		
Inckuded studies	7	Give the total number of included studies and participants and summarise relevant characteristics of studies.
Synthesis of results	8	Present results for main outcomes, preferably indicating the number of included studies and participants for each<br>If meta-analysis was done, report the summary estimate and confidence/credible interval. If comparing groups, indicate the direction of the effect (i.e., which group is favored).
**DISCUSSION**		
Limitations of evidence	9	Provide a brief summary of the limitations of the evidence included in the review (e.g., study risk of bias, inconsistency, and imprecision).
Interpretation	10	Provide a general interpretation of the results and important implications.
**OTHER**		
Funding	11	Specify the primary source of funding for the review.
Registration	12	Provide the register name and registration number

Note: This checklist is extracted from: Page MJ, McKenzie JE, Bossuyt PM, Boutron I, Hoffmann TC, Mulrow CD, Shamseer L, Tetzlaff JM, Akl EA, Brennan SE, Chou R, Glanville J, Grimshaw JM, Hróbjartsson A, Lalu MM, Li T, Loder EW, Mayo-Wilson E, McDonald S, McGuinness LA, Stewart LA, Thomas J, Tricco AC, Welch VA, Whiting P, Moher D. The PRISMA 2020 statement: an updated guideline for reporting systematic reviews. BMJ. 2021 Mar 29;372:n71. doi: 10.1136/bmj.n71. PMID: 33782057; PMCID: PMC8005924.

Supplementary Table S3 PRISMA 2020 checklist for reporting quality assessment

Section and Topic	Item #	Checklist item
**TITLE**		
Title	1	Identify the report as a systematic review. (Also see the PRISMA 2020 for Abstracts checklist.)
**ABSTRACT**		
Abstract	2	See the PRISMA 2020 for Abstracts checklist.
**INTRODUCTION**		
Rationale	3	Describe the rationale for the review in the context of existing knowledge.
Objectives	4	Provide an explicit statement of the objective(s) or question(s) the review addresses.
**METHODS**		
Eligibility criteria	5	Specify the inclusion and exclusion criteria for the review and how studies were grouped for the syntheses.
Information sources	6	Specify all databases, registers, websites, organisations, reference lists and other sources searched or consulted to identify studies. Specify the date when each source was last searched or consulted.
Search strategy	7	Present the full search strategies for all databases, registers and websites, including any filters and limits used.
Selection process	8	Specify the methods used to decide whether a study met the inclusion criteria of the review, including how many reviewers screened each record and each report retrieved, whether they worked independently, and if applicable, details of automation tools used in the process.
Data collection process	9	Specify the methods used to collect data from reports, including how many reviewers collected data from each report, whether they worked independently, any processes for obtaining or confirming data from study investigators, and if applicable, details of automation tools used in the process.
Data items	10a	List and define all outcomes for which data were sought. Specify whether all results that were compatible with each outcome domain in each study were sought (e.g. for all measures, time points, analyses), and if not, the methods used to decide which results to collect.
	10b	List and define all other variables for which data were sought (e.g. participant and intervention characteristics, funding sources). Describe any assumptions made about any missing or unclear information.
Study risk of bias assessment	11	Specify the methods used to assess risk of bias in the included studies, including details of the tool(s) used, how many reviewers assessed each study and whether they worked independently, and if applicable, details of automation tools used in the process.
Effect measures	12	Specify for each outcome the effect measure(s) (e.g. risk ratio, mean difference) used in the synthesis or presentation of results.

Synthesis methods	13a	Describe the processes used to decide which studies were eligible for each synthesis (e.g. tabulating the study intervention characteristics and comparing against the planned groups for each synthesis (item #5)).
	13b	Describe any methods required to prepare the data for presentation or synthesis, such as handling of missing summary statistics, or data conversions.
	13c	Describe any methods used to tabulate or visually display results of individual studies and syntheses.
	13d	Describe any methods used to synthesize results and provide a rationale for the choice(s). If meta-analysis was performed, describe the model(s), method(s) to identify the presence and extent of statistical heterogeneity, and software package(s) used.
	13e	Describe any methods used to explore possible causes of heterogeneity among study results (e.g. subgroup analysis, meta-regression).
	13f	Describe any sensitivity analyses conducted to assess robustness of the synthesized results.
Reporting bias assessment	14	Describe any methods used to assess risk of bias due to missing results in a synthesis (arising from reporting biases).
Certainty assessment	15	Describe any methods used to assess certainty (or confidence) in the body of evidence for an outcome.
**RESULTS**		
Study selection	16a	Describe the results of the search and selection process, from the number of records identified in the search to the number of studies included in the review, ideally using a flow diagram.
	16b	Cite studies that might appear to meet the inclusion criteria, but which were excluded, and explain why they were excluded.
Study characteristics	17	Cite each included study and present its characteristics.
Risk of bias in studies	18	Present assessments of risk of bias for each included study.
Results of individual studies	19	For all outcomes, present, for each study: (a) summary statistics for each group (where appropriate) and (b) an effect estimate and its precision (e.g. confidence/credible interval), ideally using structured tables or plots.
Results of syntheses	20a	For each synthesis, briefly summarise the characteristics and risk of bias among contributing studies.
	20b	Present results of all statistical syntheses conducted. If meta-analysis was done, present for each the summary estimate and its precision (e.g. confidence/credible interval) and measures of statistical heterogeneity. If comparing groups, describe the direction of the effect.
	20c	Present results of all investigations of possible causes of heterogeneity among study results.
	20d	Present results of all sensitivity analyses conducted to assess the robustness of the synthesized results.
Reporting biases	21	Present assessments of risk of bias due to missing results (arising from reporting biases) for each synthesis assessed.
Certainty of evidence	22	Present assessments of certainty (or confidence) in the body of evidence for each outcome assessed.

**DISCUSSION**		
Discussion	23a	Provide a general interpretation of the results in the context of other evidence.
	23b	Discuss any limitations of the evidence included in the review.
	23c	Discuss any limitations of the review processes used.
	23d	Discuss implications of the results for practice, policy, and future research.
**OTHER INFORMATION**		
Registration and protocol	24a	Provide registration information for the review, including register name and registration number, or state that the review was not registered.
	24b	Indicate where the review protocol can be accessed, or state that a protocol was not prepared.
	24c	Describe and explain any amendments to information provided at registration or in the protocol.
Support	25	Describe sources of financial or non-financial support for the review, and the role of the funders or sponsors in the review.
Competing interests	26	Declare any competing interests of review authors.
Availability of data, code and other materials	27	Report which of the following are publicly available and where they can be found: template data collection forms; data extracted from included studies; data used for all analyses; analytic code; any other materials used in the review.

Note: This checklist is extracted from: Page MJ, McKenzie JE, Bossuyt PM, Boutron I, Hoffmann TC, Mulrow CD, Shamseer L, Tetzlaff JM, Akl EA, Brennan SE, Chou R, Glanville J, Grimshaw JM, Hróbjartsson A, Lalu MM, Li T, Loder EW, Mayo-Wilson E, McDonald S, McGuinness LA, Stewart LA, Thomas J, Tricco AC, Welch VA, Whiting P, Moher D. The PRISMA 2020 statement: an updated guideline for reporting systematic reviews. BMJ. 2021 Mar 29;372:n71. doi: 10.1136/bmj.n71. PMID: 33782057; PMCID: PMC8005924.

Supplementary Table S4 AMSTAR-2 tool for methodological quality assessment

No	Question	Partial Yes	Yes
1	Did the research questions and inclusion criteria for the review include the components of PICO?	n/a	The research questions and inclusion criteria for the review should include Population, Intervention, Comparator group, and Outcome. Timeframe for follow-up is optional (recommended) to get a yes.
2*	Did the report of the review contain an explicit statement that the review methods were established prior to the conduct of the review and did the report justify any significant deviations from the protocol?	For Partial Yes, the authors state that they had a written protocol or guide that included ALL the following: review question(s), a search strategy, inclusion/exclusion criteria, a risk of bias assessment.	As for partial yes, plus the protocol should be registered and should also have specified: a meta-analysis/synthesis plan, if appropriate, and a plan for investigating causes of heterogeneity, justification for any deviations from the protocol.
3	Did the review authors explain their selection of the study designs for inclusion in the review?	n/a	For Yes, the review should satisfy ONE of the following: explanation for including only RCTs, OR explanation for including only NRSI, OR explanation for including both RCTs and NRSI.
4*	Did the review authors use a comprehensive literature search strategy?	For Partial Yes, searched at least 2 databases (relevant to research question), provided key word and/or search strategy, justified publication restrictions (eg, language).	For Yes, should also have (all the following): searched the reference lists/bibliographies of included studies, searched trial/study registries, included/consulted content experts in the field, where relevant, searched for grey literature, conducted search within 24 months of completion of the review.
5	Did the review authors perform study selection in duplicate?	n/a	For Yes, either ONE of the following: at least two reviewers independently agreed on selection of eligible studies and achieved consensus on which studies to include, OR two reviewers selected a sample of eligible studies and achieved good agreement (at least 80 per cent), with the remainder selected by one reviewer.
6	Did the review authors perform data extraction in duplicate?	n/a	For Yes, either ONE of the following: at least two reviewers achieved consensus on which data to extract from included studies, OR two reviewers extracted data from a sample of

			eligible studies and achieved good agreement (at least 80 per cent), with the remainder extracted by one reviewer.
7*	Did the review authors provide a list of excluded studies and justify the exclusions?	For Partial Yes, provided a list of all potentially relevant studies that were read in full text form but excluded from the review.	For Yes, must also have: justified the exclusion from the review of each potentially relevant study.
8	Did the review authors describe the included studies in adequate detail?	For Partial Yes, (ALL the following): described populations, described interventions, described comparators, described outcomes, described research designs.	For Yes, should also have ALL the following: described population in detail, described intervention and comparator in detail (including doses where relevant), described study's setting, timeframe for follow-up.
9*	Did the review authors use a satisfactory technique for assessing the risk of bias (RoB) in individual studies that were included in the review?	RCTs: For Partial Yes, must have assessed RoB from unconcealed allocation, and lack of blinding of patients and assessors when assessing outcomes (unnecessary for objective outcomes such as all-cause mortality).	RCTs: For Yes, must also have assessed RoB from: allocation sequence that was not truly random, and selection of the reported result from among multiple measurements or analyses of a specified outcome.
		NRSI: For Partial Yes, must have assessed RoB: from confounding, and from selection bias.	NRSI: For Yes, must also have assessed RoB: methods used to ascertain exposures and outcomes, and selection of the reported result from among multiple measurements or analyses of a specified outcome.
10	Did the review authors report on the sources of funding for the studies included in the review?	n/a	For Yes, must have reported on the sources of funding for individual studies included in the review.<br>Note: Reporting that the reviewers looked for this information but it was not reported by study authors also qualifies.
11*	If meta-analysis was performed did the review authors use appropriate methods for statistical combination of results?	n/a	RCTs: For Yes, the authors justified combining the data in a meta-analysis, AND they used an appropriate weighted technique to combine study results and adjusted for heterogeneity if present, AND investigated the causes of any heterogeneity.
		n/a	NRSI: For Yes: the authors justified combining the data in a meta-analysis, AND they used an appropriate weighted technique to

			combine study results, adjusting for heterogeneity if present, AND they statistically combined effect estimates from NRSI that were adjusted for confounding, rather than combining raw data, or justified combining raw data when adjusted effect estimates were not available, AND they reported separate summary estimates for RCTs and NRSI separately when both were included in the review.
12	If meta-analysis was performed, did the review authors assess the potential impact of RoB in individual studies on the results of the meta-analysis or other evidence synthesis?	n/a	For Yes, included only low risk of bias RCTs, OR, if the pooled estimate was based on RCTs and/or NRSI at variable RoB, the authors performed analyses to investigate possible impact of RoB on summary estimates of effect.
13*	Did the review authors account for RoB in individual studies when interpreting/discussing the results of the review?	n/a	For Yes, included only low risk of bias RCTs, OR, if RCTs with moderate or high RoB, or NRSI were included the review provided a discussion of the likely impact of RoB on the results.
14	Did the review authors provide a satisfactory explanation for, and discussion of, any heterogeneity observed in the results of the review?	n/a	For Yes, there was no significant heterogeneity in the results, OR if heterogeneity was present the authors performed an investigation of sources of any heterogeneity in the results and discussed the impact of this on the results of the review.
15*	If they performed quantitative synthesis did the review authors carry out an adequate investigation of publication bias (small study bias) and discuss its likely impact on the results of the review?	n/a	For Yes, performed graphical or statistical tests for publication bias and discussed the likelihood and magnitude of impact of publication bias.
16	Did the review authors report any potential sources of conflict of interest, including any funding they received for conducting the review?	n/a	For Yes, the authors reported no competing interests, OR the authors described their funding sources and how they managed potential conflicts of interest.
**Rating overall confidence**		• High: No or one non-critical weakness: the systematic review provides an accurate and comprehensive summary of the results of the available studies that address the question of interest.	

	• Moderate: More than one non-critical weakness**: the systematic review has more than one weakness but no critical flaws. It may provide an accurate summary of the results of the available studies that were included in the review.<br>• Low: One critical flaw with or without non-critical weaknesses: the review has a critical flaw and may not provide an accurate and comprehensive summary of the available studies that address the question of interest.<br>• Critically low: More than one critical flaw with or without non-critical weaknesses: the review has more than one critical flaw and should not be relied on to provide an accurate and comprehensive summary of the available studies.<br>* critical items.<br>** Multiple non-critical weaknesses may diminish confidence in the review and it may be appropriate to move the overall appraisal down from moderate to low confidence.

Note: This checklist is extracted from Shea BJ, Reeves BC, Wells G, Thuku M, Hamel C, Moran J, Moher D, Tugwell P, Welch V, Kristjansson E, Henry DA. AMSTAR 2: a critical appraisal tool for systematic reviews that include randomised or non-randomised studies of healthcare interventions, or both. BMJ. 2017 Sep 21;358:j4008. doi: 10.1136/bmj.j4008. PMID: 28935701; PMCID: PMC5833365.

Supplementary Table S5 ROBIS tool for risk of bias assessment

	Phase 2				Phase 3
	Domain 1: Study eligibility criteria	Domain 2 Identification and selection of studies	Domain 3: Data collection and study appraisal	Domain 4: Synthesis and findings	
**Signaling Questions**	1.1 Did the review adhere to pre-defined objectives and eligibility criteria?<br>1.2 Were the eligibility criteria appropriate for the review question?<br>1.3 Were eligibility criteria unambiguous?<br>1.4 Were all restrictions in eligibility criteria based on study characteristics appropriate?<br>1.5 Were any restrictions in eligibility criteria based on sources of information appropriate?	2.1 Did the search include an appropriate range of databases/ electronic sources for published and unpublished reports?<br>2.2 Were methods additional to database searching used to identify relevant reports?<br>2.3 Were the terms and structure of the search strategy likely to retrieve as many eligible studies as possible?<br>2.4 Were restrictions based on date, publication format, or language appropriate?<br>2.5 Were efforts made to minimise errors in selection of studies?	3.1 Were efforts made to minimise error in data collection?<br>3.2 Were sufficient study characteristics available for both review authors and readers to be able to interpret the results?<br>3.3 Were all relevant study results collected for use in the synthesis?<br>3.4 Was risk of bias (or methodological quality) formally assessed using appropriate criteria?<br>3.5 Were efforts made to minimise error in risk of bias assessment?<br>Concerns regarding methods used to collect data and appraise studies	4.1 Did the synthesis include all studies that it should?<br>4.2 Were all predefined analyses followed or departures explained?<br>4.3 Was the synthesis appropriate given the nature and similarity in the research questions, study designs and outcomes across included studies?<br>4.4 Was between-studies variation (heterogeneity) minimal or addressed in the synthesis?<br>4.5 Was robustness of the finding(s) assessed e.g. through funnel plot or sensitivity analyses?<br>4.6 Were biases in primary studies minimal or addressed in the synthesis?	A. Did the interpretation of findings address all of the concerns identified the Phase 2 assessment?<br>B. Was the relevance of identified studies to the review's research question appropriately considered?<br>C. Did the reviewers avoid emphasizing results on the basis of their statistical significance?
**Judgement**	Concerns regarding specification of study eligibility criteria	Concerns regarding methods used to identify and/or select studies	Concerns regarding methods used to collect data and appraise studies	Concerns regarding methods used to synthesize results	Risk of bias introduced by methods used to identify and/or select studies

Note: This checklist is extracted from Whiting P, Savović J, Higgins JP, Caldwell DM, Reeves BC, Shea B, Davies P, Kleijnen J, Churchill R; ROBIS group. ROBIS: A new tool to assess risk of bias in systematic reviews was developed. J Clin Epidemiol. 2016 Jan;69:225-34. doi: 10.1016/j.jclinepi.2015.06.005. Epub 2015 Jun 16. PMID: 26092286; PMCID: PMC4687950.

Supplementary Table S6 Category of five levels of evidence based on meta-analyzes

**Levels of supporting evidence**	**Description**
Convincing	$p < 10^{-6}$, > 1000 events, the largest study reaches statistical significance ($p < 0.05$), $I2 < 50\%$, the null value excluded by the 95% PI, no small-study effects ($p > 0.1$ for Egger's test) and excess significance ($p > 0.1$).
Highly suggestive	$p < 10^{-6}$, > 1000 events, the largest study reaches statistical significance ($p < 0.05$)
Suggestive	$p < 10^{-3}$, > 1000 events
Weak	$p < 0.05$
Not suggestive	$p > 0.05$

Note: Fusar-Poli P, Radua J. Ten simple rules for conducting umbrella reviews. Evid Based Ment Health. 2018 Aug;21(3):95-100. doi: 10.1136/ebmental-2018-300014. Epub 2018 Jul 13. PMID: 30006442.

Supplementary Table S7 Bibliographic information of included systematic reviews

Study ID	Author	Year	Journal	Impact factor	JCR quartile	Journal type	First authorship	Biomarker	Disease or anatomical site	Oncology
Bedrikovetski2021A	Bedrikovetski	2021	BMC Cancer	4.638	Q2	Non-imaging	Non-radiologist	Diag	Rectal Colonal	Yes
Bedrikovetski2021B	Bedrikovetski	2021	Artif Intell Med	7.011	Q1	Non-imaging	Non-radiologist	Diag	Abdominopelvic malignancy	Yes
Bhandari2021	Bhandari	2021	AJNR Am J Neuroradiol	4.966	Q2	Imaging	Non-radiologist	Diag	Glioma	Yes
Cao2022	Cao	2022	Chin J Med Imaging Technol	n. a.	n. a.	Imaging	Radiologist	Diag	Renal Cell Carcinoma	Yes
Castaldo2021	Castaldo	2021	J Med Internet Res	7.093	Q1	Non-imaging	Non-radiologist	Diag	Prostate	Yes
Chen2021	Chen	2021	Eur J Nucl Med Mol Imaging	10.057	Q1	Imaging	Radiologist	Pre/Pro	NSCLC	Yes
Cleere2022	Cleere	2022	Diagnostics (Basel)	3.992	Q2	Non-imaging	Non-radiologist	Diag	Thyroid	Yes
Davey2021A	Davey	2021	Colorectal Dis	3.917	Q1	Non-imaging	Non-radiologist	Pre/Pro	Rectal	Yes
Davey2021B	Davey	2021	BJS Open	3.875	Q1	Non-imaging	Non-radiologist	Pre/Pro	Breast	Yes
Davey2021C	Davey	2021	Eur J Radiol	4.531	Q2	Imaging	Non-radiologist	Diag	Breast	Yes
Deantonio2022	Deantonio	2022	Front Oncol	5.738	Q2	Non-imaging	Non-radiologist	Pre/Pro	Esophageal	Yes
Gao2022A	Gao	2022	Eur Radiol	7.034	Q1	Imaging	Radiologist	Pre/Pro	Pancreatic cancer	Yes

Gao2022B	Gao	2022	Chinese Journal of Evidence-Based Medicine	n. a.	n. a.	Non-imaging	Radiologist	Diag	Glioblastoma	Yes
Han2022	Han	2022	Eur Radiol	7.034	Q1	Imaging	Radiologist	Diag	Glioma	Yes
Huang2020	Huang	2020	Front Oncol	5.738	Q2	Non-imaging	Non-radiologist	Diag	Liver	Yes
Huang2021	Huang	2021	Diagn Interv Radiol	3.346	Q3	Imaging	Radiologist	Diag	Glioma	Yes
Jia2022	Jia	2022	Front Oncol	5.738	Q2	Non-imaging	Non-radiologist	Pre/Pro	Rectal	Yes
Kao2021A	Kao	2021	In Vivo	2.406	Q4	Non-imaging	Non-radiologist	Pre/Pro	Esophageal	Yes
Kao2021B	Kao	2021	Diagnostics (Basel)	3.992	Q2	Non-imaging	Non-radiologist	Diag	COVID-19	No
Kao2022	Kao	2022	Radiol Med	6.313	Q1	Imaging	Non-radiologist	Pre/Pro	COVID-19	No
Kothari2021	Kothari	2021	Radiother Oncol	6.901	Q1	Imaging	Non-radiologist	Pre/Pro	NSCLC	Yes
Kozikowski2021	Kozikowski	2021	Eur Urol Focus	5.952	Q1	Non-imaging	Non-radiologist	Diag	Bladder Cancer	Yes
Lee2022	Lee	2022	Cancers (Basel)	6.575	Q1	Non-imaging	Radiologist	Pre/Pro	Nasopharyngeal carcinoma	Yes
Li2022A	Li	2021	Front Oncol	5.738	Q2	Non-imaging	Radiologist	Diag	Liver	Yes
Li2022B	Li	2021	Eur J Radiol	4.531	Q2	Imaging	Radiologist	Diag	Cervical cancer	Yes
Li2022C	Li	2021	Eur Radiol	7.034	Q1	Imaging	Non-radiologist	Diag	Glioma	Yes
Li2022D	Li	2021	Front Oncol	5.738	Q2	Non-imaging	Radiologist	Diag	Breast	Yes
Liang2022	Liang	2022	Eur J Radiol	4.531	Q2	Imaging	Radiologist	Pre/Pro	Breast	Yes

Mühlbauer2021	Mühlbauer	2021	Cancers (Basel)	6.575	Q1	Non-imaging	Non-radiologist	Diag	Renal Cell Carcinoma	Yes
Pesapane2022	Pesapane	2022	Curr Probl Cancer	2.367	Q4	Non-imaging	Radiologist	Pre/Pro	Breast	Yes
Ren2022	Ren	2022	Eur J Radiol	4.531	Q2	Imaging	Radiologist	Diag	Cervical cancer	Yes
Sha2022	Sha	2022	Clin Radiol	3.389	Q2	Imaging	Radiologist	Diag	Breast	Yes
Sohn2020	Sohn	2020	Contrast Media Mol Imaging	3.009	Q3	Imaging	Non-radiologist	Diag	Glioma	Yes
Ugga2021	Ugga	2021	Neuroradiology	2.995	Q3	Imaging	Non-radiologist	Diag and Pre/Pro	Meningioma	Yes
Ursprung2020	Ursprung	2020	Eur Radiol	7.034	Q1	Imaging	Radiologist	Diag and Pre/Pro	Renal cell carcinoma	Yes
Yang2022	Yang	2022	Front Oncol	5.738	Q2	Non-imaging	Non-radiologist	Pre/Pro	Nasopharyngeal carcinoma	Yes
Zhang2022A	Zhang	2022	Front Oncol	5.738	Q2	Non-imaging	Non-radiologist	Diag	Liver	Yes
Zhang2022B	Zhang	2022	Front Oncol	5.738	Q2	Non-imaging	Radiologist	Diag	Breast	Yes
Zhang2022C	Zhang	2022	Front Oncol	5.738	Q2	Non-imaging	Non-radiologist	Diag	Adrenal mass	Yes
Zhong2021	Zhong	2021	Eur Radiol	7.034	Q1	Imaging	Radiologist	Diag and Pre/Pro	Osteosarcoma	Yes
Zhong2022A	Zhong	2022	Eur Radiol	7.034	Q1	Imaging	Radiologist	Diag and Pre/Pro	Chondrosarcoma	Yes
Zhong2022B	Zhong	2022	Insights Imaging	5.036	Q1	Imaging	Radiologist	Diag and Pre/Pro	Pancreatitis	No
Zhong2022C	Zhong	2022	Insights Imaging	5.036	Q1	Imaging	Radiologist	Diag and Pre/Pro	Osteosarcoma	Yes
Zhong2022D	Zhong	2022	Abdom Radiol (NY)	2.886	Q3	Imaging	Radiologist	Diag	Liver	Yes

Note: Impact factor, JCR quartile, and Journal type are extracted according to Journal Citation Reports 2021, which became available on Jun 2022 (https://access.clarivate.com/).

Supplementary Table S8 Review topics of included systematic reviews

Study ID	No. of primary studies	Study aim	Key conclusion
Bedrikovetski2021A	12	To evaluate the diagnostic accuracy of artificial intelligence (AI) models used for detection of lymph node metastasis on pre-operative staging imaging for colorectal cancer.	AI models have the potential to predict lymph node metastasis more accurately in rectal and colorectal cancer, however, radiomics studies are heterogeneous and deep learning studies are scarce.
Bedrikovetski2021B	20	To assesses the diagnostic performance of deep learning algorithms and radiomics models for lymph node metastases in abdominopelvic malignancies.	Radiomics models improve the diagnostic accuracy of lymph node staging for abdominopelvic malignancies in comparison with radiologist's assessment. Deep learning models may further improve on this, but data remain limited.
Bhandari2021	14	(1) To perform a diagnostic test accuracy systematic review for classifying IDH and 1p19q status using MR imaging radiomics. (2) To provide future directions for integration into clinical radiology.	Radiogenomics is a potential alternative to standard invasive biopsy techniques for determination of IDH and 1p19q status in lower-grade gliomas but requires translational research for clinical uptake.
Cao2022	16	To observe the value of CT radiomics for predicting pathological grade of clear cell renal carcinoma (cRCC) with meta-analysis.	CT radiomics was effective for predicting pathological grade of cRCC.
Castaldo2021	37	(1) To assesses the source of heterogeneity and the performance of machine learning applied to radiomic, genomic, and clinical biomarkers for the diagnosis of prostate cancer. (2) To clearly identify problems and issues related to the implementation of machine learning in clinical studies.	The performance of machine learning for diagnosis of prostate cancer was considered satisfactory for several studies investigating the multiparametric magnetic resonance imaging and urine biomarkers; however, given the limitations indicated in our study, further studies are warranted to extend the potential use of machine learning to clinical settings. Recommendations on the use of machine learning techniques were also provided to help researchers to design robust studies to facilitate evidence generation from the use of radiomic and genomic biomarkers.
Chen2021	15	To systematically evaluate the methodological quality of radiomic studies for predicting immunotherapy response or outcome in patients with non-small cell lung cancer (NSCLC).	Radiomics has potential to noninvasively predict immunotherapy response and outcome in patients with NSCLC. However, it has not yet been implemented as a clinical decision-making tool. Further

			external validation and evaluation within clinical pathway can facilitate personalized treatment for patients with NSCLC.
Cleere2022	75	To evaluate the diagnostic utility of radiomics in classifying undetermined thyroid nodules into benign and malignant using ultrasonography (US).	Radiomic analysis using US provides a reproducible, reliable evaluation of undetermined thyroid nodules when compared to current best practice.
Davey2021A	7	To perform a systematic review of the current literature evaluating the use of radiomics in predicting distant recurrence (DR) in patients with resected rectal carcinoma (RC).	This systematic review suggests the benefit of radiomic analysis of preoperative MRI in identifying patients with resected RC at an increased risk of DR. Our findings warrant validation in larger prospective studies as modalities to predict DR is a significant unmet need in RC. Radiomics may allow for tailored therapeutic strategies for high-risk groups.
Davey2021B	9	To perform a systematic review of current evidence evaluating the comparability of radiomics and Oncotype DXVR recurrence score (RS).	Radiomic tumour analysis is comparable to RS in differentiating patients into clinically relevant subgroups. For patients requiring MRI, radiomics may complement and enhance RS for prognostication and therapeutic decision making in ER+ breast cancer.
Davey2021C	41	To perform a systematic review of the current literature to evaluate the value radiomics in differentiating breast cancers into their molecular subtypes using diagnostic imaging.	Radiomic tumour assessment of contemporary breast imaging provide a novel option in determining breast cancer molecular subtypes. However, amelioration of such techniques are required and genetic expression assessment will remain the gold standard.
Deantonio2022	5	To provide current evidence of 18F-FDG PET-based radiomics in predicting response treatments following neoadjuvant chemoradiotherapy in oesophageal cancer.	Radiomics models exhibited a good performance in predicting pathological complete responses (pCRs). This review further strengthens the great potential of 18F- FDG PET-based radiomics to predict pCRs in oesophageal cancer patients who underwent neoadjuvant chemoradiotherapy. Additionally, our review imparts additional support to prospective studies on 18F-FDG PET radiomics for a tailored treatment strategy of oesophageal cancer patients.

Gao2022A	23	To systematically evaluate the prognostic prediction accuracy of radiomics features extracted from pre-treatment imaging in patients with pancreatic ductal adenocarcinoma (PDAC).	First-order entropy was significantly associated with overall survival (OS) and might improve the accuracy of PDAC prognosis prediction. Existing studies were poorly validated, and it should be noted in future studies. Modification of PROBAST for radiomics studies is necessary since the strict requirements of prospective study design may not be applicable to the demand for a large sample size in the model construction stage.
Gao2022B	37	To systematically review the value of radiomics in the diagnosis of glioblastoma.	The current evidence shows that radiomics provides good diagnostic accuracy for glioblastoma. Due to the limited quality and quantity of the included studies, more high-quality studies are required to verify the above conclusions.
Han2022	14	To reveal a radiogenomic correlation between the presence of the T2-fluid-attenuated inversion recovery resection (T2-FLAIR) mismatch sign on MR images and isocitrate dehydrogenase (IDH) mutation status in adult patients with lower-grade gliomas (LGGs).	The T2-FLAIR mismatch sign was an insensitive but highly specific marker for IDHmut-Noncodel and IDH-Mutation LGGs, whereas it was not a useful marker for IDHmut-Codel LGGs. The findings might identify the T2-FLAIR mismatch sign as a non-invasive imaging biomarker for the selection of patients with IDH-mutant LGGs.
Huang2020	9	To compare the predictive power between radiomics and non-radiomics (conventional imaging and functional imaging methods) for preoperative evaluation of microvascular invasion (MVI) in hepatocellular carcinoma (HCC).	The imaging method is feasible to predict the MVI state of HCC. Radiomics method based on medical image data is a promising application in clinical practice and can provide quantifiable image features. With the help of these features, highly consistent prediction performance will be achieved in anticipation.
Huang2021	15	To assess the diagnostic performance of radiomics using machine learning algorithms to predict the methylation status of the O6-methylguanine-DNA methyltransferase (MGMT) promoter in glioma patients.	This meta-analysis demonstrated that machine learning is a promising, reliable and repeatable candidate method for predicting MGMT promoter methylation status in glioma and showed a higher performance than non-machine learning methods.
Jia2022	16	(1) To evaluate the diagnostic accuracy of artificial intelligence (AI) models with MRI in predicting pathological complete response(pCR) to neoadjuvant chemoradiotherapy (nCRT) in patients with rectal cancer. (2) To assessed the methodological quality of the models.	Radiomics is a promising noninvasive method with high value in predicting pathological response to nCRT in patients with rectal cancer. DL models have higher predictive accuracy than radiomics models, and combined models incorporating clinical factors have

			higher diagnostic accuracy than radiomics models alone. In the future, prospective, large-scale, multicenter investigations using radiomics approaches will strengthen the diagnostic power of pCR.
Kao2021A	10	To investigate the predictive power of radiomics in esophageal cancer.	Using radiomics to predict complete pathological response after neoadjuvant chemoradiotherapy in esophageal cancer is feasible. In the future, prospective, multicenter studies should be carried out for predicting pathological complete response in patients with esophageal cancer
Kao2021B	7	To use computerized tomography (CT)-based radiomics models to differentiate COVID-19 pneumonia from other viral pneumonia infections.	Our meta-analysis showed that CT-based radiomics feature models can successfully differentiate COVID-19 from other viral pneumonias.
Kao2022	8	To investigate the predictive power of a CT-based radiomics model in determining COVID-19 severity.	This meta-analysis demonstrated that CT-based radiomics models might be helpful for predicting the severity of COVID-19 pneumonia.
Kothari2021	40	To perform a systematic review and meta-analysis of the prognostic value of radiomics models in patients with non-small cell lung cancer (NSCLC) treated with curative intent radiotherapy.	Based on this review, radiomics based models for lung cancer have to date demonstrated modest prognostic capabilities. Future research should consider using standardized radiomics features, robust feature selection and model development, and deep learning techniques, absolving the need for pre-defined features, to improve imaging-based models.
Kozikowski2021	8	To systematically review the diagnostic performance of radiomic techniques in predicting muscle-invasive bladder cancer (MIBC).	Radiomics shows high diagnostic performance in predicting MIBC. Despite differences in approaches, radiomic models were relatively homogeneous in their diagnostic accuracy. With further improvements, radiomics has the potential to become a useful adjunct in clinical management of bladder cancer.
Lee2022	10	To comprehensively assess the prognostic value of MRI-based radiomics for untreated nasopharyngeal carcinoma (NPC).	MRI-based radiomics shows good prognostic performance in predicting the PFS of patients with untreated NPC. However, more consistent and robust study protocols are necessary to validate the prognostic role of radiomics for NPC.

Li2022A	22	To perform a meta-analysis to investigate the diagnostic performance of radiomics for the preoperative evaluation of MVI in HCC and the effect of potential factors.	Radiomics is a promising noninvasive method that has high preoperative diagnostic performance for MVI status. Radiomics based on CT and MRI had a comparable predictive performance for MVI in HCC. Prospective, large-scale and multicenter studies with radiomics methods will improve the diagnostic power for MVI in the future.
Li2022B	12	To evaluate the ability of preoperative MRI-based radiomic features in predicting lymph node metastasis (LNM) in patients with cervical cancer.	Our meta-analysis showed that preoperative MRI-based radiomic features performs well in predicting LNM in patients with cervical cancer. This noninvasive and convenient tool may be used to facilitate preoperative identification of LNM.
Li2022C	17	(1) To evaluate the diagnostic performance of radiomics in differentiating high-grade glioma from brain metastasis and how to improve the model. (2) To assess the methodological quality of radiomics studies and explore ways of embracing the clinical application of radiomics.	Radiomics can accurately differentiate high-grade glioma from brain metastasis. The adoption of standardized workflow to avoid potential data leakage as well as the integration of clinical features and radiomics are advised to consider in future studies.
Li2022D	19	To evaluate the preoperative predictive value of radiomics in the diagnosis of breast cancer (BC).	Radiomics has shown excellent diagnostic performance in the preoperative prediction of BC and is expected to be a promising method in clinical practice.
Liang2022	15	To determine the diagnostic accuracy of machine learning (ML) models with MRI in predicting pathological response to neoadjuvant chemotherapy in patients with breast cancer. Furthermore, we compared the pathologic complete response (pCR) prediction performance of ML + radiomics with that of a deep learning (DL) algorithm.	ML applied to MRI enabled moderate accuracy in predicting pathological response to neoadjuvant therapy in patients with breast cancer. Furthermore, the meta-analysis showed that DL had higher predictive accuracy than ML + radiomics.
Mühlbauer2021	133	To assess the current evidence for the application of radiomics to renal masses, with a special focus on non- invasive classification of dignity and assessment of treatment response.	The application of radiomics seems promising for discrimination of renal tumor dignity. Shared data and open science may assist in improving reproducibility of future studies.

Pesapane2022	43	(1) To evaluate the methodological quality and the performance of prospective and retrospective studies published on MRI radiomics in predicting pathologic complete response (pCR) in breast cancer patients undergoing neoadjuvant therapy (NAT). (2) Moreover, assessing the quality of current radiomics studies on prediction of pCR to NAT may further promote the use of radiomics as a clinical tool in breast cancer patients.	MRI-radiomics may predict response to neoadjuvant therapy in breast cancer patients but the heterogeneity of the current studies is still substantial.
Ren2022	8	To evaluate and compare the diagnostic performance of apparent diffusion coefficient (ADC) values and MRI-based radiomics analysis for lymph node metastasis (LNM) detection in patients with cervical cancer (CC).	ADC values are more clinically promising because they are more easily accessible and widely applied, and exhibit a non-statistically significant trend to outperform radiomics analysis.
Sha2022	6	To analyze the diagnostic value of magnetic resonance imaging (MRI)-based radiomics for triple-negative breast cancer (TNBC) by conducting a meta-analysis.	MRI radiomics is an excellent diagnostic tool with high specificity for the diagnosis of TNBC.
Sohn2020	5	(1) To estimate the diagnostic accuracy of machine learning (ML)-based radiomics in differentiating high- grade gliomas (HGG) from low-grade gliomas (LGG). (2) To identify potential covariates that could affect the diagnostic accuracy of ML-based radiomic analysis in classifying gliomas.	This study demonstrates the excellent diagnostic performance of ML-based radiomics in differentiating HGG from LGG.
Ugga2021	23	(1) To systematically review and evaluate the methodological quality of studies using radiomics for diagnostic and predictive purposes in patients with intracranial meningioma. (2) To perform a meta-analysis of machine learning studies for the prediction of intracranial meningioma grading from pre-operative brain MRI.	Machine learning and radiomics have been proposed for multiple applications in the imaging of meningiomas, with promising results for preoperative lesion grading. However, future studies with adequate standardization and higher methodological quality are required prior to their introduction in clinical practice.
Ursprung2020	57	(1) To assess the methodological quality of radiomics studies investigating histological subtypes, therapy response, and survival in patients with renal cell carcinoma (RCC). (2) To determine the risk of bias in these radiomics studies.	Radiomics algorithms show promise for answering clinical questions where subjective interpretation is challenging or not established. However, the generalizability of findings to prospective cohorts needs to be demonstrated in future trials for progression towards clinical translation. Improved sharing of methods including code and

			images could facilitate independent validation of radiomics signatures.
Yang2022	12	(1) To evaluate the methodological quality and analyze the effectiveness of neoadjuvant chemotherapy in NPC among the published radiomics papers. (2) To predict the treatment response of neoadjuvant chemotherapy, using the radiomics method, in NPC.	Prediction response of neoadjuvant chemotherapy in NPC using machine learning and radiomics is beneficial in improving standardization and methodological quality before applying it to clinical practice.
Zhang2022A	14	To assess the diagnostic accuracy of artificial intelligence (AI) algorithms for non-invasive, preoperative prediction of MVI based on imaging data.	This meta-analysis demonstrates the high diagnostic accuracy of non-deep learning and deep learning methods for MVI status prediction and their promising potential for clinical decision-making. Deep learning models perform better than non-deep learning models in terms of the accuracy of MVI prediction, methodology, and cost-effectiveness.
Zhang2022B	13	To perform a meta-analysis to evaluate the diagnostic performance of machine learning(ML)-based radiomics of dynamic contrast-enhanced (DCE) magnetic resonance imaging (MRI) DCE-MRI in predicting axillary lymph node metastasis (ALNM) and sentinel lymph node metastasis(SLNM) in breast cancer.	ML-based radiomics of DCE-MRI has the potential to predict ALNM and SLNM accurately. The heterogeneity of the ALNM and SLNM diagnoses included between the studies is a major limitation.
Zhang2022C	28	(1) To assess the methodological quality and risk of bias of radiomics studies investigating the diagnostic performance in adrenal masses. (2) To determine the potential diagnostic value of radiomics in adrenal tumors by quantitative analysis.	The methodological quality and risk of bias of studies investigating the diagnostic performance of radiomics in adrenal tumors should be further improved in the future. CT-based radiomics has the potential benefits in differentiating malignant from benign adrenal tumors. The heterogeneity between the included studies was a major limitation to obtaining more accurate conclusions.
Zhong2021	12	To assess the methodological quality and risk of bias in radiomics studies investigating diagnosis, therapy response, and survival of patients with osteosarcoma.	The overall scientific quality of included studies is insufficient; however, radiomics remains a promising technology for predicting treatment response, which might guide therapeutic decision-making and related to prognosis. Improvements in study design, validation, and open science needs to be made to demonstrate the generalizability of findings and to achieve clinical applications.

			Widespread application of RQS, pre-trained RQS scoring procedure, and modification of RQS in response to clinical needs are necessary.
Zhong2022A	12	To evaluate the study quality and clinical value of radiomics studies on chondrosarcoma.	The current scientific and reporting quality of radiomics studies on chondrosarcoma was insufficient. Radiomics has potential in facilitating the optimization of operation decision-making in chondrosarcoma.
Zhong2022B	30	To assess the quality of pancreatitis radiomics research and test the feasibility of the evidence level rating tool.	More research on prognosis of acute pancreatitis is encouraged. The current pancreatitis radiomics studies have insufficient quality and share common scientific disadvantages. The evidence level rating is feasible and necessary for bringing the field of radiomics from preclinical research area to clinical stage.
Zhong2022C	29	To update the systematic review of radiomics in osteosarcoma.	The quality of osteosarcoma radiomics studies is insufficient. More investigation is needed before using radiomics to optimize osteosarcoma treatment. CLAIM is recommended to guide the design and reporting of radiomics research.
Zhong2022D	23	To assess the methodological quality and to evaluate the predictive performance of radiomics studies for preoperative prediction of microvascular invasion (MVI) in hepatocellular carcinoma (HCC).	Radiomics models show promising prediction performance for predicting MVI in HCC. However, improvements in standardization of methodology are required for feasibility confirmation and clinical translation.

Note: No. of primary studies refers to the radiomics studies included in the systematic reviews; those deep learning or machine learning-based not radiomics studies were not counted.

Supplementary Table S9 PRISMA adherence rate of included systematic reviews

PRISMA 2020	Bedrikovetski2021A	Bedrikovetski2021B	Bhandari2021	Cao2022	Castaldo2021	Chen2021	Cleere2022	Davey2021A	Davey2021B	Davey2021C	Deantonio2022	Gao2022A	Gao2022B	Han2022	Huang2020	Huang2021	Jia2022	Kao2021A	Kao2021B	Kao2022	Kothari2021	Kozikowski2021
**Title & Abstract (12)**	**10**	**7**	**8**	**8**	**8**	**7**	**6**	**6**	**6**	**6**	**5**	**7**	**7**	**7**	**8**	**7**	**9**	**5**	**7**	**6**	**8**	**9**
1/2.1	1	1	1	1	1	1	1	1	1	1	1	1	1	1	1	1	1	1	1	1	1	1
2.2	1	1	1	1	1	1	1	1	1	1	1	1	1	1	1	1	1	0	1	1	1	1
2.3	1	0	0	0	0	0	0	0	0	0	0	0	0	0	1	0	0	0	0	0	0	0
2.4	1	1	1	1	1	1	0	0	0	0	0	0	1	1	1	1	1	1	1	1	1	1
2.5	1	1	1	1	1	0	0	0	0	0	0	1	0	0	0	0	1	0	0	0	0	1
2.6	1	1	1	1	1	1	1	1	1	1	0	1	1	1	1	1	1	1	1	1	1	1
2.7	0	0	0	1	0	1	1	1	1	1	0	0	1	1	1	1	1	0	1	0	1	1
2.8	1	1	1	1	1	1	1	1	1	1	1	1	1	1	1	1	1	1	1	1	1	1
2.9	1	0	1	0	1	0	0	0	0	0	0	1	0	0	0	0	0	0	0	0	1	1
2.10	1	1	1	1	1	1	1	1	1	1	1	1	1	1	1	1	1	1	1	1	1	1
2.11	0	0	0	0	0	0	0	0	0	0	0	0	0	0	0	0	0	0	0	0	0	0
2.12	1	0	0	0	0	0	0	0	0	0	1	0	0	0	0	0	1	0	0	0	0	0
**Introduction (2)**	**2**	**2**	**2**	**2**	**2**	**2**	**2**	**2**	**2**	**2**	**2**	**2**	**2**	**2**	**2**	**2**	**2**	**2**	**2**	**2**	**2**	**2**
3	1	1	1	1	1	1	1	1	1	1	1	1	1	1	1	1	1	1	1	1	1	1
4	1	1	1	1	1	1	1	1	1	1	1	1	1	1	1	1	1	1	1	1	1	1
**Methods (17)**	**12**	**12**	**10**	**10**	**13**	**11**	**8**	**8**	**9**	**11**	**12**	**9**	**13**	**14**	**8**	**11**	**10**	**8**	**9**	**10**	**11**	**15**
5	1	1	1	1	1	1	1	1	1	1	1	1	1	1	1	1	1	1	1	1	1	1
6	1	1	1	1	1	1	1	1	1	1	1	1	1	1	1	1	1	1	1	1	1	1
7	1	1	1	0	0	0	0	0	0	0	1	1	0	1	0	0	1	0	0	0	1	1
8	1	1	1	0	1	1	1	1	1	1	1	1	1	1	1	0	0	0	0	1	1	1
9	0	0	0	1	1	1	0	0	0	1	1	0	1	1	0	1	0	0	0	0	1	1
10a	1	1	1	0	1	1	1	1	1	1	1	0	1	1	1	1	1	1	1	1	1	1
10b	1	1	1	0	1	1	1	1	1	1	1	0	1	1	1	1	1	1	1	1	1	1
11	1	1	1	1	1	1	0	0	0	1	1	1	1	1	0	0	1	0	1	1	0	1
12	1	1	1	1	1	1	1	1	1	1	1	1	1	1	1	1	1	1	1	1	1	1
13a	1	1	1	1	1	1	1	1	1	1	1	1	1	1	0	1	0	0	0	0	1	1
13b	1	1	0	0	1	1	0	0	1	1	1	1	1	1	1	1	0	1	1	1	1	1
13c	1	1	0	0	1	1	0	1	0	0	0	0	1	1	1	1	1	1	1	1	0	1

PRISMA 2020	Lee2022	Li2022A	Li2022B	Li2022C	Li2022D	Liang2022	Muhlbauer2021	Pesapane2022	Ren2022	Sha2022	Sohn2020	Ugga2021	Ursprung2020	Yang2022	Zhang2022A	Zhang2022B	Zhang2022C	Zhong2021	Zhong2022A	Zhong2022B	Zhong2022C	Zhong2022D
**Title & Abstract (12)**	**8**	**10**	**8**	**7**	**7**	**9**	**5**	**4**	**8**	**6**	**7**	**5**	**8**	**6**	**10**	**8**	**9**	**7**	**7**	**5**	**7**	**7**
1/2.1	1	1	1	1	1	1	1	1	1	1	1	1	1	1	1	1	1	1	1	1	1	1
2.2	1	1	1	1	1	1	1	0	1	1	1	1	1	1	1	1	1	1	1	1	1	1
2.3	0	0	0	0	0	0	0	0	0	0	0	0	0	0	0	0	0	0	0	0	0	0
2.4	1	1	1	0	1	1	0	0	1	0	1	0	0	0	1	0	1	1	1	0	1	0
2.5	0	1	1	1	0	1	0	0	0	0	0	0	1	1	1	1	1	1	1	1	1	1
2.6	1	1	1	1	1	1	0	1	1	1	1	1	1	1	1	1	1	1	1	0	1	1
2.7	1	1	1	1	1	1	0	0	1	1	0	0	1	0	1	1	0	0	0	0	0	1
2.8	1	1	1	1	1	1	1	1	1	1	1	1	1	1	1	1	1	1	1	1	1	1
2.9	1	1	0	0	0	0	1	0	1	0	1	0	1	0	1	1	1	0	0	0	0	0
2.10	1	1	1	1	1	1	1	1	1	1	1	1	1	1	1	1	1	1	1	1	1	1
2.11	0	0	0	0	0	0	0	0	0	0	0	0	0	0	0	0	0	0	0	0	0	0
2.12	0	1	0	0	0	1	0	0	0	0	0	0	0	0	1	0	1	0	0	0	0	0
**Introduction (2)**	**2**	**2**	**2**	**2**	**2**	**2**	**2**	**2**	**2**	**2**	**2**	**2**	**2**	**2**	**2**	**2**	**2**	**2**	**2**	**2**	**2**	**2**
3	1	1	1	1	1	1	1	1	1	1	1	1	1	1	1	1	1	1	1	1	1	1
4	1	1	1	1	1	1	1	1	1	1	1	1	1	1	1	1	1	1	1	1	1	1
**Methods (17)**	**12**	**13**	**12**	**11**	**8**	**13**	**11**	**14**	**14**	**11**	**12**	**11**	**13**	**12**	**12**	**11**	**15**	**14**	**15**	**15**	**15**	**10**
5	1	1	1	1	1	1	1	1	1	1	1	1	1	1	1	1	1	1	1	1	1	1
6	1	1	1	1	1	1	1	1	1	1	1	1	1	1	1	1	1	1	1	1	1	1
7	0	1	0	0	0	0	1	1	0	0	1	0	0	0	1	0	1	1	1	1	1	0
8	1	1	1	1	1	1	1	1	1	1	0	1	1	1	0	1	1	1	1	1	1	1
9	1	1	1	1	1	1	1	1	1	1	0	0	1	1	0	0	1	1	1	1	1	0
10a	1	1	1	1	1	1	1	1	1	1	1	1	1	1	1	1	1	1	1	1	1	1
10b	1	1	1	1	1	1	1	1	1	1	1	1	1	1	1	1	1	1	1	1	1	1
11	1	1	1	1	0	1	0	1	1	1	1	1	1	0	1	1	1	1	1	1	1	0
12	1	1	0	1	1	1	1	1	1	1	1	1	1	1	1	1	1	1	1	1	1	1
13a	1	0	0	1	0	0	1	1	1	0	1	1	1	1	0	0	1	1	1	1	1	1
13b	1	1	0	0	0	1	1	1	1	1	1	0	1	1	1	0	1	1	1	1	1	1
13c	0	1	1	0	1	0	1	0	1	0	1	1	1	1	1	0	1	1	1	1	1	0

13d	0	0	0	1	0	0	0	0	0	0	0	0	1	0	0	0	0	0	0	0	1	1	0	0	1	0	0	1	0	0	1	0	0	1	1	0	0	1	0	1	1	1	1	0
13e	0	0	0	1	1	0	0	0	0	0	0	0	1	1	0	1	1	0	0	0	0	1	1	1	1	1	0	1	0	1	1	1	1	0	0	1	1	1	1	0	0	0	0	1
13f	0	0	0	1	0	0	0	0	0	0	0	0	0	0	0	0	0	0	0	0	0	0	0	0	1	0	0	1	0	1	0	0	0	0	0	0	1	1	1	0	0	0	0	0
14	1	1	1	1	1	0	1	0	1	1	1	1	0	1	0	1	1	1	1	1	0	1	1	1	1	1	0	1	0	1	1	1	1	1	1	1	1	1	1	1	1	1	1	1
15	0	0	0	0	0	0	0	0	0	0	0	0	0	0	0	0	0	0	0	0	0	0	0	0	0	0	0	0	0	0	0	0	0	0	0	0	0	0	0	0	1	1	1	0
**Results (11)**	**6**	**6**	**4**	**7**	**7**	**5**	**5**	**5**	**4**	**4**	**6**	**7**	**5**	**6**	**6**	**8**	**7**	**6**	**6**	**6**	**4**	**7**	**6**	**7**	**8**	**8**	**7**	**8**	**4**	**8**	**7**	**7**	**7**	**6**	**6**	**7**	**8**	**8**	**8**	**6**	**7**	**7**	**7**	**7**
16a	1	1	1	1	1	1	1	1	1	1	1	1	1	1	1	1	1	1	1	1	1	1	1	1	1	1	1	1	1	1	1	1	1	1	1	1	1	1	1	1	1	1	1	1
16b	0	0	0	0	0	0	0	0	0	0	0	0	0	0	0	0	0	0	0	0	0	0	0	0	0	1	0	0	0	0	0	0	0	0	0	0	0	0	0	0	0	0	0	0
17	1	1	1	1	1	1	1	1	1	1	1	1	1	1	1	1	1	1	1	1	1	1	1	1	1	1	1	1	1	1	1	1	1	1	1	1	1	1	1	1	1	1	1	1
18	1	1	1	0	1	1	0	1	0	0	1	1	0	0	1	1	1	1	1	1	0	1	0	1	1	1	1	1	0	1	1	1	1	1	1	1	1	1	1	1	1	1	1	1
19	1	1	1	1	1	1	1	1	1	1	1	1	1	1	1	1	1	1	1	1	1	1	1	1	1	1	1	1	1	1	1	1	1	1	1	1	1	1	1	1	1	1	1	1
20a	0	0	0	0	0	0	0	0	0	0	0	0	0	0	0	0	0	0	0	0	0	0	0	0	0	0	0	0	0	0	0	0	0	0	0	0	0	0	0	0	0	0	0	0
20b	1	1	0	1	1	1	1	1	1	1	1	1	1	1	1	1	1	1	1	1	1	1	1	1	1	1	1	1	1	1	1	1	1	1	1	1	1	1	1	1	1	1	1	1
20c	0	0	0	1	1	0	0	0	0	0	0	1	1	1	0	1	1	0	0	0	0	1	1	1	1	1	1	1	0	1	1	1	1	0	0	1	1	1	1	0	0	0	0	1
20d	0	0	0	1	0	0	0	0	0	0	0	0	0	0	1	1	0	0	0	0	0	0	0	0	1	0	1	1	0	1	0	0	0	0	0	0	1	1	1	0	0	0	0	0
21	1	1	0	1	1	0	1	0	0	0	1	1	0	1	0	1	1	1	1	1	0	1	1	1	1	1	0	1	0	1	1	1	1	1	1	1	1	1	1	1	1	1	1	1
22	0	0	0	0	0	0	0	0	0	0	0	0	0	0	0	0	0	0	0	0	0	0	0	0	0	0	0	0	0	0	0	0	0	0	0	0	0	0	0	0	1	1	1	0
**Discussion (4)**	**3**	**4**	**3**	**4**	**4**	**4**	**4**	**3**	**3**	**3**	**3**	**4**	**3**	**4**	**3**	**3**	**3**	**3**	**3**	**4**	**4**	**3**	**3**	**3**	**3**	**3**	**4**	**4**	**3**	**4**	**4**	**3**	**4**	**4**	**4**	**4**	**3**	**4**	**4**	**4**	**4**	**4**	**4**	**4**
23a	1	1	1	1	1	1	1	1	1	1	1	1	1	1	1	1	1	1	1	1	1	1	1	1	1	1	1	1	1	1	1	1	1	1	1	1	1	1	1	1	1	1	1	1
23b	1	1	1	1	1	1	1	1	1	1	1	1	1	1	1	1	1	1	1	1	1	1	1	1	1	1	1	1	1	1	1	1	1	1	1	1	1	1	1	1	1	1	1	1
23c	0	1	0	1	1	1	1	0	0	0	0	1	0	1	0	0	0	0	0	1	1	0	0	0	0	0	1	1	0	1	1	0	1	1	1	1	0	1	1	1	1	1	1	1
23d	1	1	1	1	1	1	1	1	1	1	1	1	1	1	1	1	1	1	1	1	1	1	1	1	1	1	1	1	1	1	1	1	1	1	1	1	1	1	1	1	1	1	1	1
**Other Information (6)**	**6**	**1**	**0**	**0**	**1**	**6**	**2**	**5**	**1**	**1**	**5**	**4**	**1**	**2**	**2**	**1**	**5**	**1**	**2**	**2**	**4**	**5**	**4**	**5**	**4**	**4**	**2**	**4**	**6**	**6**	**1**	**1**	**2**	**1**	**6**	**2**	**5**	**2**	**5**	**4**	**4**	**5**	**6**	**1**
24a	1	0	0	0	0	1	0	1	0	0	1	1	0	0	0	0	1	0	0	0	1	1	1	1	1	1	0	1	1	1	0	0	0	0	1	0	1	0	1	1	1	1	1	0
24b	1	0	0	0	0	1	0	1	0	0	1	1	0	0	0	0	1	0	0	0	1	1	1	1	1	1	0	1	1	1	0	0	0	0	1	0	1	0	1	1	1	1	1	0
24c	1	0	0	0	0	1	0	1	0	0	1	1	0	0	0	0	1	0	0	0	1	1	1	1	1	1	0	1	1	1	0	0	0	0	1	0	1	0	1	1	1	1	1	0
25	1	0	0	0	0	1	0	0	0	0	0	0	0	1	0	0	0	0	1	1	0	1	0	0	0	0	0	0	1	1	0	0	0	0	1	0	0	0	0	0	0	0	1	0
26	1	1	0	0	1	1	1	1	1	1	1	1	1	1	1	1	1	1	1	1	1	1	1	1	1	1	1	1	1	1	1	1	1	1	1	1	1	1	1	1	1	1	1	1
27	1	0	0	0	0	1	1	1	0	0	1	0	0	0	1	0	1	0	0	0	0	0	0	1	0	0	1	0	1	1	0	0	1	0	1	1	1	1	1	0	0	1	1	0
Overall	39	32	27	31	35	35	27	29	25	27	33	33	31	35	29	32	36	25	29	30	33	41	35	40	37	35	30	40	31	38	36	30	34	29	39	33	40	35	43	37	39	38	41	31

Note: Overall = PRISMA 2022 for abstract + PRISMA 2022 checklist.

Supplementary Table S10 AMSTAR-2 ratings of included systematic reviews

AMSTAR-2 question	Bedrikovetski2021A	Bedrikovetski2021B	Bhandari2021	Cao2022	Castaldo2021	Chen2021	Cleere2022	Davey2021A	Davey2021B	Davey2021C	Deantonio2022	Gao2022A	Gao2022B	Han2022	Huang2020	Huang2021	Jia2022	Kao2021A	Kao2021B	Kao2022	Kothari2021	Kozikowski2021
1	Y	Y	Y	Y	Y	Y	Y	Y	Y	Y	Y	Y	Y	Y	Y	Y	Y	Y	Y	Y	Y	Y
2	PY	N	N	PY	PY	PY	N	PY	N	N	PY	PY	PY	PY	N	PY	Y	N	N	N	PY	Y
3	Y	Y	N	Y	N	Y	Y	Y	Y	Y	N	Y	N	Y	N	N	N	N	Y	Y	N	Y
4	PY	PY	PY	N	N	N	N	N	N	N	PY	PY	N	PY	N	N	PY	N	N	N	PY	PY
5	Y	Y	Y	N	Y	Y	Y	Y	Y	Y	Y	Y	Y	Y	Y	N	N	N	N	Y	Y	Y
6	N	N	N	Y	Y	Y	N	N	N	Y	Y	N	Y	Y	N	Y	N	N	N	N	Y	Y
7	N	N	N	N	N	N	N	N	N	N	N	N	N	N	N	N	N	N	N	N	N	N
8	PY	PY	PY	PY	PY	PY	PY	PY	PY	PY	PY	PY	PY	PY	PY	PY	PY	PY	PY	PY	PY	PY
9	Y	Y	Y	Y	Y	N	N	N	N	N	N	Y	Y	Y	Y	Y	Y	N	N	Y	N	Y
10	N	N	N	N	N	N	N	N	N	N	N	N	N	N	N	N	N	N	N	N	N	N
11	N	N	N	Y	Y	N	N	N	N	N	N	N	Y	Y	N	Y	Y	N	N	N	N	Y
12	N	N	N	N	N	N	N	N	N	N	N	N	N	N	N	N	N	N	N	N	N	N
13	Y	Y	Y	N	Y	N	Y	N	N	N	N	N	Y	N	Y	N	N	N	N	N	N	Y
14	N	N	N	Y	Y	N	N	N	N	N	N	Y	Y	Y	N	Y	Y	N	N	N	N	Y
15	Y	Y	N	Y	Y	N	Y	N	N	N	Y	Y	N	Y	N	Y	Y	Y	Y	Y	N	Y
16	Y	Y	N	N	Y	Y	Y	Y	Y	Y	Y	Y	Y	Y	Y	Y	Y	Y	Y	Y	Y	Y
Overall	CL	CL	CL	CL	CL	CL	CL	CL	CL	CL	CL	CL	CL	CL	CL	CL	CL	CL	CL	CL	CL	L

AMSTAR-2 question	Lee2022	Li2022A	Li2022B	Li2022C	Li2022D	Liang2022	Muhlbauer2021	Pesapane2022	Ren2022	Sha2022	Sohn2020	Ugga2021	Ursprung2020	Yang2022	Zhang2022A	Zhang2022B	Zhang2022C	Zhong2021	Zhong2022A	Zhong2022B	Zhong2022C	Zhong2022D
1	Y	Y	Y	Y	Y	Y	Y	Y	Y	Y	Y	Y	Y	Y	Y	Y	Y	Y	Y	Y	Y	Y
2	Y	Y	Y	Y	N	Y	PY	N	PY	PY	PY	N	PY	PY	Y	PY	Y	PY	PY	PY	PY	PY
3	Y	Y	Y	Y	N	Y	Y	Y	Y	Y	Y	Y	Y	Y	Y	Y	Y	Y	Y	Y	Y	Y
4	N	PY	N	N	N	N	PY	PY	N	N	PY	N	N	N	PY	N	PY	PY	PY	PY	PY	N
5	Y	Y	Y	Y	Y	Y	Y	Y	Y	Y	N	Y	Y	Y	N	Y	Y	Y	Y	Y	Y	Y
6	Y	Y	Y	Y	Y	Y	Y	Y	Y	Y	N	N	Y	Y	N	N	Y	Y	Y	Y	Y	N
7	N	N	N	Y	N	N	N	N	N	N	N	N	N	N	N	N	N	N	N	N	N	N
8	PY	PY	PY	PY	PY	PY	PY	PY	PY	PY	PY	PY	PY	PY	PY	PY	PY	PY	PY	PY	PY	PY
9	N	Y	Y	Y	Y	Y	N	N	Y	Y	Y	Y	Y	Y	Y	Y	Y	Y	Y	Y	Y	Y
10	N	N	N	N	N	N	N	N	N	N	N	N	Y	N	N	N	N	N	N	N	N	N
11	Y	Y	Y	Y	N	Y	N	Y	Y	Y	Y	N	N	Y	Y	Y	Y	N	N	N	N	Y
12	N	N	N	N	N	N	N	N	N	N	N	N	N	N	Y	N	N	N	N	N	N	N
13	N	Y	Y	Y	N	Y	Y	N	Y	Y	N	Y	Y	Y	Y	Y	Y	N	N	N	N	N
14	Y	Y	Y	Y	Y	Y	N	Y	Y	Y	Y	N	N	Y	Y	Y	Y	N	N	N	N	Y
15	Y	Y	Y	Y	N	Y	N	Y	Y	Y	Y	Y	Y	Y	Y	Y	Y	Y	Y	Y	Y	Y
16	Y	Y	Y	Y	Y	Y	Y	Y	Y	Y	Y	Y	Y	Y	Y	Y	Y	Y	Y	Y	Y	Y
Overall	CL	L	CL	L	CL	CL	CL	CL	CL	CL	CL	CL	CL	CL	L	CL	L	CL	CL	CL	CL	CL

Note: Y = Yes, PY = partial yes, N = no. H = high confidence, M = moderate confidence, L = low confidence, CL = critically low confidence.

Supplementary Table S11 ROBIS tool assessments of included systematic reviews

ROBIS tool	Bedrikovetski2021A	Bedrikovetski2021B	Bhandari2021	Cao2022	Castaldo2021	Chen2021	Cleere2022	Davey2021A	Davey2021B	Davey2021C	Deantonio2022	Gao2022A	Gao2022B	Han2022	Huang2020	Huang2021	Jia2022	Kao2021A	Kao2021B	Kao2022	Kothari2021	Kozikowski2021
SQ #1.1	Y	P Y	P Y	P Y	P Y	Y	P Y	Y	P Y	P Y	Y	Y	P Y	P Y	P Y	P Y	Y	P Y	P Y	P Y	Y	Y
SQ #1.2	P Y	P Y	P Y	P Y	P Y	P Y	P Y	P Y	P Y	P Y	P Y	P Y	P Y	P Y	P Y	P Y	P Y	P Y	P Y	P Y	P Y	P Y
SQ #1.3	P Y	P Y	P Y	P N	P N	P N	P N	P N	P N	P N	P Y	P Y	P N	P Y	P N	P N	P Y	P N	P N	P N	P Y	P Y
SQ #1.4	Y	Y	P Y	Y	P Y	Y	Y	Y	Y	Y	P Y	Y	P Y	Y	P Y	P Y	P Y	P Y	Y	Y	P Y	Y
SQ #1.5	Y	Y	Y	P Y	P Y	P Y	P Y	P Y	P Y	P Y	Y	Y	P Y	P Y	P Y	P Y	Y	P Y	P Y	P Y	Y	Y
Domain 1	L	L	L	U	U	U	U	U	U	U	L	L	U	L	U	U	L	U	U	U	L	L
SQ #2.1	Y	Y	Y	P Y	P Y	P Y	P Y	P Y	P Y	P Y	Y	Y	P Y	Y	P Y	P Y	Y	P Y	P Y	P Y	Y	Y
SQ #2.2	P Y	P Y	P Y	P N	P N	P N	P N	P N	P N	P N	P Y	P Y	P N	P Y	P N	P N	P Y	P N	P N	P N	P Y	P Y
SQ #2.3	Y	Y	Y	P N	P N	P N	P N	P N	P N	P N	Y	Y	P N	Y	P N	P N	Y	P N	P N	P N	Y	Y
SQ #2.4	P Y	P Y	P Y	P Y	P Y	P Y	P Y	P Y	P Y	P Y	P Y	P Y	P Y	P Y	P Y	P Y	P Y	P Y	P Y	P Y	P Y	P Y
SQ #2.5	Y	Y	Y	P N	Y	Y	Y	Y	Y	Y	Y	Y	Y	Y	Y	P N	P N	P N	P N	Y	Y	Y
Domain 2	L	L	L	H	U	U	U	U	U	U	L	L	U	L	U	H	U	H	H	U	L	L
SQ #3.1	P N	P N	P N	Y	Y	Y	P N	P N	P N	Y	Y	P N	Y	Y	P N	Y	P N	P N	P N	P N	Y	Y
SQ #3.2	Y	P Y	P Y	P Y	P Y	Y	Y	Y	P Y	P Y	Y	P Y	P Y	P Y	Y	P Y	Y	P Y	P Y	P Y	P Y	P Y
SQ #3.3	Y	Y	P Y	P Y	Y	Y	P Y	P Y	Y	Y	Y	Y	Y	Y	P Y	Y	P N	P Y	P Y	P Y	Y	Y
SQ #3.4	Y	Y	Y	Y	Y	N	N	N	N	N	N	Y	Y	Y	Y	Y	Y	N	N	Y	N	Y
SQ #3.5	Y	Y	Y	Y	Y	Y	P N	P N	P N	Y	Y	Y	Y	Y	P N	P N	Y	P N	Y	Y	P N	Y
Domain 3	U	U	U	L	L	H	H	H	H	H	H	U	L	L	U	U	U	H	H	U	H	L

ROBIS tool	Lee2022	Li2022A	Li2022B	Li2022C	Li2022D	Liang2022	Muhlbauer2021	Pesapane2022	Ren2022	Sha2022	Sohn2020	Ugga2021	Ursprung2020	Yang2022	Zhang2022A	Zhang2022B	Zhang2022C	Zhong2021	Zhong2022A	Zhong2022B	Zhong2022C	Zhong2022D
SQ #1.1	Y	Y	Y	Y	P Y	Y	Y	Y	P Y	P Y	P Y	P Y	Y	P Y	Y	P Y	Y	Y	Y	Y	Y	P Y
SQ #1.2	P Y	P Y	P Y	P Y	P Y	P Y	P Y	P Y	P Y	P Y	P Y	P Y	P Y	P Y	P Y	P Y	P Y	P Y	P Y	P Y	P Y	P Y
SQ #1.3	P N	P Y	P N	P N	P N	P N	P Y	P Y	P N	P N	P Y	P N	P N	P N	P Y	P N	P Y	P Y	P Y	P Y	P Y	P N
SQ #1.4	Y	Y	Y	Y	P Y	Y	Y	Y	Y	Y	Y	Y	Y	Y	Y	Y	Y	Y	Y	Y	Y	Y
SQ #1.5	P Y	Y	P Y	P Y	P Y	P Y	Y	Y	P Y	P Y	Y	P Y	P Y	P Y	Y	P Y	Y	Y	Y	Y	Y	P Y
Domain 1	U	L	U	U	U	U	L	L	U	U	L	U	U	U	L	U	L	L	L	L	L	U
SQ #2.1	P Y	Y	P Y	P Y	P Y	P Y	Y	Y	P Y	P Y	Y	P Y	P Y	P Y	Y	P Y	Y	Y	Y	Y	Y	P Y
SQ #2.2	P N	P Y	P N	P N	P N	P N	P Y	P N	P N	P N	P Y	P N	P N	P N	P Y	P N	P N	P Y	P Y	P Y	P Y	P N
SQ #2.3	P N	Y	P N	P N	P N	P N	Y	Y	P N	P N	Y	P N	P N	P N	Y	P N	Y	Y	Y	Y	Y	P N
SQ #2.4	P Y	P Y	P Y	P Y	P Y	P Y	P Y	P Y	P Y	P Y	P Y	P Y	P Y	P Y	P Y	P Y	P Y	P Y	P Y	P Y	P Y	P Y
SQ #2.5	Y	Y	Y	Y	Y	Y	Y	Y	Y	Y	P N	Y	Y	Y	P N	Y	Y	Y	Y	Y	Y	Y
Domain 2	U	L	U	U	U	U	L	U	U	U	U	U	U	U	U	U	U	L	L	L	L	U
SQ #3.1	Y	Y	Y	Y	Y	Y	Y	Y	Y	Y	P N	P N	Y	Y	P N	P N	Y	Y	Y	Y	Y	P N
SQ #3.2	P Y	Y	P Y	P Y	Y	P Y	Y	Y	P Y	P Y	Y	P Y	Y	Y	Y	Y	Y	P Y	P Y	Y	Y	P Y
SQ #3.3	Y	P Y	P N	P Y	P N	P Y	Y	Y	Y	P Y	Y	P Y	Y	Y	P Y	P N	Y	Y	Y	Y	Y	Y
SQ #3.4	N	Y	Y	Y	Y	Y	N	N	Y	Y	Y	Y	Y	Y	Y	Y	Y	Y	Y	Y	Y	Y
SQ #3.5	Y	Y	Y	Y	P N	Y	P N	Y	Y	Y	Y	Y	Y	P N	Y	Y	Y	Y	Y	Y	Y	P N
Domain 3	H	L	U	L	U	L	H	H	L	L	U	U	L	U	U	U	L	L	L	L	L	U

SQ #4.1	Y	Y	PY	PY	Y	Y	PY	PY	Y	Y	Y	Y	Y	Y	Y	Y	PY	Y	Y	Y	Y	Y	Y	Y	PY	PY	PY	Y	Y	Y	Y	Y	Y	PY	Y	Y	Y	PY	Y	Y	Y	Y	Y	Y
SQ #4.2	PY	PY	N	PY	PY	PY	PY	PY	N	N	PY	N	PY	PY	N	N	PY	PY	PY	PY	PY	PY	PY	PY	PY	PY	N	PY	PY	PY	PY	PY	PY	PY	PY	PY	PY	PY	PY	PY	PY	PY	PY	PY
SQ #4.3	PY	PY	PY	Y	PY	PY	PY	PY	PY	PY	PY	PY	Y	PY	PY	PY	PY	PY	PY	PY	Y	Y	PY	PY	Y	PY	PY	Y	PY	PY	Y	PY	PY	Y	Y	PY	PY	Y	PY	Y	Y	Y	Y	PY
SQ #4.4	N	N	N	Y	Y	N	N	N	N	N	N	N	Y	Y	N	Y	Y	N	N	N	N	Y	Y	Y	Y	Y	N	Y	N	Y	Y	Y	Y	N	N	Y	Y	Y	Y	N	N	N	N	Y
SQ #4.5	Y	Y	Y	Y	Y	N	Y	N	Y	Y	Y	Y	N	Y	N	Y	Y	Y	Y	Y	N	Y	Y	Y	Y	Y	N	Y	N	Y	Y	Y	Y	Y	Y	Y	Y	Y	Y	Y	Y	Y	Y	Y
SQ #4.6	PN	PN	PN	PY	PN	N	N	N	N	N	N	PN	PN	PN	PN	PN	PN	N	N	PN	N	PN	N	PN	PY	PN	PN	PY	N	N	N	PN	PN	PN	PN	PN	PY	PY	PY	PN	PN	PN	PN	PN
Domain 4	H	H	H	L	U	H	H	H	H	H	H	H	H	U	H	H	U	H	H	H	H	U	H	U	L	U	H	L	H	H	U	U	U	H	H	U	L	L	L	H	H	H	H	U
SQ #A	N	N	N	N	PY	N	N	N	N	N	N	N	N	PY	N	N	PY	N	N	N	N	PY	N	PY	PY	PY	N	PY	N	N	PY	PY	PY	N	N	PY	PY	PY	PY	N	N	N	N	PY
SQ #B	Y	Y	Y	Y	Y	PY	PY	PY	PY	PY	PY	Y	Y	Y	Y	Y	Y	PY	PY	Y	PY	Y	PY	Y	Y	Y	Y	Y	PY	PY	Y	Y	Y	Y	Y	Y	Y	Y	Y	Y	Y	Y	Y	Y
SQ #C	PY	PY	PY	Y	PY	PY	PY	PY	PY	PY	PY	PY	PY	PY	PY	Y	PY	PY	PY	PY	PY	PY	PY	PY	Y	PY	PY	Y	PY	Y	PY	PY	PY	PY	PY	PY	Y	Y	Y	PY	PY	PY	PY	PY
Overall	H	H	H	H	U	H	H	H	H	H	H	H	H	U	H	H	U	H	H	H	H	U	H	U	L	U	H	L	H	H	U	U	U	H	H	U	L	L	L	H	H	H	H	U

Note: SQ = signaling question. Y = Yes, PY = probably yes, PN = probably no, N = no. H = high risk of bias, U = unclear risk of bias, L = low risk of bias.

Gao2022A	23	To systematically evaluate the prognostic prediction accuracy of radiomics features extracted from pre-treatment imaging in patients with pancreatic ductal adenocarcinoma (PDAC).	First-order entropy was significantly associated with overall survival (OS) and might improve the accuracy of PDAC prognosis prediction. Existing studies were poorly validated, and it should be noted in future studies. Modification of PROBAST for radiomics studies is necessary since the strict requirements of prospective study design may not be applicable to the demand for a large sample size in the model construction stage.
Gao2022B	37	To systematically review the value of radiomics in the diagnosis of glioblastoma.	The current evidence shows that radiomics provides good diagnostic accuracy for glioblastoma. Due to the limited quality and quantity of the included studies, more high-quality studies are required to verify the above conclusions.
Han2022	14	To reveal a radiogenomic correlation between the presence of the T2-fluid-attenuated inversion recovery resection (T2-FLAIR) mismatch sign on MR images and isocitrate dehydrogenase (IDH) mutation status in adult patients with lower-grade gliomas (LGGs).	The T2-FLAIR mismatch sign was an insensitive but highly specific marker for IDHmut-Noncodel and IDH-Mutation LGGs, whereas it was not a useful marker for IDHmut-Codel LGGs. The findings might identify the T2-FLAIR mismatch sign as a non-invasive imaging biomarker for the selection of patients with IDH-mutant LGGs.
Huang2020	9	To compare the predictive power between radiomics and non-radiomics (conventional imaging and functional imaging methods) for preoperative evaluation of microvascular invasion (MVI) in hepatocellular carcinoma (HCC).	The imaging method is feasible to predict the MVI state of HCC. Radiomics method based on medical image data is a promising application in clinical practice and can provide quantifiable image features. With the help of these features, highly consistent prediction performance will be achieved in anticipation.
Huang2021	15	To assess the diagnostic performance of radiomics using machine learning algorithms to predict the methylation status of the O6-methylguanine-DNA methyltransferase (MGMT) promoter in glioma patients.	This meta-analysis demonstrated that machine learning is a promising, reliable and repeatable candidate method for predicting MGMT promoter methylation status in glioma and showed a higher performance than non-machine learning methods.
Jia2022	16	(1) To evaluate the diagnostic accuracy of artificial intelligence (AI) models with MRI in predicting pathological complete response(pCR) to neoadjuvant chemoradiotherapy (nCRT) in patients with rectal cancer. (2) To assessed the methodological quality of the models.	Radiomics is a promising noninvasive method with high value in predicting pathological response to nCRT in patients with rectal cancer. DL models have higher predictive accuracy than radiomics models, and combined models incorporating clinical factors have

			higher diagnostic accuracy than radiomics models alone. In the future, prospective, large-scale, multicenter investigations using radiomics approaches will strengthen the diagnostic power of pCR.
Kao2021A	10	To investigate the predictive power of radiomics in esophageal cancer.	Using radiomics to predict complete pathological response after neoadjuvant chemoradiotherapy in esophageal cancer is feasible. In the future, prospective, multicenter studies should be carried out for predicting pathological complete response in patients with esophageal cancer
Kao2021B	7	To use computerized tomography (CT)-based radiomics models to differentiate COVID-19 pneumonia from other viral pneumonia infections.	Our meta-analysis showed that CT-based radiomics feature models can successfully differentiate COVID-19 from other viral pneumonias.
Kao2022	8	To investigate the predictive power of a CT-based radiomics model in determining COVID-19 severity.	This meta-analysis demonstrated that CT-based radiomics models might be helpful for predicting the severity of COVID-19 pneumonia.
Kothari2021	40	To perform a systematic review and meta-analysis of the prognostic value of radiomics models in patients with non-small cell lung cancer (NSCLC) treated with curative intent radiotherapy.	Based on this review, radiomics based models for lung cancer have to date demonstrated modest prognostic capabilities. Future research should consider using standardized radiomics features, robust feature selection and model development, and deep learning techniques, absolving the need for pre-defined features, to improve imaging-based models.
Kozikowski2021	8	To systematically review the diagnostic performance of radiomic techniques in predicting muscle-invasive bladder cancer (MIBC).	Radiomics shows high diagnostic performance in predicting MIBC. Despite differences in approaches, radiomic models were relatively homogeneous in their diagnostic accuracy. With further improvements, radiomics has the potential to become a useful adjunct in clinical management of bladder cancer.
Lee2022	10	To comprehensively assess the prognostic value of MRI-based radiomics for untreated nasopharyngeal carcinoma (NPC).	MRI-based radiomics shows good prognostic performance in predicting the PFS of patients with untreated NPC. However, more consistent and robust study protocols are necessary to validate the prognostic role of radiomics for NPC.

Li2022A	22	To perform a meta-analysis to investigate the diagnostic performance of radiomics for the preoperative evaluation of MVI in HCC and the effect of potential factors.	Radiomics is a promising noninvasive method that has high preoperative diagnostic performance for MVI status. Radiomics based on CT and MRI had a comparable predictive performance for MVI in HCC. Prospective, large-scale and multicenter studies with radiomics methods will improve the diagnostic power for MVI in the future.
Li2022B	12	To evaluate the ability of preoperative MRI-based radiomic features in predicting lymph node metastasis (LNM) in patients with cervical cancer.	Our meta-analysis showed that preoperative MRI-based radiomic features performs well in predicting LNM in patients with cervical cancer. This noninvasive and convenient tool may be used to facilitate preoperative identification of LNM.
Li2022C	17	(1) To evaluate the diagnostic performance of radiomics in differentiating high-grade glioma from brain metastasis and how to improve the model. (2) To assess the methodological quality of radiomics studies and explore ways of embracing the clinical application of radiomics.	Radiomics can accurately differentiate high-grade glioma from brain metastasis. The adoption of standardized workflow to avoid potential data leakage as well as the integration of clinical features and radiomics are advised to consider in future studies.
Li2022D	19	To evaluate the preoperative predictive value of radiomics in the diagnosis of breast cancer (BC).	Radiomics has shown excellent diagnostic performance in the preoperative prediction of BC and is expected to be a promising method in clinical practice.
Liang2022	15	To determine the diagnostic accuracy of machine learning (ML) models with MRI in predicting pathological response to neoadjuvant chemotherapy in patients with breast cancer. Furthermore, we compared the pathologic complete response (pCR) prediction performance of ML + radiomics with that of a deep learning (DL) algorithm.	ML applied to MRI enabled moderate accuracy in predicting pathological response to neoadjuvant therapy in patients with breast cancer. Furthermore, the meta-analysis showed that DL had higher predictive accuracy than ML + radiomics.
Mühlbauer2021	133	To assess the current evidence for the application of radiomics to renal masses, with a special focus on non- invasive classification of dignity and assessment of treatment response.	The application of radiomics seems promising for discrimination of renal tumor dignity. Shared data and open science may assist in improving reproducibility of future studies.

Pesapane2022	43	(1) To evaluate the methodological quality and the performance of prospective and retrospective studies published on MRI radiomics in predicting pathologic complete response (pCR) in breast cancer patients undergoing neoadjuvant therapy (NAT). (2) Moreover, assessing the quality of current radiomics studies on prediction of pCR to NAT may further promote the use of radiomics as a clinical tool in breast cancer patients.	MRI-radiomics may predict response to neoadjuvant therapy in breast cancer patients but the heterogeneity of the current studies is still substantial.
Ren2022	8	To evaluate and compare the diagnostic performance of apparent diffusion coefficient (ADC) values and MRI-based radiomics analysis for lymph node metastasis (LNM) detection in patients with cervical cancer (CC).	ADC values are more clinically promising because they are more easily accessible and widely applied, and exhibit a non-statistically significant trend to outperform radiomics analysis.
Sha2022	6	To analyze the diagnostic value of magnetic resonance imaging (MRI)-based radiomics for triple-negative breast cancer (TNBC) by conducting a meta-analysis.	MRI radiomics is an excellent diagnostic tool with high specificity for the diagnosis of TNBC.
Sohn2020	5	(1) To estimate the diagnostic accuracy of machine learning (ML)-based radiomics in differentiating high- grade gliomas (HGG) from low-grade gliomas (LGG). (2) To identify potential covariates that could affect the diagnostic accuracy of ML-based radiomic analysis in classifying gliomas.	This study demonstrates the excellent diagnostic performance of ML-based radiomics in differentiating HGG from LGG.
Ugga2021	23	(1) To systematically review and evaluate the methodological quality of studies using radiomics for diagnostic and predictive purposes in patients with intracranial meningioma. (2) To perform a meta-analysis of machine learning studies for the prediction of intracranial meningioma grading from pre-operative brain MRI.	Machine learning and radiomics have been proposed for multiple applications in the imaging of meningiomas, with promising results for preoperative lesion grading. However, future studies with adequate standardization and higher methodological quality are required prior to their introduction in clinical practice.
Ursprung2020	57	(1) To assess the methodological quality of radiomics studies investigating histological subtypes, therapy response, and survival in patients with renal cell carcinoma (RCC). (2) To determine the risk of bias in these radiomics studies.	Radiomics algorithms show promise for answering clinical questions where subjective interpretation is challenging or not established. However, the generalizability of findings to prospective cohorts needs to be demonstrated in future trials for progression towards clinical translation. Improved sharing of methods including code and

			images could facilitate independent validation of radiomics signatures.
Yang2022	12	(1) To evaluate the methodological quality and analyze the effectiveness of neoadjuvant chemotherapy in NPC among the published radiomics papers. (2) To predict the treatment response of neoadjuvant chemotherapy, using the radiomics method, in NPC.	Prediction response of neoadjuvant chemotherapy in NPC using machine learning and radiomics is beneficial in improving standardization and methodological quality before applying it to clinical practice.
Zhang2022A	14	To assess the diagnostic accuracy of artificial intelligence (AI) algorithms for non-invasive, preoperative prediction of MVI based on imaging data.	This meta-analysis demonstrates the high diagnostic accuracy of non-deep learning and deep learning methods for MVI status prediction and their promising potential for clinical decision-making. Deep learning models perform better than non-deep learning models in terms of the accuracy of MVI prediction, methodology, and cost-effectiveness.
Zhang2022B	13	To perform a meta-analysis to evaluate the diagnostic performance of machine learning(ML)-based radiomics of dynamic contrast-enhanced (DCE) magnetic resonance imaging (MRI) DCE-MRI in predicting axillary lymph node metastasis (ALNM) and sentinel lymph node metastasis(SLNM) in breast cancer.	ML-based radiomics of DCE-MRI has the potential to predict ALNM and SLNM accurately. The heterogeneity of the ALNM and SLNM diagnoses included between the studies is a major limitation.
Zhang2022C	28	(1) To assess the methodological quality and risk of bias of radiomics studies investigating the diagnostic performance in adrenal masses. (2) To determine the potential diagnostic value of radiomics in adrenal tumors by quantitative analysis.	The methodological quality and risk of bias of studies investigating the diagnostic performance of radiomics in adrenal tumors should be further improved in the future. CT-based radiomics has the potential benefits in differentiating malignant from benign adrenal tumors. The heterogeneity between the included studies was a major limitation to obtaining more accurate conclusions.
Zhong2021	12	To assess the methodological quality and risk of bias in radiomics studies investigating diagnosis, therapy response, and survival of patients with osteosarcoma.	The overall scientific quality of included studies is insufficient; however, radiomics remains a promising technology for predicting treatment response, which might guide therapeutic decision-making and related to prognosis. Improvements in study design, validation, and open science needs to be made to demonstrate the generalizability of findings and to achieve clinical applications.

			Widespread application of RQS, pre-trained RQS scoring procedure, and modification of RQS in response to clinical needs are necessary.
Zhong2022A	12	To evaluate the study quality and clinical value of radiomics studies on chondrosarcoma.	The current scientific and reporting quality of radiomics studies on chondrosarcoma was insufficient. Radiomics has potential in facilitating the optimization of operation decision-making in chondrosarcoma.
Zhong2022B	30	To assess the quality of pancreatitis radiomics research and test the feasibility of the evidence level rating tool.	More research on prognosis of acute pancreatitis is encouraged. The current pancreatitis radiomics studies have insufficient quality and share common scientific disadvantages. The evidence level rating is feasible and necessary for bringing the field of radiomics from preclinical research area to clinical stage.
Zhong2022C	29	To update the systematic review of radiomics in osteosarcoma.	The quality of osteosarcoma radiomics studies is insufficient. More investigation is needed before using radiomics to optimize osteosarcoma treatment. CLAIM is recommended to guide the design and reporting of radiomics research.
Zhong2022D	23	To assess the methodological quality and to evaluate the predictive performance of radiomics studies for preoperative prediction of microvascular invasion (MVI) in hepatocellular carcinoma (HCC).	Radiomics models show promising prediction performance for predicting MVI in HCC. However, improvements in standardization of methodology are required for feasibility confirmation and clinical translation.

Note: No. of primary studies refers to the radiomics studies included in the systematic reviews; those deep learning or machine learning-based not radiomics studies were not counted.

Supplementary Table S9 PRISMA adherence rate of included systematic reviews

PRISMA 2020	Bedrikovetski2021A	Bedrikovetski2021B	Bhandari2021	Cao2022	Castaldo2021	Chen2021	Cleere2022	Davey2021A	Davey2021B	Davey2021C	Deantonio2022	Gao2022A	Gao2022B	Han2022	Huang2020	Huang2021	Jia2022	Kao2021A	Kao2021B	Kao2022	Kothari2021	Kozikowski2021	Lee2022
Title & Abstract (12)	10	7	8	8	8	7	6	6	6	6	5	7	7	7	8	7	9	5	7	6	8	9	8
1/2.1	1	1	1	1	1	1	1	1	1	1	1	1	1	1	1	1	1	1	1	1	1	1	1
2.2	1	1	1	1	1	1	1	1	1	1	1	1	1	1	1	1	1	0	1	1	1	1	1
2.3	1	0	0	0	0	0	0	0	0	0	0	0	0	0	1	0	0	0	0	0	0	0	0
2.4	1	1	1	1	1	1	0	0	0	0	0	0	1	1	1	1	1	1	1	1	1	1	1
2.5	1	1	1	1	1	0	0	0	0	0	0	1	0	0	0	0	1	0	0	0	0	1	0
2.6	1	1	1	1	1	1	1	1	1	1	0	1	1	1	1	1	1	1	1	1	1	1	1
2.7	0	0	0	1	0	1	1	1	1	1	0	0	1	1	1	1	1	0	1	0	1	1	1
2.8	1	1	1	1	1	1	1	1	1	1	1	1	1	1	1	1	1	1	1	1	1	1	1
2.9	1	0	1	0	1	0	0	0	0	0	0	1	0	0	0	0	0	0	0	0	1	1	1
2.10	1	1	1	1	1	1	1	1	1	1	1	1	1	1	1	1	1	1	1	1	1	1	1
2.11	0	0	0	0	0	0	0	0	0	0	0	0	0	0	0	0	0	0	0	0	0	0	0
2.12	1	0	0	0	0	0	0	0	0	0	1	0	0	0	0	0	1	0	0	0	0	0	0
Introduction (2)	2	2	2	2	2	2	2	2	2	2	2	2	2	2	2	2	2	2	2	2	2	2	2
3	1	1	1	1	1	1	1	1	1	1	1	1	1	1	1	1	1	1	1	1	1	1	1
4	1	1	1	1	1	1	1	1	1	1	1	1	1	1	1	1	1	1	1	1	1	1	1
Methods (17)	12	12	10	10	13	11	8	8	9	11	12	9	13	14	8	11	10	8	9	10	11	15	12
5	1	1	1	1	1	1	1	1	1	1	1	1	1	1	1	1	1	1	1	1	1	1	1
6	1	1	1	1	1	1	1	1	1	1	1	1	1	1	1	1	1	1	1	1	1	1	1
7	1	1	1	0	0	0	0	0	0	0	1	1	0	1	0	0	1	0	0	0	1	1	0
8	1	1	1	0	1	1	1	1	1	1	1	1	1	1	1	0	0	0	0	1	1	1	1
9	0	0	0	1	1	1	0	0	0	1	1	0	1	1	0	1	0	0	0	0	1	1	1
10a	1	1	1	0	1	1	1	1	1	1	1	0	1	1	1	1	1	1	1	1	1	1	1
10b	1	1	1	0	1	1	1	1	1	1	1	0	1	1	1	1	1	1	1	1	1	1	1
11	1	1	1	1	1	1	0	0	0	1	1	1	1	1	0	0	1	0	1	1	0	1	1
12	1	1	1	1	1	1	1	1	1	1	1	1	1	1	1	1	1	1	1	1	1	1	1
13a	1	1	1	1	1	1	1	1	1	1	1	1	1	1	0	1	0	0	0	0	1	1	1
13b	1	1	0	0	1	1	0	0	1	1	1	1	1	1	1	1	0	1	1	1	1	1	1
13c	1	1	0	0	1	1	0	1	0	0	0	0	1	1	1	1	1	1	1	1	0	1	0

PRISMA 2020	Li2022A	Li2022B	Li2022C	Li2022D	Liang2022	Muhlbauer2021	Pesapane2022	Ren2022	Sha2022	Sohn2020	Ugga2021	Ursprung2020	Yang2022	Zhang2022A	Zhang2022B	Zhang2022C	Zhong2021	Zhong2022A	Zhong2022B	Zhong2022C	Zhong2022D
Title & Abstract (12)	10	8	7	7	9	5	4	8	6	7	5	8	6	10	8	9	7	7	5	7	7
1/2.1	1	1	1	1	1	1	1	1	1	1	1	1	1	1	1	1	1	1	1	1	1
2.2	1	1	1	1	1	1	0	1	1	1	1	1	1	1	1	1	1	1	1	1	1
2.3	0	0	0	0	0	0	0	0	0	0	0	0	0	0	0	0	0	0	0	0	0
2.4	1	1	0	1	1	0	0	1	0	1	0	0	0	1	0	1	1	1	0	1	0
2.5	1	1	1	0	1	0	0	0	0	0	0	1	1	1	1	1	1	1	1	1	1
2.6	1	1	1	1	1	0	1	1	1	1	1	1	1	1	1	1	1	1	0	1	1
2.7	1	1	1	1	1	0	0	1	1	0	0	1	0	1	1	0	0	0	0	0	1
2.8	1	1	1	1	1	1	1	1	1	1	1	1	1	1	1	1	1	1	1	1	1
2.9	1	0	0	0	0	1	0	1	0	1	0	1	0	1	1	1	0	0	0	0	0
2.10	1	1	1	1	1	1	1	1	1	1	1	1	1	1	1	1	1	1	1	1	1
2.11	0	0	0	0	0	0	0	0	0	0	0	0	0	0	0	0	0	0	0	0	0
2.12	1	0	0	0	1	0	0	0	0	0	0	0	0	1	0	1	0	0	0	0	0
Introduction (2)	2	2	2	2	2	2	2	2	2	2	2	2	2	2	2	2	2	2	2	2	2
3	1	1	1	1	1	1	1	1	1	1	1	1	1	1	1	1	1	1	1	1	1
4	1	1	1	1	1	1	1	1	1	1	1	1	1	1	1	1	1	1	1	1	1
Methods (17)	13	12	11	8	13	11	14	14	11	12	11	13	12	12	11	15	14	15	15	15	10
5	1	1	1	1	1	1	1	1	1	1	1	1	1	1	1	1	1	1	1	1	1
6	1	1	1	1	1	1	1	1	1	1	1	1	1	1	1	1	1	1	1	1	1
7	1	0	0	0	0	1	1	0	0	1	0	0	0	1	0	1	1	1	1	1	0
8	1	1	1	1	1	1	1	1	1	0	1	1	1	0	1	1	1	1	1	1	1
9	1	1	1	1	1	1	1	1	1	0	0	1	1	0	0	1	1	1	1	1	0
10a	1	1	1	1	1	1	1	1	1	1	1	1	1	1	1	1	1	1	1	1	1
10b	1	1	1	1	1	1	1	1	1	1	1	1	1	1	1	1	1	1	1	1	1
11	1	1	1	0	1	0	1	1	1	1	1	1	0	1	1	1	1	1	1	1	0
12	1	0	1	1	1	1	1	1	1	1	1	1	1	1	1	1	1	1	1	1	1
13a	0	0	1	0	0	1	1	1	0	1	1	1	1	0	0	1	1	1	1	1	1
13b	1	0	0	0	1	1	1	1	1	1	0	1	1	1	0	1	1	1	1	1	1
13c	1	1	0	1	0	1	0	1	0	1	1	1	1	1	0	1	1	1	1	1	0

13d	0	0	0	1	0	0	0	0	0	0	0	0	1	0	0	0	0	0	0	0	1	1	0	0	1	0	0	1	0	0	1	0	0	1	1	0	0	1	0	1	1	1	1	0
13e	0	0	0	1	1	0	0	0	0	0	0	0	1	1	0	1	1	0	0	0	0	1	1	1	1	1	0	1	0	1	1	1	1	0	0	1	1	1	1	0	0	0	0	1
13f	0	0	0	1	0	0	0	0	0	0	0	0	0	0	0	0	0	0	0	0	0	0	0	0	1	0	0	1	0	1	0	0	0	0	0	0	1	1	1	0	0	0	0	0
14	1	1	1	1	1	0	1	0	1	1	1	1	0	1	0	1	1	1	1	1	0	1	1	1	1	1	0	1	0	1	1	1	1	1	1	1	1	1	1	1	1	1	1	1
15	0	0	0	0	0	0	0	0	0	0	0	0	0	0	0	0	0	0	0	0	0	0	0	0	0	0	0	0	0	0	0	0	0	0	0	0	0	0	0	0	1	1	1	0
**Result<br>s (11)**	**6**	**6**	**4**	**7**	**7**	**5**	**5**	**5**	**4**	**4**	**6**	**7**	**5**	**6**	**6**	**8**	**7**	**6**	**6**	**6**	**4**	**7**	**6**	**7**	**8**	**8**	**7**	**8**	**4**	**8**	**7**	**7**	**7**	**6**	**6**	**7**	**8**	**8**	**8**	**6**	**7**	**7**	**7**	**7**
16a	1	1	1	1	1	1	1	1	1	1	1	1	1	1	1	1	1	1	1	1	1	1	1	1	1	1	1	1	1	1	1	1	1	1	1	1	1	1	1	1	1	1	1	1
16b	0	0	0	0	0	0	0	0	0	0	0	0	0	0	0	0	0	0	0	0	0	0	0	0	0	1	0	0	0	0	0	0	0	0	0	0	0	0	0	0	0	0	0	0
17	1	1	1	1	1	1	1	1	1	1	1	1	1	1	1	1	1	1	1	1	1	1	1	1	1	1	1	1	1	1	1	1	1	1	1	1	1	1	1	1	1	1	1	1
18	1	1	1	0	1	1	0	1	0	0	1	1	0	0	1	1	1	1	1	1	0	1	0	1	1	1	1	1	0	1	1	1	1	1	1	1	1	1	1	1	1	1	1	1
19	1	1	1	1	1	1	1	1	1	1	1	1	1	1	1	1	1	1	1	1	1	1	1	1	1	1	1	1	1	1	1	1	1	1	1	1	1	1	1	1	1	1	1	1
20a	0	0	0	0	0	0	0	0	0	0	0	0	0	0	0	0	0	0	0	0	0	0	0	0	0	0	0	0	0	0	0	0	0	0	0	0	0	0	0	0	0	0	0	0
20b	1	1	0	1	1	1	1	1	1	1	1	1	1	1	1	1	1	1	1	1	1	1	1	1	1	1	1	1	1	1	1	1	1	1	1	1	1	1	1	1	1	1	1	1
20c	0	0	0	1	1	0	0	0	0	0	0	1	1	1	0	1	1	0	0	0	0	1	1	1	1	1	1	1	0	1	1	1	1	0	0	1	1	1	1	0	0	0	0	1
20d	0	0	0	1	0	0	0	0	0	0	0	0	0	0	1	1	0	0	0	0	0	0	0	0	1	0	1	1	0	1	0	0	0	0	0	0	1	1	1	0	0	0	0	0
21	1	1	0	1	1	0	1	0	0	0	1	1	0	1	0	1	1	1	1	1	0	1	1	1	1	1	0	1	0	1	1	1	1	1	1	1	1	1	1	1	1	1	1	1
22	0	0	0	0	0	0	0	0	0	0	0	0	0	0	0	0	0	0	0	0	0	0	0	0	0	0	0	0	0	0	0	0	0	0	0	0	0	0	0	0	1	1	1	0
**Discu<br>ssion<br>(4)**	**3**	**4**	**3**	**4**	**4**	**4**	**4**	**3**	**3**	**3**	**3**	**4**	**3**	**4**	**3**	**3**	**3**	**3**	**3**	**4**	**4**	**3**	**3**	**3**	**3**	**3**	**4**	**4**	**3**	**4**	**4**	**3**	**4**	**4**	**4**	**4**	**3**	**4**	**4**	**4**	**4**	**4**	**4**	**4**
23a	1	1	1	1	1	1	1	1	1	1	1	1	1	1	1	1	1	1	1	1	1	1	1	1	1	1	1	1	1	1	1	1	1	1	1	1	1	1	1	1	1	1	1	1
23b	1	1	1	1	1	1	1	1	1	1	1	1	1	1	1	1	1	1	1	1	1	1	1	1	1	1	1	1	1	1	1	1	1	1	1	1	1	1	1	1	1	1	1	1
23c	0	1	0	1	1	1	1	0	0	0	0	1	0	1	0	0	0	0	0	1	1	0	0	0	0	0	1	1	0	1	1	0	1	1	1	1	0	1	1	1	1	1	1	1
23d	1	1	1	1	1	1	1	1	1	1	1	1	1	1	1	1	1	1	1	1	1	1	1	1	1	1	1	1	1	1	1	1	1	1	1	1	1	1	1	1	1	1	1	1
**Other<br>Inform<br>ation<br>(6)**	**6**	**1**	**0**	**0**	**1**	**6**	**2**	**5**	**1**	**1**	**5**	**4**	**1**	**2**	**2**	**1**	**5**	**1**	**2**	**2**	**4**	**5**	**4**	**5**	**4**	**4**	**2**	**4**	**6**	**6**	**1**	**1**	**2**	**1**	**6**	**2**	**5**	**2**	**5**	**4**	**4**	**5**	**6**	**1**
24a	1	0	0	0	0	1	0	1	0	0	1	1	0	0	0	0	1	0	0	0	1	1	1	1	1	1	0	1	1	1	0	0	0	0	1	0	1	0	1	1	1	1	1	0
24b	1	0	0	0	0	1	0	1	0	0	1	1	0	0	0	0	1	0	0	0	1	1	1	1	1	1	0	1	1	1	0	0	0	0	1	0	1	0	1	1	1	1	1	0
24c	1	0	0	0	0	1	0	1	0	0	1	1	0	0	0	0	1	0	0	0	1	1	1	1	1	1	0	1	1	1	0	0	0	0	1	0	1	0	1	1	1	1	1	0
25	1	0	0	0	0	1	0	0	0	0	0	0	0	1	0	0	0	0	1	1	0	1	0	0	0	0	0	0	1	1	0	0	0	0	1	0	0	0	0	0	0	0	1	0
26	1	1	0	0	1	1	1	1	1	1	1	1	1	1	1	1	1	1	1	1	1	1	1	1	1	1	1	1	1	1	1	1	1	1	1	1	1	1	1	1	1	1	1	1
27	1	0	0	0	0	1	1	1	0	0	1	0	0	0	1	0	1	0	0	0	0	0	0	1	0	0	1	0	1	1	0	0	1	0	1	1	1	1	1	0	0	1	1	0
**Overa<br>ll**	3<br>9	3<br>2	2<br>7	3<br>1	3<br>5	3<br>5	2<br>7	2<br>9	2<br>5	2<br>7	3<br>3	3<br>3	3<br>1	3<br>5	2<br>9	3<br>2	3<br>6	2<br>5	2<br>9	3<br>0	3<br>3	4<br>1	3<br>5	4<br>0	3<br>7	3<br>5	3<br>0	4<br>0	3<br>1	3<br>8	3<br>6	3<br>0	3<br>4	2<br>9	3<br>9	3<br>3	4<br>0	3<br>5	4<br>3	3<br>7	3<br>9	3<br>8	4<br>1	3<br>1

Note: Overall = PRISMA 2022 for abstract + PRISMA 2022 checklist.

Supplementary Table S10 AMSTAR-2 ratings of included systematic reviews

AMSTAR-2 question	Bedrikovetski2021A	Bedrikovetski2021B	Bhandari2021	Cao2022	Castaldo2021	Chen2021	Cleere2022	Davey2021A	Davey2021B	Davey2021C	Deantonio2022	Gao2022A	Gao2022B	Han2022	Huang2020	Huang2021	Jia2022	Kao2021A	Kao2021B	Kao2022	Kothari2021	Kozikowski2021	Lee2022	Li2022A	Li2022B	Li2022C	Li2022D	Liang2022	Muhlbauer2021	Pesapane2022	Ren2022	Sha2022	Sohn2020	Ugga2021	Ursprung2020	Yang2022	Zhang2022A	Zhang2022B	Zhang2022C	Zhong2021	Zhong2022A	Zhong2022B	Zhong2022C	Zhong2022D
1	Y	Y	Y	Y	Y	Y	Y	Y	Y	Y	Y	Y	Y	Y	Y	Y	Y	Y	Y	Y	Y	Y	Y	Y	Y	Y	Y	Y	Y	Y	Y	Y	Y	Y	Y	Y	Y	Y	Y	Y	Y	Y	Y	Y
2	PY	N	N	PY	PY	PY	N	PY	N	N	PY	PY	PY	PY	N	PY	Y	N	N	N	PY	Y	Y	Y	Y	Y	N	Y	PY	N	PY	PY	PY	N	PY	PY	Y	PY	Y	PY	PY	PY	PY	PY
3	Y	Y	N	Y	N	Y	Y	Y	Y	Y	N	Y	N	Y	N	N	N	N	Y	Y	N	Y	Y	Y	Y	Y	N	Y	Y	Y	Y	Y	Y	Y	Y	Y	Y	Y	Y	Y	Y	Y	Y	Y
4	PY	PY	PY	N	N	N	N	N	N	N	PY	PY	N	PY	N	N	PY	N	N	N	PY	PY	N	PY	N	N	N	N	PY	PY	N	N	PY	N	N	N	PY	N	PY	PY	PY	PY	PY	N
5	Y	Y	Y	N	Y	Y	Y	Y	Y	Y	Y	Y	Y	Y	Y	N	N	N	N	Y	Y	Y	Y	Y	Y	Y	Y	Y	Y	Y	Y	Y	N	Y	Y	Y	N	Y	Y	Y	Y	Y	Y	Y
6	N	N	N	Y	Y	Y	N	N	N	Y	Y	N	Y	Y	N	Y	N	N	N	N	Y	Y	Y	Y	Y	Y	Y	Y	Y	Y	Y	Y	N	N	Y	Y	N	N	Y	Y	Y	Y	Y	N
7	N	N	N	N	N	N	N	N	N	N	N	N	N	N	N	N	N	N	N	N	N	N	N	N	N	Y	N	N	N	N	N	N	N	N	N	N	N	N	N	N	N	N	N	N
8	PY	PY	PY	PY	PY	PY	PY	PY	PY	PY	PY	PY	PY	PY	PY	PY	PY	PY	PY	PY	PY	PY	PY	PY	PY	PY	PY	PY	PY	PY	PY	PY	PY	PY	PY	PY	PY	PY	PY	PY	PY	PY	PY	PY
9	Y	Y	Y	Y	Y	N	N	N	N	N	N	Y	Y	Y	Y	Y	Y	N	N	Y	N	Y	N	Y	Y	Y	Y	Y	N	N	Y	Y	Y	Y	Y	Y	Y	Y	Y	Y	Y	Y	Y	Y
10	N	N	N	N	N	N	N	N	N	N	N	N	N	N	N	N	N	N	N	N	N	N	N	N	N	N	N	N	N	N	N	N	N	N	Y	N	N	N	N	N	N	N	N	N
11	N	N	N	Y	Y	N	N	N	N	N	N	N	Y	Y	N	Y	Y	N	N	N	N	Y	Y	Y	Y	Y	N	Y	N	Y	Y	Y	Y	N	N	Y	Y	Y	Y	N	N	N	N	Y
12	N	N	N	N	N	N	N	N	N	N	N	N	N	N	N	N	N	N	N	N	N	N	N	N	N	N	N	N	N	N	N	N	N	N	N	N	Y	N	N	N	N	N	N	N
13	Y	Y	Y	N	Y	N	Y	N	N	N	N	N	Y	N	Y	N	N	N	N	N	N	Y	N	Y	Y	Y	N	Y	Y	N	Y	Y	N	Y	Y	Y	Y	Y	Y	N	N	N	N	N
14	N	N	N	Y	Y	N	N	N	N	N	N	Y	Y	Y	N	Y	Y	N	N	N	N	Y	Y	Y	Y	Y	Y	Y	N	Y	Y	Y	Y	N	N	Y	Y	Y	Y	N	N	N	N	Y
15	Y	Y	N	Y	Y	N	Y	N	N	N	Y	Y	N	Y	N	Y	Y	Y	Y	Y	N	Y	Y	Y	Y	Y	N	Y	N	Y	Y	Y	Y	Y	Y	Y	Y	Y	Y	Y	Y	Y	Y	Y
16	Y	Y	N	N	Y	Y	Y	Y	Y	Y	Y	Y	Y	Y	Y	Y	Y	Y	Y	Y	Y	Y	Y	Y	Y	Y	Y	Y	Y	Y	Y	Y	Y	Y	Y	Y	Y	Y	Y	Y	Y	Y	Y	Y
Overall	CL	CL	CL	CL	CL	CL	CL	CL	CL	CL	CL	CL	CL	CL	CL	CL	CL	CL	CL	CL	CL	L	CL	L	CL	L	CL	CL	CL	CL	CL	CL	CL	CL	CL	CL	L	CL	L	CL	CL	CL	CL	CL

Note: Y = Yes, PY = partial yes, N = no. H = high confidence, M = moderate confidence, L = low confidence, CL = critically low confidence.

Supplementary Table S11 ROBIS tool assessments of included systematic reviews

ROBIS tool	Bedrikovetski2021A	Bedrikovetski2021B	Bhandari2021	Cao2022	Castaldo2021	Chen2021	Cleere2022	Davey2021A	Davey2021B	Davey2021C	Deantonio2022	Gao2022A	Gao2022B	Han2022	Huang2020	Huang2021	Jia2022	Kao2021A	Kao2021B	Kao2022	Kothari2021	Kozikowski2021
SQ #1.1	Y	PY	PY	PY	PY	Y	PY	Y	PY	PY	Y	Y	PY	PY	PY	PY	Y	PY	PY	PY	Y	Y
SQ #1.2	PY	PY	PY	PY	PY	PY	PY	PY	PY	PY	PY	PY	PY	PY	PY	PY	PY	PY	PY	PY	PY	PY
SQ #1.3	PY	PY	PY	PN	PN	PN	PN	PN	PN	PN	PY	PY	PN	PY	PN	PN	PY	PN	PN	PN	PY	PY
SQ #1.4	Y	Y	PY	Y	PY	Y	Y	Y	Y	Y	PY	Y	PY	Y	PY	PY	PY	PY	Y	Y	PY	Y
SQ #1.5	Y	Y	Y	PY	PY	PY	PY	PY	PY	PY	Y	Y	PY	PY	PY	PY	Y	PY	PY	PY	Y	Y
Domain 1	L	L	L	U	U	U	U	U	U	U	L	L	U	L	U	U	L	U	U	U	L	L
SQ #2.1	Y	Y	Y	PY	PY	PY	PY	PY	PY	PY	Y	Y	PY	Y	PY	PY	Y	PY	PY	PY	Y	Y
SQ #2.2	PY	PY	PY	PN	PN	PN	PN	PN	PN	PN	PY	PY	PN	PY	PN	PN	PY	PN	PN	PN	PY	PY
SQ #2.3	Y	Y	Y	PN	PN	PN	PN	PN	PN	PN	Y	Y	PN	Y	PN	PN	Y	PN	PN	PN	Y	Y
SQ #2.4	PY	PY	PY	PY	PY	PY	PY	PY	PY	PY	PY	PY	PY	PY	PY	PY	PY	PY	PY	PY	PY	PY
SQ #2.5	Y	Y	Y	PN	Y	Y	Y	Y	Y	Y	Y	Y	Y	Y	Y	PN	PN	PN	PN	Y	Y	Y
Domain 2	L	L	L	H	U	U	U	U	U	U	L	L	U	L	U	H	U	H	H	U	L	L
SQ #3.1	PN	PN	PN	Y	Y	Y	PN	PN	PN	Y	Y	PN	Y	Y	PN	Y	PN	PN	PN	PN	Y	Y
SQ #3.2	Y	PY	PY	PY	PY	Y	Y	Y	PY	PY	Y	PY	PY	PY	Y	PY	Y	PY	PY	PY	PY	PY
SQ #3.3	Y	Y	PY	PY	Y	Y	PY	PY	Y	Y	Y	Y	Y	Y	PY	Y	PN	PY	PY	PY	Y	Y
SQ #3.4	Y	Y	Y	Y	Y	N	N	N	N	N	N	Y	Y	Y	Y	Y	Y	N	N	Y	N	Y
SQ #3.5	Y	Y	Y	Y	Y	Y	PN	PN	PN	Y	Y	Y	Y	Y	PN	PN	Y	PN	Y	Y	PN	Y
Domain 3	U	U	U	L	L	H	H	H	H	H	H	U	L	L	U	U	U	H	H	U	H	L
SQ #4.1	Y	Y	PY	PY	Y	Y	PY	PY	Y	Y	Y	Y	Y	Y	Y	Y	PY	Y	Y	Y	Y	Y

ROBIS tool	Lee2022	Li2022A	Li2022B	Li2022C	Li2022D	Liang2022	Muhlbauer2021	Pesapane2022	Ren2022	Sha2022	Sohn2020	Ugga2021	Ursprung2020	Yang2022	Zhang2022A	Zhang2022B	Zhang2022C	Zhong2021	Zhong2022A	Zhong2022B	Zhong2022C	Zhong2022D
SQ #1.1	Y	Y	Y	Y	PY	Y	Y	Y	PY	PY	PY	PY	Y	PY	Y	PY	Y	Y	Y	Y	Y	PY
SQ #1.2	PY	PY	PY	PY	PY	PY	PY	PY	PY	PY	PY	PY	PY	PY	PY	PY	PY	PY	PY	PY	PY	PY
SQ #1.3	PN	PY	PN	PN	PN	PN	PY	PY	PN	PN	PY	PN	PN	PN	PY	PN	PY	PY	PY	PY	PY	PN
SQ #1.4	Y	Y	Y	Y	PY	Y	Y	Y	Y	Y	Y	Y	Y	Y	Y	Y	Y	Y	Y	Y	Y	Y
SQ #1.5	PY	Y	PY	PY	PY	PY	Y	Y	PY	PY	Y	PY	PY	PY	Y	PY	Y	Y	Y	Y	Y	PY
Domain 1	U	L	U	U	U	U	L	L	U	U	L	U	U	U	L	U	L	L	L	L	L	U
SQ #2.1	PY	Y	PY	PY	PY	PY	Y	Y	PY	PY	Y	PY	PY	PY	Y	PY	Y	Y	Y	Y	Y	PY
SQ #2.2	PN	PY	PN	PN	PN	PN	PY	PN	PN	PN	PY	PN	PN	PN	PY	PN	PN	PY	PY	PY	PY	PN
SQ #2.3	PN	Y	PN	PN	PN	PN	Y	Y	PN	PN	Y	PN	PN	PN	Y	PN	Y	Y	Y	Y	Y	PN
SQ #2.4	PY	PY	PY	PY	PY	PY	PY	PY	PY	PY	PY	PY	PY	PY	PY	PY	PY	PY	PY	PY	PY	PY
SQ #2.5	Y	Y	Y	Y	Y	Y	Y	Y	Y	Y	PN	Y	Y	Y	PN	Y	Y	Y	Y	Y	Y	Y
Domain 2	U	L	U	U	U	U	L	U	U	U	U	U	U	U	U	U	U	L	L	L	L	U
SQ #3.1	Y	Y	Y	Y	Y	Y	Y	Y	Y	Y	PN	PN	Y	Y	PN	PN	Y	Y	Y	Y	Y	PN
SQ #3.2	PY	Y	PY	PY	Y	PY	Y	Y	PY	PY	Y	PY	Y	Y	Y	Y	Y	PY	PY	Y	Y	PY
SQ #3.3	Y	PY	PN	PY	PN	PY	Y	Y	Y	PY	Y	PY	Y	Y	PY	PN	Y	Y	Y	Y	Y	Y
SQ #3.4	N	Y	Y	Y	Y	Y	N	N	Y	Y	Y	Y	Y	Y	Y	Y	Y	Y	Y	Y	Y	Y
SQ #3.5	Y	Y	Y	Y	PN	Y	PN	Y	Y	Y	Y	Y	Y	PN	Y	Y	Y	Y	Y	Y	Y	PN
Domain 3	H	L	U	L	U	L	H	H	L	L	U	U	L	U	U	U	L	L	L	L	L	U
SQ #4.1	Y	Y	PY	PY	PY	Y	Y	Y	Y	Y	Y	PY	Y	Y	Y	PY	Y	Y	Y	Y	Y	Y

SQ #4.2	PY	PY	N	PY	PY	PY	PY	PY	N	N	PY	N	PY	PY	N	N	PY	PY	PY	PY	PY	PY	PY	PY	PY	PY	N	PY	PY	PY	PY	PY	PY	PY	PY	PY	PY	PY	PY	PY	PY	PY	PY	PY
SQ #4.3	PY	PY	PY	Y	PY	PY	PY	PY	PY	PY	PY	PY	Y	PY	PY	PY	PY	PY	PY	PY	Y	Y	PY	PY	Y	PY	PY	Y	PY	PY	Y	PY	PY	Y	Y	PY	PY	Y	PY	Y	Y	Y	Y	PY
SQ #4.4	N	N	N	Y	Y	N	N	N	N	N	N	N	Y	Y	N	Y	Y	N	N	N	N	Y	Y	Y	Y	Y	N	Y	N	Y	Y	Y	Y	N	N	Y	Y	Y	Y	N	N	N	N	Y
SQ #4.5	Y	Y	Y	Y	Y	N	Y	N	Y	Y	Y	Y	N	Y	N	Y	Y	Y	Y	Y	N	Y	Y	Y	Y	Y	N	Y	N	Y	Y	Y	Y	Y	Y	Y	Y	Y	Y	Y	Y	Y	Y	Y
SQ #4.6	PN	PN	PN	PY	PN	N	N	N	N	N	N	PN	PN	PN	PN	PN	PN	N	N	PN	N	PN	N	PN	PY	PN	PN	PY	N	N	PN	PN	PN	PN	PN	PN	PY	PY	PY	PN	PN	PN	PN	PN
Domain 4	H	H	H	L	U	H	H	H	H	H	H	H	H	U	H	H	U	H	H	H	H	U	H	U	L	U	H	L	H	H	U	U	U	H	H	U	L	L	L	H	H	H	H	U
SQ #A	N	N	N	N	PY	N	N	N	N	N	N	N	N	PY	N	N	PY	N	N	N	N	PY	N	PY	PY	PY	N	PY	N	N	PY	PY	PY	N	N	PY	PY	PY	PY	N	N	N	N	PY
SQ #B	Y	Y	Y	Y	Y	PY	PY	PY	PY	PY	PY	Y	Y	Y	Y	Y	Y	PY	PY	Y	PY	Y	PY	Y	Y	Y	Y	Y	PY	PY	Y	Y	Y	Y	Y	Y	Y	Y	Y	Y	Y	Y	Y	Y
SQ #C	PY	PY	PY	Y	PY	PY	PY	PY	PY	PY	PY	PY	PY	PY	PY	Y	PY	PY	PY	PY	PY	PY	PY	PY	Y	PY	PY	Y	PY	Y	PY	PY	PY	PY	PY	PY	Y	Y	Y	PY	PY	PY	PY	PY
Overall	H	H	H	H	U	H	H	H	H	H	H	H	H	U	H	H	U	H	H	H	H	U	H	U	L	U	H	L	H	H	U	U	U	H	H	U	L	L	L	H	H	H	H	U

Note: SQ = signaling question. Y = Yes, PY = probably yes, PN = probably no, N = no. H = high risk of bias, U = unclear risk of bias, L = low risk of bias.

PROSPERO
International prospective register of systematic reviews

National Institute for Health Research

UNIVERSITY of York
Centre for Reviews and Dissemination

Systematic review

1. * Review title.

Give the title of the review in English

Study Quality and Clinical Value of Radiomics: An Overview of Systematic Reviews and Meta-analyzes of Radiomics Studies

2. Original language title.

For reviews in languages other than English, give the title in the original language. This will be displayed with the English language title.

3. * Anticipated or actual start date.

Give the date the systematic review started or is expected to start.

01/08/2021

4. * Anticipated completion date.

Give the date by which the review is expected to be completed.

31/12/2021

5. * Stage of review at time of this submission.

This field uses answers to initial screening questions. It cannot be edited until after registration.

Tick the boxes to show which review tasks have been started and which have been completed.

Update this field each time any amendments are made to a published record.

The review has not yet started: No

Review stage	Started	Completed
Preliminary searches	Yes	No
Piloting of the study selection process	No	No
Formal screening of search results against eligibility criteria	No	No
Data extraction	No	No
Risk of bias (quality) assessment	No	No
Data analysis	No	No

Provide any other relevant information about the stage of the review here.

6. * Named contact.

The named contact is the guarantor for the accuracy of the information in the register record. This may be any member of the review team.

Jingyu Zhong

Email salutation (e.g. "Dr Smith" or "Joanne") for correspondence:

Dr Jingyu Zhong

7. * Named contact email.

Give the electronic email address of the named contact.

wal_zjy@163.com

8. Named contact address

Give the full institutional/organisational postal address for the named contact.

No. 1111, Xianxia Rd., Changning District, Shanghai 200050, China

9. Named contact phone number.

Give the telephone number for the named contact, including international dialling code.

15221992711

10. * Organisational affiliation of the review.

Full title of the organisational affiliations for this review and website address if available. This field may be completed as 'None' if the review is not affiliated to any organisation.

Department of Imaging, Tongren Hospital, Shanghai Jiao Tong University School of Medicine

Organisation web address:

https://www.shtrhospital.com

11. * Review team members and their organisational affiliations.

Give the personal details and the organisational affiliations of each member of the review team. Affiliation refers to groups or organisations to which review team members belong. **NOTE: email and country now MUST be entered for each person, unless you are amending a published record.**

Dr Jingyu Zhong. Department of Imaging, Tongren Hospital, Shanghai Jiao Tong University School of Medicine
Dr Yangfan Hu. Department of Imaging, Tongren Hospital, Shanghai Jiao Tong University School of Medicine
Professor Weiwu Yao. Department of Imaging, Tongren Hospital, Shanghai Jiao Tong University School of Medicine

12. * Funding sources/sponsors.

Details of the individuals, organizations, groups, companies or other legal entities who have funded or sponsored the review.

This work is supported by the National Natural Science Foundation of China (81771790) and the Medicine and Engineering Combination Project of Shanghai Jiao Tong University (YG2019ZDB09).

Grant number(s)

State the funder, grant or award number and the date of award

13. * Conflicts of interest.

List actual or perceived conflicts of interest (financial or academic).

None

14. Collaborators.

Give the name and affiliation of any individuals or organisations who are working on the review but who are not listed as review team members. **NOTE: email and country must be completed for each person, unless you are amending a published record.**

15. * Review question.

State the review question(s) clearly and precisely. It may be appropriate to break very broad questions down into a series of related more specific questions. Questions may be framed or refined using PI(E)COS or similar where relevant.

What is the existing systematic review evidence for (1) the diagnostic, prognostic, and predictive accuracy of radiomics studies in clinical settings, and (2) quality assessment and risk of bias tools for these studies?

16. * Searches.

State the sources that will be searched (e.g. Medline). Give the search dates, and any restrictions (e.g. language or publication date). Do NOT enter the full search strategy (it may be provided as a link or attachment below.)

We will search the following databases for eligible systematic reviews: (1) Cochrane Database of Systematic Reviews (CDSR), (2) PubMed, (3) EMBASE, and (4) Web of Science. Systematic reviews concerning radiomics studies Literature search strategies will be developed using medical subject headings (MeSH) and derived words, including "radiomics", "systematic review", and "meta-analysis". Only systematic reviews published after Jan 01 2012 will be included, since the term "radiomics" was first coined in early 2012. The search strategy will

include only terms relating to the review question. Publications must be available in English, Chinese, Japanese, German or French. To ensure literature saturation, we will check the reference lists of included systematic reviews identified through the search.

17. URL to search strategy.

Upload a file with your search strategy, or an example of a search strategy for a specific database, (including the keywords) in pdf or word format. In doing so you are consenting to the file being made publicly accessible. Or provide a URL or link to the strategy. Do NOT provide links to your search **results**.

https://www.crd.york.ac.uk/PROSPEROFILES/272746_STRATEGY_20210810.pdf

Alternatively, upload your search strategy to CRD in pdf format. Please note that by doing so you are consenting to the file being made publicly accessible.

Do not make this file publicly available until the review is complete

18. * Condition or domain being studied.

Give a short description of the disease, condition or healthcare domain being studied in your systematic review.

Since Lambin et al first coined the term radiomics in early 2012, almost a decade has passed. Thousands of papers have been published in this rapidly evolving field. However, what is the existing systematic review evidence for (1) the diagnostic, prognostic, and predictive accuracy of radiomics studies in clinical settings, and (2) quality assessment and risk of bias tools for these studies, is unknown. Therefore, we aim to summarize current evidence from radiomics studies, and to help improve the quality of such studies.

19. * Participants/population.

Specify the participants or populations being studied in the review. The preferred format includes details of both inclusion and exclusion criteria.

Patients or participants assessed by radiomics approaches for clinical questions, typically diagnostic, prognostic, and predictive accuracy tests.

20. * Intervention(s), exposure(s).

Give full and clear descriptions or definitions of the interventions or the exposures to be reviewed. The preferred format includes details of both inclusion and exclusion criteria.

Diagnostic test accuracy of radiomics approaches used for clinical questions.

21. * Comparator(s)/control.

Where relevant, give details of the alternatives against which the intervention/exposure will be compared (e.g. another intervention or a non-exposed control group). The preferred format includes details of both inclusion and exclusion criteria.

Not applicable.

22. * Types of study to be included.

Give details of the study designs (e.g. RCT) that are eligible for inclusion in the review. The preferred format includes both inclusion and exclusion criteria. If there are no restrictions on the types of study, this should be

stated.

Systematic reviews and meta-analyzes of diagnostic test accuracy.

23. Context.

Give summary details of the setting or other relevant characteristics, which help define the inclusion or exclusion criteria.

Systematic reviews concerning diagnostic test accuracy of radiomics approaches will be included. Meta-analyzes focusing on a specific clinical question will be included.

24. * Main outcome(s).

Give the pre-specified main (most important) outcomes of the review, including details of how the outcome is defined and measured and when these measurement are made, if these are part of the review inclusion criteria.

The bibliographic information, review questions, and quality and risk of bias assessment tools used in systematic reviews will be summarized. The rating of AMSTAR 2 tool, PRISMA 2020 checklist, and ROBIS tool per systematic review will be used as metrics of methodological quality, reporting quality, and risk of bias, respectively. If meta-analyzes concerning a specific clinical question were performed in included systematics reviews, statistical analyses for the evidence rating will conducted for levels of supporting evidence of meta-analyzes.

Measures of effect

Please specify the effect measure(s) for you main outcome(s) e.g. relative risks, odds ratios, risk difference, and/or 'number needed to treat.

Levels of supporting evidence of will be categorized into five levels: convincing, highly suggestive, suggestive, weak, and not suggestive based on the criteria shown as follows: p 10?6, 1000 events, the largest study reaches statistical significance (p 0.05), I^2 50%, the null value excluded by the 95% PI, no small-study effects (p 0.1 for Egger’s test) and excess significance (p 0.1), and survived the 10% credibility ceiling (p 0.05) for strong evidence; p 10?6, 1000 events, the largest study reaches statistical significance (p 0.05) for highly suggestive evidence; p 10?3, 1000 events for suggestive evidence; p 0.05 for weak evidence; and p 0.05 for not suggestive evidence.

25. * Additional outcome(s).

List the pre-specified additional outcomes of the review, with a similar level of detail to that required for main outcomes. Where there are no additional outcomes please state ‘None’ or ‘Not applicable’ as appropriate to the review

None.

Measures of effect

Please specify the effect measure(s) for you additional outcome(s) e.g. relative risks, odds ratios, risk difference, and/or 'number needed to treat.

Not applicable.

NHS
National Institute for
Health Research

26. * Data extraction (selection and coding).

Describe how studies will be selected for inclusion. State what data will be extracted or obtained. State how this will be done and recorded.

All data will be managed using the latest version of EndNote software. The first 15 sources will be pilot title and abstract screened by two reviewers according to the inclusion and exclusion criteria. Subsequent discussion will inform the screening notes. Title/abstract screening and full text screening will be conducted by two reviewers. A third reviewer will resolve any disagreements.

A data extraction tool will be established and then trialed on two randomly chosen studies, which fulfilled all the inclusion criteria. These shall be used to train reviewers to appropriately apply the data extraction tool. Data will be abstracted by one reviewer and spot-checked="checked" value="1" by a second, with a third reviewer providing moderation as required. Key data extracted will include characteristics of included systematic reviews: (1) bibliographic information, (2) review questions, (3) quality and risk of bias assessment tools used, and (4) metrics of meta-analyzes.

27. * Risk of bias (quality) assessment.

State which characteristics of the studies will be assessed and/or any formal risk of bias/quality assessment tools that will be used.

AMSTAR 2 tool, PRISMA 2020 checklist, and ROBIS tool will be used for methodological quality, reporting quality, and risk of bias, respectively.

28. * Strategy for data synthesis.

Describe the methods you plan to use to synthesise data. This **must not be generic text** but should be **specific to your review** and describe how the proposed approach will be applied to your data. If meta-analysis is planned, describe the models to be used, methods to explore statistical heterogeneity, and software package to be used.

A narrative synthesis will be provided with information presented in the text and/or tables to summarize and explain the characteristics and findings of the included studies. A quantitative synthesis will be done if the included studies are sufficiently homogenous. Additional analyses may be possible for comparing across included reviews, in which case we will consult with a statistical specialist regarding the validity and suitability of further analyses. All analysis will be based on aggregate data.

29. * Analysis of subgroups or subsets.

State any planned investigation of ‘subgroups’. Be clear and specific about which type of study or participant will be included in each group or covariate investigated. State the planned analytic approach.

Not planned.

30. * Type and method of review.

Select the type of review, review method and health area from the lists below.

Type of review

Cost effectiveness
No

Diagnostic
Yes

Epidemiologic
No

Individual patient data (IPD) meta-analysis
No

Intervention
No

Living systematic review
No

Meta-analysis
Yes

Methodology
No

Narrative synthesis
Yes

Network meta-analysis
No

Pre-clinical
No

Prevention
No

Prognostic
Yes

Prospective meta-analysis (PMA)
No

Review of reviews
Yes

Service delivery
No

Synthesis of qualitative studies
No

Systematic review
Yes

Other
No

Health area of the review

Alcohol/substance misuse/abuse
No

Blood and immune system
No

Cancer

No

Cardiovascular
No

Care of the elderly
No

Child health
No

Complementary therapies
No

COVID-19
No

Crime and justice
No

Dental
No

Digestive system
No

Ear, nose and throat
No

Education
No

Endocrine and metabolic disorders
No

Eye disorders
No

General interest
Yes

Genetics
No

Health inequalities/health equity
No

Infections and infestations
No

International development
No

Mental health and behavioural conditions
No

Musculoskeletal
No

Neurological
No

Nursing
No

Obstetrics and gynaecology
No

Oral health
No

Palliative care
No

Perioperative care
No

Physiotherapy
No

Pregnancy and childbirth
No

Public health (including social determinants of health)
No

Rehabilitation
No

Respiratory disorders
No

Service delivery
No

Skin disorders
No

Social care
No

Surgery
No

Tropical Medicine
No

Urological
No

Wounds, injuries and accidents
No

Violence and abuse
No

31. Language.

Select each language individually to add it to the list below, use the bin icon to remove any added in error.
English

There is not an English language summary

32. * Country.

Select the country in which the review is being carried out. For multi-national collaborations select all the countries involved.

China

33. Other registration details.

Name any other organisation where the systematic review title or protocol is registered (e.g. Campbell, or The Joanna Briggs Institute) together with any unique identification number assigned by them. If extracted data will be stored and made available through a repository such as the Systematic Review Data Repository (SRDR), details and a link should be included here. If none, leave blank.

34. Reference and/or URL for published protocol.

If the protocol for this review is published provide details (authors, title and journal details, preferably in Vancouver format)

Add web link to the published protocol.

Or, upload your published protocol here in pdf format. Note that the upload will be publicly accessible.

No I do not make this file publicly available until the review is complete

Please note that the information required in the PROSPERO registration form must be completed in full even if access to a protocol is given.

35. Dissemination plans.

Do you intend to publish the review on completion?

Yes

Give brief details of plans for communicating review findings.?

36. Keywords.

Give words or phrases that best describe the review. Separate keywords with a semicolon or new line. Keywords help PROSPERO users find your review (keywords do not appear in the public record but are included in searches). Be as specific and precise as possible. Avoid acronyms and abbreviations unless these are in wide use.

Radiomics, Overview of Reviews

37. Details of any existing review of the same topic by the same authors.

If you are registering an update of an existing review give details of the earlier versions and include a full bibliographic reference, if available.

38. * Current review status.

Update review status when the review is completed and when it is published.New registrations must be ongoing so this field is not editable for initial submission.
Please provide anticipated publication date

Review_Ongoing

39. Any additional information.

Provide any other information relevant to the registration of this review.

References

Lambin P, Rios-Velazquez E, Leijenaar R, Carvalho S, van Stiphout RG, Granton P, Zegers CM, Gillies R, Boellard R, Dekker A, Aerts HJ. Radiomics: extracting more information from medical images using

advanced feature analysis. Eur J Cancer. 2012 Mar;48(4):441-6. doi: 10.1016/j.ejca.2011.11.036. Epub 2012 Jan 16. PMID: 22257792; PMCID: PMC4533986.

2. Song J, Yin Y, Wang H, Chang Z, Liu Z, Cui L. A review of original articles published in the emerging field of radiomics. Eur J Radiol. 2020 Jun;127:108991. doi: 10.1016/j.ejrad.2020.108991. Epub 2020 Apr 12. PMID: 32334372.

3. van Timmeren JE, Cester D, Tanadini-Lang S, Alkadhi H, Baessler B. Radiomics in medical imaging-"how-to" guide and critical reflection. Insights Imaging. 2020 Aug 12;11(1):91. doi: 10.1186/s13244-020-00887-2. PMID: 32785796; PMCID: PMC7423816.

4. Halligan S, Menu Y, Mallett S. Why did European Radiology reject my radiomic biomarker paper? How to correctly evaluate imaging biomarkers in a clinical setting. Eur Radiol. 2021 May 18. doi: 10.1007/s00330-021-07971-1. Epub ahead of print. PMID: 34003349.

5. Pollock A, Campbell P, Brunton G, Hunt H, Estcourt L. Selecting and implementing overview methods: implications from five exemplar overviews. Syst Rev. 2017 Jul 18;6(1):145. doi: 10.1186/s13643-017-0534-3. PMID: 28720141; PMCID: PMC5516331.

6. Pollock M, Fernandes RM, Pieper D, Tricco AC, Gates M, Gates A, Hartling L. Preferred Reporting Items for Overviews of Reviews (PRIOR): a protocol for development of a reporting guideline for overviews of reviews of healthcare interventions. Syst Rev. 2019 Dec 23;8(1):335. doi: 10.1186/s13643-019-1252-9. PMID: 31870434; PMCID: PMC6929355.

7. Gates M, Gates A, Guitard S, Pollock M, Hartling L. Guidance for overviews of reviews continues to accumulate, but important challenges remain: a scoping review. Syst Rev. 2020 Nov 4;9(1):254. doi: 10.1186/s13643-020-01509-0. PMID: 33148319; PMCID: PMC7643411.

8. Shea BJ, Reeves BC, Wells G, Thuku M, Hamel C, Moran J, Moher D, Tugwell P, Welch V, Kristjansson E, Henry DA. AMSTAR 2: a critical appraisal tool for systematic reviews that include randomised or non-randomised studies of healthcare interventions, or both. BMJ. 2017 Sep 21;358:j4008. doi: 10.1136/bmj.j4008. PMID: 28935701; PMCID: PMC5833365.

9. Page MJ, McKenzie JE, Bossuyt PM, Boutron I, Hoffmann TC, Mulrow CD, Shamseer L, Tetzlaff JM, Akl EA, Brennan SE, Chou R, Glanville J, Grimshaw JM, Hróbjartsson A, Lalu MM, Li T, Loder EW, Mayo-Wilson E, McDonald S, McGuinness LA, Stewart LA, Thomas J, Tricco AC, Welch VA, Whiting P, Moher D. The PRISMA 2020 statement: an updated guideline for reporting systematic reviews. BMJ. 2021 Mar 29;372:n71. doi: 10.1136/bmj.n71. PMID: 33782057; PMCID: PMC8005924.

10. Whiting P, Savovi? J, Higgins JP, Caldwell DM, Reeves BC, Shea B, Davies P, Kleijnen J, Churchill R; ROBIS group. ROBIS: A new tool to assess risk of bias in systematic reviews was developed. J Clin Epidemiol. 2016 Jan;69:225-34. doi: 10.1016/j.jclinepi.2015.06.005. Epub 2015 Jun 16. PMID: 26092286; PMCID: PMC4687950.

11. Dang Y, Hou Y. The prognostic value of late gadolinium enhancement in heart diseases: an umbrella review of meta-analyses of observational studies. Eur Radiol. 2021 Jul;31(7):4528-4537. doi: 10.1007/s00330-020-07437-w. Epub 2021 Jan 7. PMID: 33409800.

40. Details of final report/publication(s) or preprints if available.

Leave empty until publication details are available OR you have a link to a preprint (NOTE: this field is not editable for initial submission). List authors, title and journal details preferably in Vancouver format.

Give the link to the published review or preprint.

Checklists

Title: An overview of meta-analyses on radiomics: more evidence is needed to support clinical translation

1. PRISMA 2020 checklist for abstract

TITLE	CHECKLIST ITEM	REPORTED ON PAGE #
1. Title:	Identify the report as a systematic review, meta-analysis, or both.	1
BACKGROUND		
2. Objectives:	The research question including components such as participants, interventions, comparators, and outcomes.	2
METHODS		
3. Eligibility criteria:	Study and report characteristics used as criteria for inclusion.	2
4. Information sources:	Key databases searched and search dates.	2
5. Risk of bias:	Methods of assessing risk of bias.	2
RESULTS		
6. Included studies:	Number and type of included studies and participants and relevant characteristics of studies.	2
7. Synthesis of results:	Results for main outcomes (benefits and harms), preferably indicating the number of studies and participants for each. If meta-analysis was done, include summary measures and confidence intervals.	2
8. Description of the effect:	Direction of the effect (i.e. which group is favoured) and size of the effect in terms meaningful to clinicians and patients.	2
DISCUSSION		
9. Strengths and Limitations of evidence:	Brief summary of strengths and limitations of evidence (e.g. inconsistency, imprecision, indirectness, or risk of bias, other supporting or conflicting evidence)	2
10. Interpretation:	General interpretation of the results and important implications	2
OTHER		
11. Funding:	Primary source of funding for the review.	Full title page
12. Registration:	Registration number and registry name.	Text

Note: Extracted from Page MJ, McKenzie JE, Bossuyt PM, Boutron I, Hoffmann TC, Mulrow CD, Shamseer L, Tetzlaff JM, Akl EA, Brennan SE, Chou R, Glanville J, Grimshaw JM, Hróbjartsson A, Lalu MM, Li T, Loder EW, Mayo-Wilson E, McDonald S, McGuinness LA, Stewart LA, Thomas J, Tricco AC, Welch VA, Whiting P, Moher D.

The PRISMA 2020 statement: an updated guideline for reporting systematic reviews. BMJ. 2021 Mar 29;372:n71. doi: 10.1136/bmj.n71. PMID: 33782057; PMCID: PMC8005924.

2. PRISMA 2020 checklist

**Section and Topic**	**Item #**	**Checklist item**	**Location where item is reported**
**TITLE**			
Title	1	Identify the report as a systematic review.	1
**ABSTRACT**			
Abstract	2	See the PRISMA 2020 for Abstracts checklist.	2
**INTRODUCTION**			
Rationale	3	Describe the rationale for the review in the context of existing knowledge.	3
Objectives	4	Provide an explicit statement of the objective(s) or question(s) the review addresses.	3
**METHODS**			
Eligibility criteria	5	Specify the inclusion and exclusion criteria for the review and how studies were grouped for the syntheses.	3-4
Information sources	6	Specify all databases, registers, websites, organisations, reference lists and other sources searched or consulted to identify studies. Specify the date when each source was last searched or consulted.	3
Search strategy	7	Present the full search strategies for all databases, registers and websites, including any filters and limits used.	3-4
Selection process	8	Specify the methods used to decide whether a study met the inclusion criteria of the review, including how many reviewers screened each record and each report retrieved, whether they worked independently, and if applicable, details of automation tools used in the process.	3-4
Data collection process	9	Specify the methods used to collect data from reports, including how many reviewers collected data from each report, whether they worked independently, any processes for obtaining or confirming data from study investigators, and if applicable, details of automation tools used in the process.	4
Data items	10a	List and define all outcomes for which data were sought. Specify whether all results that were compatible with each outcome domain in each study were sought (e.g. for all measures, time points, analyses), and if not, the methods used to decide which	4

		results to collect.	
	10b	List and define all other variables for which data were sought (e.g. participant and intervention characteristics, funding sources). Describe any assumptions made about any missing or unclear information.	4
Study risk of bias assessment	11	Specify the methods used to assess risk of bias in the included studies, including details of the tool(s) used, how many reviewers assessed each study and whether they worked independently, and if applicable, details of automation tools used in the process.	4
Effect measures	12	Specify for each outcome the effect measure(s) (e.g. risk ratio, mean difference) used in the synthesis or presentation of results.	4
Synthesis methods	13a	Describe the processes used to decide which studies were eligible for each synthesis (e.g. tabulating the study intervention characteristics and comparing against the planned groups for each synthesis (item #5)).	4
	13b	Describe any methods required to prepare the data for presentation or synthesis, such as handling of missing summary statistics, or data conversions.	4
	13c	Describe any methods used to tabulate or visually display results of individual studies and syntheses.	4
	13d	Describe any methods used to synthesize results and provide a rationale for the choice(s). If meta-analysis was performed, describe the model(s), method(s) to identify the presence and extent of statistical heterogeneity, and software package(s) used.	4
	13e	Describe any methods used to explore possible causes of heterogeneity among study results (e.g. subgroup analysis, meta-regression).	n/a
	13f	Describe any sensitivity analyses conducted to assess robustness of the synthesized results.	n/a
Reporting bias assessment	14	Describe any methods used to assess risk of bias due to missing results in a synthesis (arising from reporting biases).	4
Certainty assessment	15	Describe any methods used to assess certainty (or confidence) in the body of evidence for an outcome.	4
**RESULTS**			
Study selection	16a	Describe the results of the search and selection process, from the number of records identified in the search to the number of studies included in the review, ideally using a flow diagram.	4-5

	16b	Cite studies that might appear to meet the inclusion criteria, but which were excluded, and explain why they were excluded.	5
Study characteristics	17	Cite each included study and present its characteristics.	5
Risk of bias in studies	18	Present assessments of risk of bias for each included study.	5
Results of individual studies	19	For all outcomes, present, for each study: (a) summary statistics for each group (where appropriate) and (b) an effect estimate and its precision (e.g. confidence/credible interval), ideally using structured tables or plots.	5
Results of syntheses	20a	For each synthesis, briefly summarise the characteristics and risk of bias among contributing studies.	5
	20b	Present results of all statistical syntheses conducted. If meta-analysis was done, present for each the summary estimate and its precision (e.g. confidence/credible interval) and measures of statistical heterogeneity. If comparing groups, describe the direction of the effect.	5
	20c	Present results of all investigations of possible causes of heterogeneity among study results.	5
	20d	Present results of all sensitivity analyses conducted to assess the robustness of the synthesized results.	5
Reporting biases	21	Present assessments of risk of bias due to missing results (arising from reporting biases) for each synthesis assessed.	5
Certainty of evidence	22	Present assessments of certainty (or confidence) in the body of evidence for each outcome assessed.	5
DISCUSSION			
Discussion	23a	Provide a general interpretation of the results in the context of other evidence.	5-6
	23b	Discuss any limitations of the evidence included in the review.	6
	23c	Discuss any limitations of the review processes used.	6
	23d	Discuss implications of the results for practice, policy, and future research.	6-7
OTHER INFORMATION			
Registration and protocol	24a	Provide registration information for the review, including register name and registration number, or state that the review was not registered.	Supplementary Material

	24b	Indicate where the review protocol can be accessed, or state that a protocol was not prepared.	3
	24c	Describe and explain any amendments to information provided at registration or in the protocol.	3
Support	25	Describe sources of financial or non-financial support for the review, and the role of the funders or sponsors in the review.	Full title page
Competing interests	26	Declare any competing interests of review authors.	Full title page
Availability of data, code and other materials	27	Report which of the following are publicly available and where they can be found: template data collection forms; data extracted from included studies; data used for all analyses; analytic code; any other materials used in the review.	Full title page

Note: Extracted from Page MJ, McKenzie JE, Bossuyt PM, Boutron I, Hoffmann TC, Mulrow CD, Shamseer L, Tetzlaff JM, Akl EA, Brennan SE, Chou R, Glanville J, Grimshaw JM, Hróbjartsson A, Lalu MM, Li T, Loder EW, Mayo-Wilson E, McDonald S, McGuinness LA, Stewart LA, Thomas J, Tricco AC, Welch VA, Whiting P, Moher D. The PRISMA 2020 statement: an updated guideline for reporting systematic reviews. BMJ. 2021 Mar 29;372:n71. doi: 10.1136/bmj.n71. PMID: 33782057; PMCID: PMC8005924.

3. PRIO-harms checklist

**Section/topic**	**Item#**	**Checklist item**	**Reported on page#**
**Title**			
1.Tiltle	1a	Specify the study design with terms such as ‘overview of (systematic) reviews,’ ‘umbrella review,’ ‘(systematic) review of systematic reviews,’ or ‘(systematic) meta-review’ in the title of the OoSRs.	1
	1b	Mention ‘‘safety’’ or harms related terms, or the adverse event(s) of interest in the title of the OoSRs.	n/a
**Abstract**			
2. Structured-like summary	2a	Provide a structured-like abstract, as applicable: background, objective, data sources, selection criteria, data extraction, review appraisal, data synthesis methods, results, limitations, conclusions.	2
	2b	Report the main findings of analysis of harms undertaken in the OoSRs or/and in the included SRs.	n/a
**Introduction**			
3. Rationale	3a	Specify the rationale and the scope (wide or narrow agendas) for the overview in the context of an existing body of knowledge on the topic.	3
	3b	Provide a balanced presentation of potential benefits and harms of the intervention(s).	3

	3c	Define which events are considered harms according to previous literature and provide a clear rationale for the specific harms included in the OoSRs.	3
4. Objectives (PICOS)	4	Provide an explicit statement of research question(s) that specifies PICOS: Participants, Interventions, Comparators, Outcomes, Study design.	3
**Methods**			
5. Protocol and registration	5a	Indicate if a protocol exists or not.	3
	5b	If registered, provide the name of the registry (such as a valid Web address, PROSPERO).	3
6. Eligibility criteria and outcomes of interest	6a	Specify inclusion and exclusion criteria for study design, participants, interventions, and comparators in detail.	3-4
	6b	List (and define whenever it is necessary) the outcomes for which data were recorded, ideally include prioritization of main and additional outcomes.	4
	6c	Include adverse events as (primary or secondary) outcome of interest. Define them and grade their severity (such as mild, moderate, severe, fatal; severity could also be described in the appendix), if appropriate.	3-4
	6d	Specify report characteristics (such as language restrictions, publication status, and years considered) used as criteria for eligibility for the OoSRs (see also item 7).	3-4
7. Information sources	7a	Search at least two electronic databases.	3
	7b	Search supplementary sources (e.g., hand searching, reference lists, related reviews and guidelines, protocol registries, conference abstracts, and other gray literature).	3
	7c	Report the date of last search and/or dates of coverage for each database.	3
8. Search strategy	8a	Specify full electronic search strategy (algorithm) for at least one database including any limits used (e.g., language and date restrictionsdsee also subitems 6d and 7c) such that it could be repeated.	3-4
	8b	Present any additional search process (e.g., algorithm or filter for adverse events, searches in pertinent websites) specifically to identify adverse events that have been investigated.	3-4
9. Data management and selection process	9a	Describe the software that was used to manage records and data throughout the OoSRs.	4
	9b	Define what is an SR and provide the process for selecting SRs and its relevant details (screening the title and abstract or full text by at least two reviewers, selection by multiple independent investigators and resolving disagreements by consensus).	4
	9c	Report any attempt to handle overlapping (include one review among multiple potential candidates by choosing for example the most updated SR, the most methodologically rigorous SR or the SR with larger number of primary studies).	4

10. Additional search for primary studies	10	Report additional search to identify eligible primary studies (e.g., searching in more databases or update the search) and its relevant details.	3
11. Data collection process	11a	Describe the method of data extraction from included SRs (e.g., data collection form, extraction in duplicate and independently, resolving disagreements by consensus).	4
	11b	Report any processes for obtaining, confirming, or updating data from investigators (e.g., contact with authors of included reviews, obtain data from primary studies of included reviews).	4
12. Data items	12	List (and define whenever is necessary) the variables for which data were recorded (e.g., PICOS items, number of included studies and participants, dose, length of follow up, results, funding sources) and any data assumptions and simplifications made.	4
13. Assessment of methodological quality and quality of evidence	13a	State the evaluation of reporting or/and methodological quality (e.g., using PRISMA or PRISMA-harms, AMSTAR or R-AMSTAR) of the included reviews.	4
	13b	State the evaluation of quality for individual studies that are included in the SRs (inform whether tools such as Jadad or RoB of Cochrane were used by the included reviews) and for the additional primary studies.	4
	13c	State the evaluation of quality of evidence (e.g., using GRADE approach).	4
	13d	Describe the methods (e.g., piloted forms, independently, in duplicate) used for the quality assessment.	4
14. Meta-bias(es)	14	Specify any planned assessment of meta-bias(es) (such as publication bias or selective reporting across studies, ROBIS tool).	4
15. Data synthesis	15a	Specify clearly the method (narrative, meta-analysis, or network meta-analysis) of handling or synthesizing data and their details (e.g., state the principal summary measures that were extracted or calculated, how heterogeneity was assessed, what statistical approaches were used if a quantitative synthesis has been conducted)	4
	15b	Describe the software that was used to analyze the data if a quantitative synthesis has been conducted.	4
	15c	Report if zero events are included in the studies and how they were handled in statistical analyses, if relevant.	4
	15d	Describe methods of any prespecified additional analyses (such as sensitivity or subgroup analyses, meta-regression).	4
**Results**			

16. Review and primary study selection	16a	Provide the details of review selection (e.g., numbers of reviews screened, retrieved, and included and excluded in the overview) and the number of the additional eligible primary studies that were included, ideally with a flow diagram of the overview process.	4-5
	16b	Present a flow diagram that gives separately the number of studies focused on harms outcomes.	Figure 1
	16c	List the studies (full citation) that were excluded after reading the full text and provide reasons.	5
17. Review and primary study characteristics	17a	Describe characteristics of each included SR in tables (such as title or author, search date, PICOS, design and number of studies included, number and age range of participants, dose/frequency, follow up period [treatment duration], review limitations, results or conclusion) and of each additional primary study.	5, Table 1
	17b	For each included SR report language and publication status restrictions that have been used.	5
18. Overlapping	18	Present or/and discuss about overlapping of studies within SRs (at least one of the following):<br>➓ Present measures of overlap (such as CCA).<br>➓ Provide citation matrix.<br>➓ Give the number of index publications or/and discuss about overlapping.	Supplementary Material
19. Present assessment of methodological quality and quality of evidence	19	Present results in text or/and tablesc of any quality assessment (see also subitems 13aec):<br>➓ Reporting or/and methodological quality of the included SRs.<br>➓ Inform for the quality of the individual studies that were included in the SRs (report results for sequence generation, allocation concealment, blinding, withdrawals, bias etc.) and for the additional included primary studies.<br>➓ Quality of evidence.	Figure 2-3, Table 2-3
20. Present meta-bias(es)	20	Present results of any assessment of meta-bias(es) (such as publication bias or selective reporting across studies, ROBIS assessment).	5
21. Synthesis of results	21a	Summarize and present the main findings of the overview for benefits and harms. If a quantitative synthesis has been conducted, present each summary measure with a confidence interval, prediction interval, or a credible interval and measures of heterogeneity or inconsistency.	5
	21b	Give results of any additional analyses, if done (such as sensitivity, subgroup analyses, or meta-regression).	5
	21c	Report results for adverse events separately for each intervention.	n/a
**Discussion**			
22. Summary of evidence	22	Provide a concise summary of the main findings with the strengths and shortcomings of evidence for each main outcome.	5-6

23. Limitations	23a	Discuss limitations of either the overview or included studies (or both) (e.g., different eligibility criteria, limitations of searching reviews, language restrictions, publication and selection bias).	6
	23b	Report possible limitations of the included reviews related to harms (issues of missing data and information, definitions of harms, rare adverse effects).	6
24. Conclusions	24a	Provide a general interpretation of the results in coherence with the review findings and present implications for practice; consider the harms equally as carefully as the benefits and in the context of other evidence.	6-7
	24b	Present implications for future research.	6-7
**Authorship**			
25. Contributions of authors	25	Provide contributions of authors.	Full title page
26. Dual (co-)authorship	26	Report about dual (co-)authorship in the limitation or declarations of interest section.	Supplementary Material
**Funding**			
27. Funding or other support	27a	Indicate sources of financial and other support for the OoSRs (direct funding) or for the authors (indirect funding), or report no funding.	Full title page
	27b	Provide name for the overview funder and/or sponsor, or for the authors' supporters.	Full title page
	27c	Describe roles of funder(s), sponsor(s), and/or institution(s), if any, in conducted the OoSRs.	Full title page

Note: Extracted from Bougioukas KI, Liakos A, Tsapas A, Ntzani E, Haidich AB. Preferred reporting items for overviews of systematic reviews including harms checklist: a pilot tool to be used for balanced reporting of benefits and harms. J Clin Epidemiol. 2018 Jan;93:9-24. doi: 10.1016/j.jclinepi.2017.10.002. Epub 2017 Oct 14. PMID: 29037888.
